# *Homo sapiens* reached the higher latitudes of Europe by 45,000 years ago

**DOI:** 10.1038/s41586-023-06923-7

**Published:** 2024-01-31

**Authors:** Dorothea Mylopotamitaki, Marcel Weiss, Helen Fewlass, Elena Irene Zavala, Hélène Rougier, Arev Pelin Sümer, Mateja Hajdinjak, Geoff M. Smith, Karen Ruebens, Virginie Sinet-Mathiot, Sarah Pederzani, Elena Essel, Florian S. Harking, Huan Xia, Jakob Hansen, André Kirchner, Tobias Lauer, Mareike Stahlschmidt, Michael Hein, Sahra Talamo, Lukas Wacker, Harald Meller, Holger Dietl, Jörg Orschiedt, Jesper V. Olsen, Hugo Zeberg, Kay Prüfer, Johannes Krause, Matthias Meyer, Frido Welker, Shannon P. McPherron, Tim Schüler, Jean-Jacques Hublin

**Affiliations:** 1 Chair of Paleoanthropology, CIRB (UMR 7241–U1050), Collège de France, Paris, France; 2 Max Planck Institute for Evolutionary Anthropology, Leipzig, Germany; 3 Friedrich-Alexander-Universität Erlangen-Nürnberg, Institut für Ur- und Frühgeschichte, Erlangen, Germany; 4 Ancient Genomics Lab, Francis Crick Institute, London, UK; 5 Department of Molecular and Cell Biology, University of California Berkeley, Berkeley, CA USA; 6 Department of Anthropology, California State University Northridge, Northridge, CA USA; 7 School of Anthropology and Conservation, University of Kent, Canterbury, UK; 8 Univ. Bordeaux, CNRS, Ministère de la Culture, PACEA, UMR 5199, Bordeaux, France; 9 Archaeological Micromorphology and Biomarker Lab, University of La Laguna, San Cristóbal de La Laguna, Spain; 10 Center for Protein Research, University of Copenhagen, Copenhagen, Denmark; 11 College of Earth and Environmental Sciences, Lanzhou University, Lanzhou, China; 12 Globe Institute, University of Copenhagen, Copenhagen, Denmark; 13 Departament de Prehistòria, Universitat Autònoma de Barcelona, Barcelona, Spain; 14 Department of Soil Protection and Soil Survey, State Authority for Mining, Energy and Geology of Lower Saxony (LBEG), Hannover, Germany; 15 Terrestrial Sedimentology, Department of Geosciences, University of Tübingen, Tübingen, Germany; 16 Department of Evolutionary Anthropology and Human Evolution and Archaeological Sciences (HEAS), University of Vienna, Vienna, Austria; 17 Institute of Ecology, Leuphana University, Lüneburg, Germany; 18 Historical Anthropospheres Working Group, Leipzig Lab, Leipzig University, Leipzig, Germany; 19 Department of Chemistry G. Ciamician, Bologna University, Bologna, Italy; 20 Ion Beam Physics, ETH Zurich, Zurich, Switzerland; 21 Landesamt für Denkmalpflege und Archäologie Sachsen-Anhalt – Landesmuseum für Vorgeschichte, Halle, Germany; 22 Department of Physiology and Pharmacology, Karolinska Institutet, Stockholm, Sweden; 23 Thuringian State Office for the Preservation of Historical Monuments and Archaeology, Weimar, Germany

**Keywords:** Population genetics, Archaeology, Anthropology

## Abstract

The Middle to Upper Palaeolithic transition in Europe is associated with the regional disappearance of Neanderthals and the spread of *Homo sapiens*. Late Neanderthals persisted in western Europe several millennia after the occurrence of *H.* *sapiens* in eastern Europe^1^. Local hybridization between the two groups occurred^2^, but not on all occasions^3^. Archaeological evidence also indicates the presence of several technocomplexes during this transition, complicating our understanding and the association of behavioural adaptations with specific hominin groups^4^. One such technocomplex for which the makers are unknown is the Lincombian–Ranisian–Jerzmanowician (LRJ), which has been described in northwestern and central Europe^5–8^. Here we present the morphological and proteomic taxonomic identification, mitochondrial DNA analysis and direct radiocarbon dating of human remains directly associated with an LRJ assemblage at the site Ilsenhöhle in Ranis (Germany). These human remains are among the earliest directly dated Upper Palaeolithic *H.* *sapiens* remains in Eurasia. We show that early *H.* *sapiens* associated with the LRJ were present in central and northwestern Europe long before the extinction of late Neanderthals in southwestern Europe. Our results strengthen the notion of a patchwork of distinct human populations and technocomplexes present in Europe during this transitional period.

## Abstract (web summary)

Through archaeological excavation, morphological and proteomic taxonomic identification, mitochondrial DNA analysis and direct radiocarbon dating of human remains, a study reports the presence of *Homo sapiens* in Germany north of the Alps more than 45,000 years ago.

## Main

**Fig. 1:**
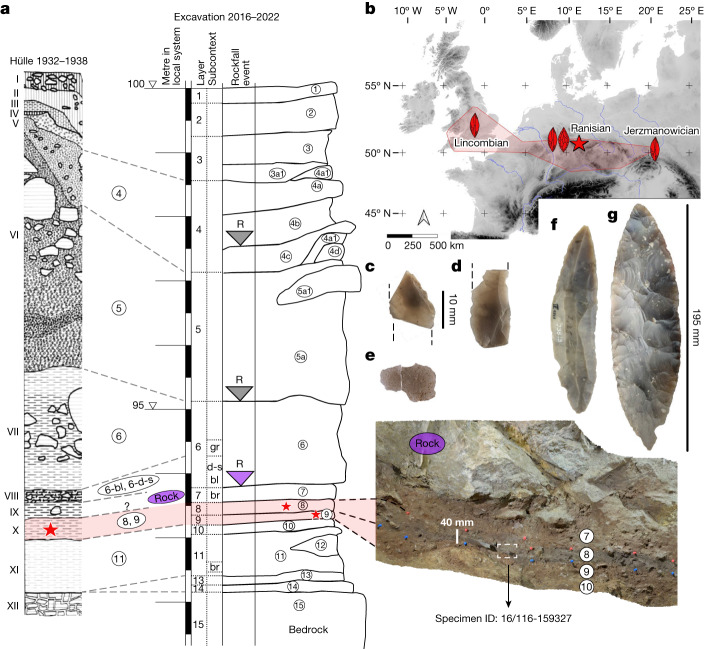
Stratigraphy with location of *H.* *sapiens* bones, map of LRJ sites and lithics from Ranis. **a**, General stratigraphy and correlations of the 1930s and 2016–2022 excavations with the in situ location of the hominin specimen ID 16/116-159327 within the layer 8, north profile (photograph). Stars mark the layers with hominin bones. Shaded in red are the LRJ layers. ‘R’ marks rockfall events. The purple rock represents the 1.7-m-thick rock that sealed the basal sequence. gr, grey; d-s, dark-spotted; bl, black; br, brown. **b**, Location of Ranis (star) and the LRJ (red shaded area). Schematic artefacts mark the dominant leaf point type in the LRJ subdivisions (Lincombian (blade points), Ranisian (blade points and large bifacial points) and Jerzmanowician (blade points)). The map was created in QGIS^39^ on the basis of Shuttle Radar Topography Mission data V4 (http://srtm.csi.cgiar.org)^40^. **c**,**d**, Blade fragments (16/116-159048 and 16/116-151453), layer 8. **e**, Quartzite flake (16/116-159051) from surface retouch, layer 8. **f**, Jerzmanowice blade point, layer X (Museum Burg Ranis, IV 1328). **g**, Bifacial leaf point (Museum Burg Ranis IV 1319), layer X. **a**, Adapted from ref. ^17^. **f**,**g**, Photos: J. Schubert.

The Middle to Upper Palaeolithic LRJ^9,10^ technocomplex extends across northwestern and central Europe (Fig. 1b and Supplementary Fig. 1a). It has been attributed to either Neanderthals^5^ or *H.* *sapiens*^11^. Based on its stone tools, the LRJ has often been classified as Early Upper Palaeolithic^12^ given the laminar blank production directed towards the production of partial-bifacial blade points (Jerzmanowice points)^12^. By contrast, the LRJ has alternatively been interpreted as a local development by Neanderthals^5,10^, as the bi-directional blade production differs from the predominantly uni-directional blade production system of the succeeding Upper Palaeolithic made by *H.* *sapiens*. Additionally, the occasional presence of bifacial leaf points in some LRJ assemblages suggested a Middle Palaeolithic origin. From a chronological perspective, either attribution is possible, as LRJ assemblages are generally dated to about 44,000–41,000 calibrated years before the present (cal bp)^5,13^, a period during which Neanderthals and *H.* *sapiens* groups are known to be present in Europe^14–16^.

**Extended Data Fig. 1:**
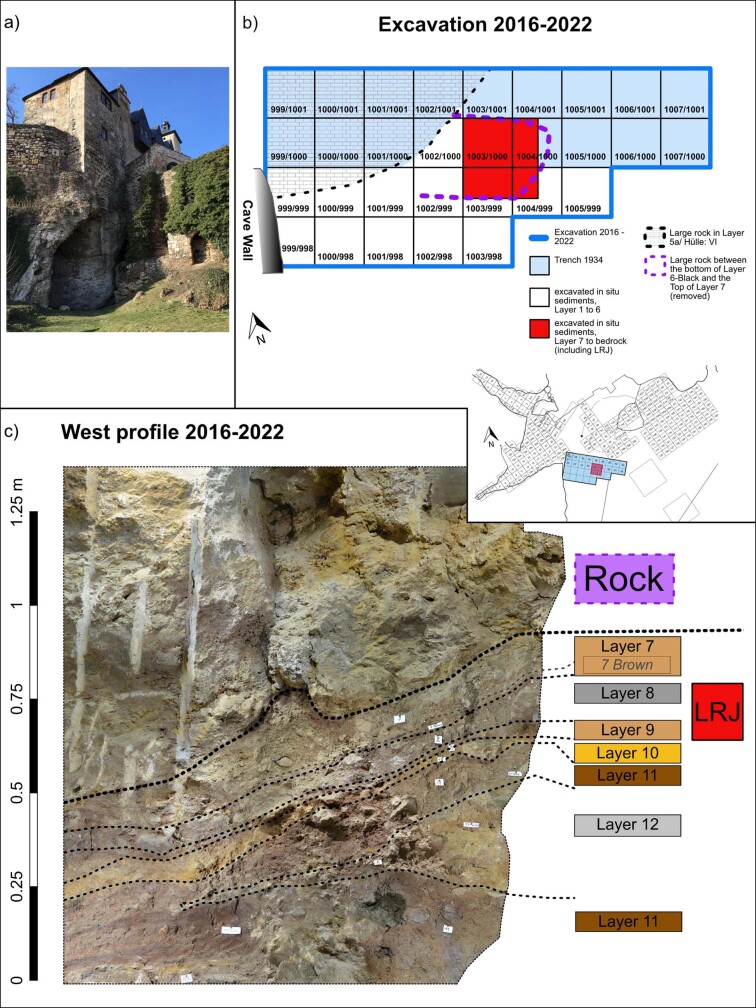
Ranis site plan and main West profile of the 2016–2022 excavation. **a)** Ilsenhöhle Ranis. **b)** Plan of the 2016–2022 excavation. The basal sequence including the LRJ layers were excavated in the red area of squares 1003/999, 1003/1000, 1004/999, and 1004/1000. The purple dashed line marks the removed rock. The inset shows the location of our excavation area relative to the plan of the 1932–1938 excavation^17^, while the red box marks the area of the excavated basal sequence. **c)** West profile of Layers 12 to 7. The lower part of the large rock that was removed is on top of Layer 7. **b** (inset), adapted from ref. ^17^.

**Extended Data Fig. 2:**
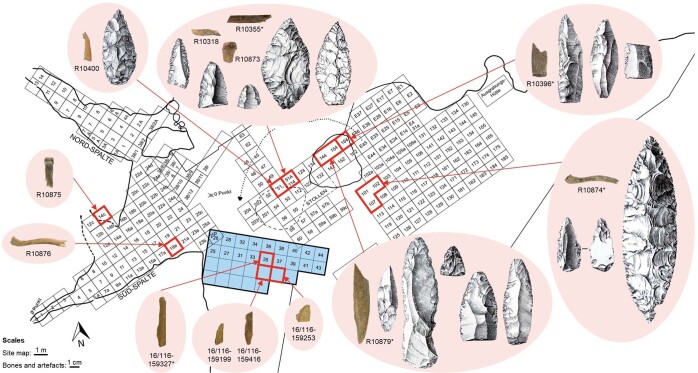
Map of Ranis with the location of the newly identified hominin specimens and selected lithic artefacts. The numbered squares were excavated by W.M. Hülle in the 1930s and we excavated the blue area between 2016 and 2022. The human specimen IDs are provided on the figure (Tab. S5, SI); the five specimens with an asterisk next to their ID showed no pairwise differences in their mtDNA. The artefact IDs are as follows (from left to right when more than one is present in the same square): sq. 51 – 2, 46 ^B^; sq. 101/107 – 2, 31 ^pb^, 2, 56 ^B^; sq. 51 A/114 – 2, 32 ^J^, 2, 36 ^J^, 2, 37 ^J^, 2, 52 ^B^, 2, 54 ^B^; sq. 144/154 – 2, 24 ^J^, 2, 29 ^pb^, 2, 42 ^B^, 2, 43 ^J^, 2, 59 ^J^; sq. 164 – 2, 26 ^J^, 2, 60 ^J^, 2, 78 ^b^. Artefacts marked with a ^B^, ^J^, ^pb^ and ^b^ are bifacial leaf points, Jerzmanowice points, pointed blades and a blade, respectively. The illustrated lithic artefacts were all found in the same layers and on the same day as or within a day of the excavation of the newly identified human remains from the same squares. Site map and artefact drawings are modified from Hülle^17^.

The site Ilsenhöhle in Ranis (50° 39.7563′ N, 11° 33.9139′ E, hereafter Ranis) is one of the eponymous LRJ sites based on its unique composition of bifacial and unifacial points. Ranis is located in the Orla River valley (Thuringia, Germany; Fig. 1b and Supplementary Fig. 1a). The cave formed in the south-facing cliff of a Permian limestone reef (Extended Data Fig. 1a and Supplementary Fig. 2a). Only two short chambers remain intact from a formerly large and high chamber that collapsed during the late Pleistocene^17^. Fieldwork started in 1926, continuing in 1929 and 1931, but the site was mainly excavated by W. M. Hülle between 1932 and 1938 (Extended Data Figs. 1b and 2 and Supplementary Fig. 3; ref. ^17^). Near the base of the 8-m sequence, these excavations revealed a complex stratigraphy of five layers (from bottom to top: XI to VII), including a layer (variably named X and Graue Schicht; Fig. 1a) rich in bifacial leaf points (Fig. 1g and Supplementary Fig. 2c) and with Jerzmanowice blade points (Fig. 1f and Supplementary Fig. 2d). This layer represents the Ranisian as part of the LRJ.

In 2016, we returned to Ranis to clarify the stratigraphy and chronology^18^ and to identify the makers of the LRJ. We reopened the main 1934 trench and excavated adjacent squares to bedrock (Extended Data Fig. 1b and Supplementary Figs. 2b and 3–8). Among the basal layers, layer 11 (Hülle: XI, Fig. 1a) has a low density of undiagnostic, possibly Middle Palaeolithic artefacts^17^. The overlying layer 10 has no equivalent in the 1932–1938 stratigraphy and is followed by layers 9 and 8, which we correlate with the LRJ layer X or Graue Schicht of the Hülle excavation (Fig. 1a). The following layer 7 is sealed by a roof collapse event. A large rock of 1.7 m thickness (Extended Data Fig. 1c and Supplementary Figs. 6 and 8) separates layer 7 from layer 6-black/dark spotted (Hülle: VIII), which contains younger Upper Palaeolithic artefacts. This large rock prevented Hülle from excavating the key basal layers in this location (Extended Data Fig. 1b).

The correlation of our layers 9–8 with the LRJ layer X of the Hülle excavation is based on an extensive set of radiocarbon dates from both collections, ancient DNA analysis, sedimentology and micromorphology showing anthropogenic inputs of charred plant material (Supplementary Fig. 9), and lithic artefact analysis. Like layer X (ref. ^17^), layer 8 has the highest lithic density (Supplementary Tables 2 and 3) of the basal stratigraphic sequence below the Upper Palaeolithic (layer 6-black/dark spotted) and represents the main occupation of the LRJ. Although we did not recover any diagnostic points, two of the artefacts from our excavations are fragmented blades (Fig. 1c,d), which are typical blanks for the LRJ blade points. Similar to the LRJ assemblage from layer X (ref. ^17^), most of the artefacts from layers 9 and 8 were made of Baltic flint (Supplementary Table 3). This shows a connection of the Ranis LRJ to the lowlands north of the site where the flint was procured. Three small flakes coming from surface shaping and edge retouch are made of quartzite (Fig. 1e). This raw material also occurs in a few artefacts from the 1932–1938 excavation (Supplementary Table 1). Among them is a fragment of a bifacial tool, possibly a leaf point. Except for one chunk, layer 7 contained no artefacts, but a few flint chips were found during sorting of screened excavated sediment from the layer contact 8/7 (Supplementary Table 3). We assign these artefacts to the archaeological horizon of layers 9–8. In contrast to the LRJ layers, flint artefacts are absent in the underlying layers 10 and 11. A similar low-frequency usage of flint has also been reported for layer XI of the 1932–1938 excavation^17^.

**Fig. 2:**
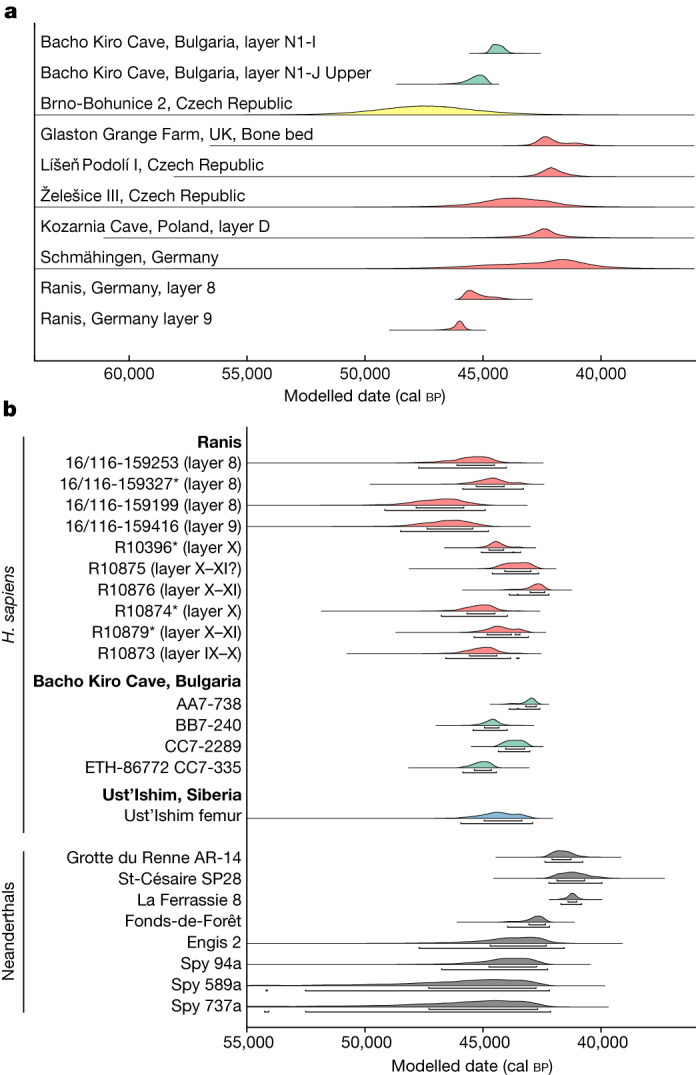
Chronological comparison of Ranis with selected contemporary sites and directly dated human remains. **a**, Distributions showing kernel density estimates^41^ of radiocarbon dates from LRJ contexts across Europe (red), IUP contexts at Bacho Kiro Cave (green) and TL dates from Brno-Bohunice (yellow). The location of the sites is shown in Supplementary Fig. 1. **b**, Calibrated ranges of directly radiocarbon dated *H.* *sapiens* from Ranis (LRJ, red), Bacho Kiro Cave (IUP, green) and Ust’-Ishim (no associated archaeology, blue), and Neanderthals (grey) from southwestern France and Belgium. The asterisk marks the Ranis bones for which mtDNA indicates that they originate either from the same or maternally related individuals. The four dates are statistically identical. R10355 and R10318 were not directly dated owing to contamination from conservative treatments (Supplementary Information section 4.1). Layer numbers of the bones from the 1932–1938 excavation (IX, X and XI) refer to labels of boxes in which the finds are stored, which contain material from one or more layers. Data included are shown in Supplementary Tables 17 and 26.

**Extended Data Fig. 3:**
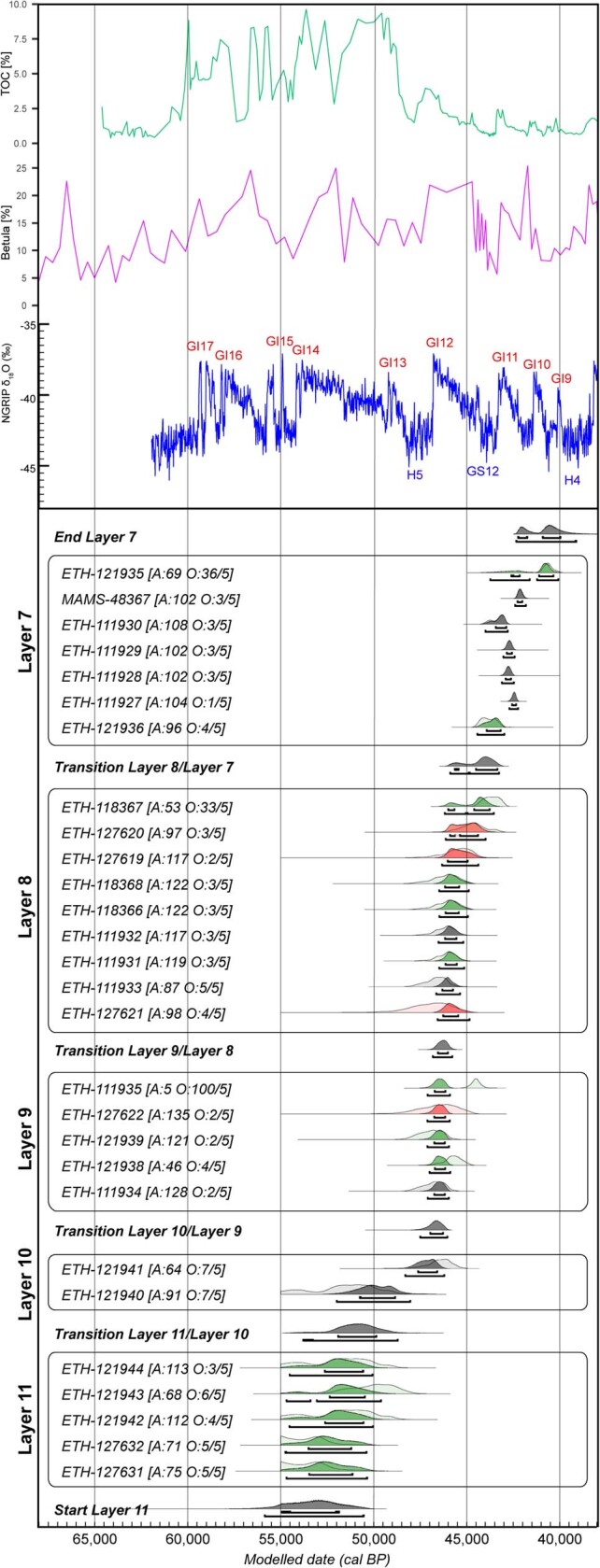
Chronological site model of 2016-2022 material from Ranis. Directly dated human remains are shown in red, and faunal bones with anthropogenic modifications are shown in green. Along the top of the figure, the North Greenland Ice Core Project (NGRIP) GICC05 δ^18^O curve (dark blue) is shown with warmer Greenland Interstadials (GI17-5) and cooler Greenland Stadial 12 (GS12) and Heinrich Events (HE5, HE4) indicated^79,80^. Climatic cooling effects correlating with GS12 can also be seen in German palaeoclimate records closer to Ranis: the purple curve shows the Betula pollen (%) measured in the Füramoos pollen record from southern Germany^81^ and the green curve shows the Total Organic Carbon Content (TOC) in the Eifel maar sediment core in southwestern Germany^82^. Given the errors inherent in the ^14^C chronology and climate records themselves, any correlations noted here are tentative^83^. The model outputs are shown in Tab. S15, SI and further information is in SI section 4.2.

We constructed a chronological model based on 28 radiocarbon dates from newly excavated material from layers 11–7, including directly dated human remains, anthropogenically modified bones and charcoal. Bone collagen preservation was exceptional with an average yield of 11.8% (range: 5.3–16.3%, *n* = 33; Supplementary Table 13). Only one date in the model was identified as an outlier, highlighting the stratigraphic integrity of the layers. At the base, layer 11, containing undiagnostic artefacts, dates to 55,860–48,710 cal bp. Layers 9 and 8, associated with the LRJ, date to 47,500–45,770 cal bp and 46,820–43,260 cal bp, respectively (modelled ranges at 95.4% probability; Extended Data Fig. 3 and Supplementary Table 15). The overlying layer 7 dates to 45,890–39,110 cal bp and is sealed by the roof collapse. In addition, six newly identified human bones from layer X in the 1930s collection, thought to be associated with the LRJ, were directly radiocarbon dated (see below). These dates (46,950–42,200 cal bp at 95.4% probability) fit within the range of dates obtained in our model for layers 9 and 8 (Fig. 2 and Extended Data Fig. 3), thus providing additional support for linking the LRJ of layer X (1930s) with our layers 9 and 8.

We carried out a combination of two proteomic screening approaches (matrix-assisted laser desorption ionization–time-of-flight mass spectrometry and liquid chromatography–tandem mass spectrometry) and morphological identification on bone specimens from the 2016–2022 and 1932–1938 excavations (Methods). We were able to retrieve 13 hominin bone specimens in total (Extended Data Table 1). Out of those, four hominin bones were discovered through proteomic methods and derived from the 2016–2022 excavation: one from layer 9 (specimen ID: 16/116-159416) and three from layer 8 (specimen IDs: 16/116-159253, 16/116-159327 and 16/116-159199; Fig. 1a). Among material from the 1932–1938 excavation, we identified nine additional hominin bone specimens, four through proteomic analysis, all from layer X (specimen IDs: R10318, R10355, R10396 and R10400), and five through morphological analysis from boxes labelled with layer X, layers IX and X, or layers X and XI (specimen IDs: R10873, R10874, R10875, R10876 and R10879; Fig. 1a and Supplementary Table 5). The boxes with mixed layer tags from the 1932–1938 excavation are a result of the expedited excavation methods from the 1930s. The hominin remains were in most cases, however, excavated on the same day or within a day of the discovery of LRJ artefacts in the same squares (Extended Data Fig. 2).

**Extended Data Fig. 4:**
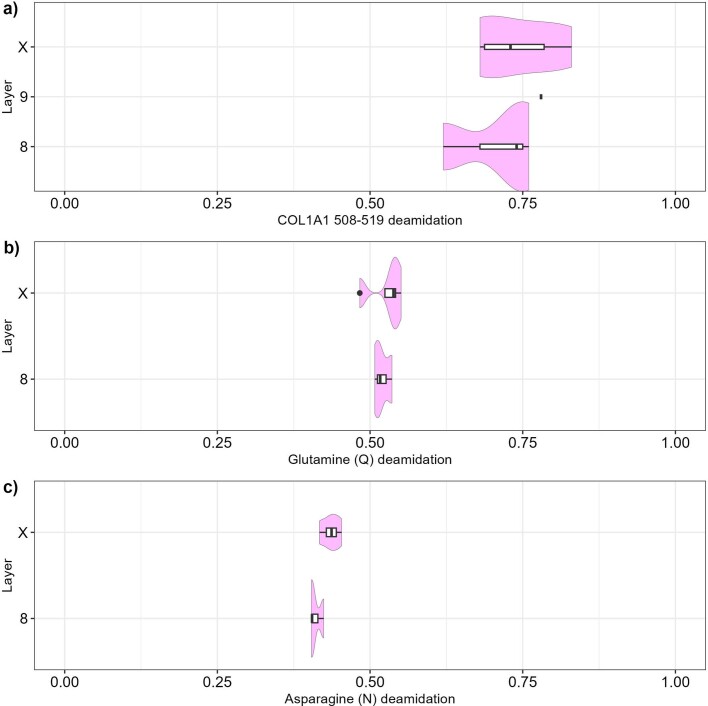
Protein deamidation for all hominin specimens in Ranis. **a)** Deamidation rate of COL1A1 508–519 for hominin specimens in the 2016–2022 and 1932–1938 excavations with ZooMS (layer X, *n* = 4 NISP; layer 8, *n* = 3; layer 9, *n* = 1), **b)** Glutamine (Q) deamidation plot for hominin specimens in the 2016–2022 and 1932–1938 excavations analysed with directDIA SPIN (layer X, *n* = 4; layer 8, *n* = 3), and **c)** Asparagine (N) deamidation plot for hominin specimens in the 2016–2022 and 1932–1938 excavations analysed with directDIA SPIN (layer X, *n* = 4; layer 8, *n* = 3). The hominin specimen recovered from layer 9 was not included in the SPIN analysis (plots b and c). For panel a, 1 indicates no deamidation of N or Q, while 0 indicates complete deamidation of N or Q. For panels b and c, 0 indicates no deamidation of N or Q, while 1 indicates complete deamidation of N or Q. The box plots within the violin plots define the range of the data (whiskers extend to 1.5× the interquartile range), outliers (black dots, beyond 1.5× the interquartile range), 25th and 75th percentiles (boxes), and medians (centre lines).

**Extended Data Fig. 5:**
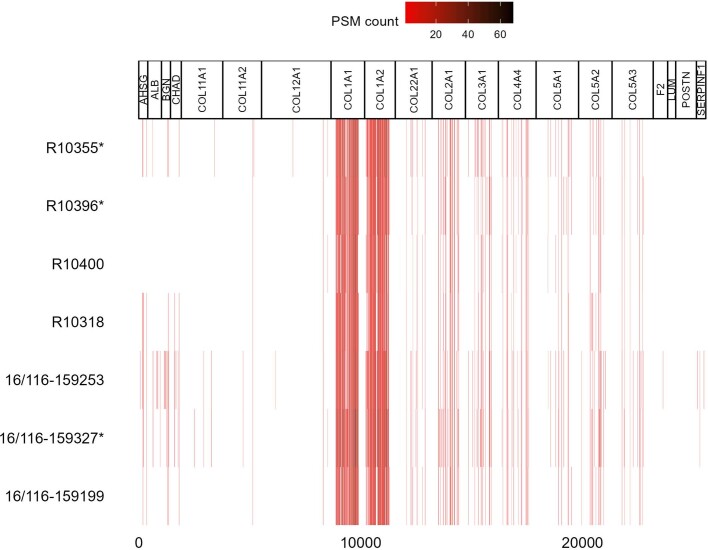
Proteomic coverage for the seven hominin bone specimens analysed with SPIN. Peptide spectrum match (PSM) count per protein amino acid for each hominin specimen by libraryDIA of the 20 genes used in Rüther et al. ^3^. White indicates no coverage, red indicates medium coverage, and black indicates high coverage. The asterisk (*) marks the specimens where mtDNA indicates they originate from the same or maternally related individuals. The x-axis represents the amino acid position in the protein sequence alignment of the 20 proteins considered in SPIN analysis.

To support the identification of endogenous proteomes in all proteomically identified hominin specimens, we evaluated the amino acid degradation and their coverage per identified position. Proteome deamidation measurements (matrix-assisted laser desorption ionization–time-of-flight and liquid chromatography with tandem mass spectrometry) of the fauna revealed diagenesis of collagen type I consistent with all of the hominin remains (Extended Data Fig. 4 and Supplementary Fig. 10). Finally, we compared our results with the existing reference proteome of Homininae^19^ and calculated the proteomic coverage per amino acid position for all of the identified hominin specimens (Extended Data Fig. 5). Our proteomic sequencing results showed that amino acid positions recovered for all hominin specimens analysed with the species by proteome investigation pipeline^19^ matched with the Homininae reference proteome. However, owing to limitations in the existing proteomic reference database^19^, further taxonomic distinctions among hominin populations are not made.

**Fig. 3:**
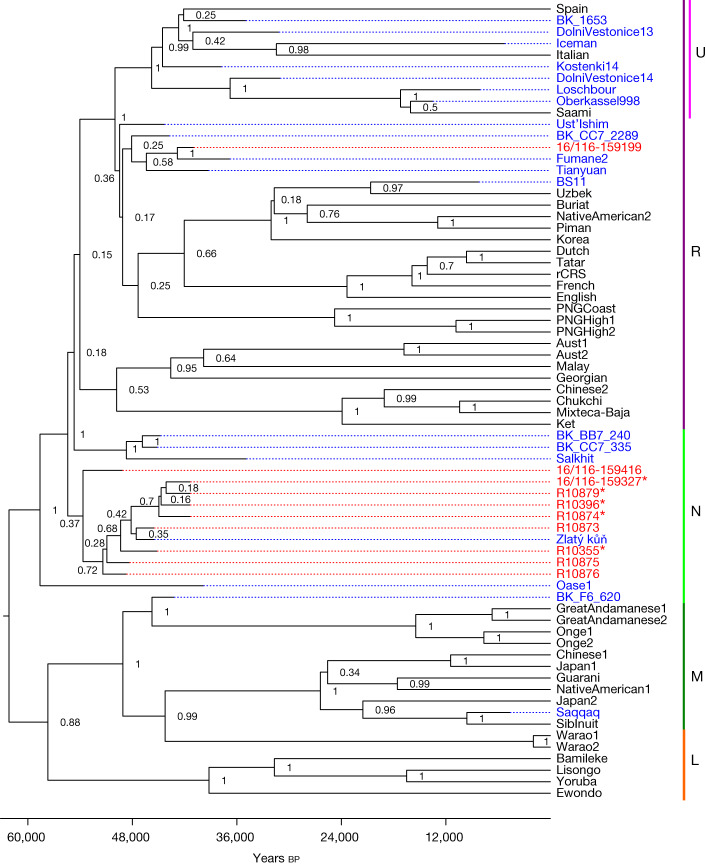
Bayesian phylogenetic tree of the newly reconstructed mtDNA genomes with previously published ancient and recent modern human mtDNA genomes constructed with BEAST2. The posterior probability is shown for each branch point and the *x* axis shows the years before present. Individual genomes are coloured to denote whether they are ancient (blue) or modern (black) genomes; newly sequenced mtDNA genomes from this study are coloured in red. The Neanderthal mtDNA genomes used to root the tree and modern human mtDNA genomes falling outside the clades shown are not depicted. Asterisks mark mtDNA genomes with no pairwise differences. MtDNA haplogroups (L, M, N, R and U) are labelled in the right column.

We tested 11 of the hominin remains for the preservation of ancient mitochondrial DNA (mtDNA). Between 4,413 and 175,688 unique reads mapping to the human mtDNA reference genome were recovered per skeletal fragment. These mtDNA reads had elevated frequencies of cytosine (C)-to-thymine (T) substitutions (32.6% to 49.6% on the 5′ end and 19.0% to 47.9% on the 3′ end, respectively; Supplementary Figs. 14–24), which are indicative of ancient DNA. Positions shown to be informative for differentiating between *H.* *sapiens*, Neanderthal and Denisovan mtDNA genomes enabled us to identify each of the 11 skeletal fragments as belonging to ancient *H.* *sapiens* (Supplementary Table 18). Libraries from ten of the eleven skeletal fragments contained sufficient data for reconstructing near-complete mtDNA genomes. Five of these mtDNA genomes showed no pairwise differences among them for the positions covered, suggesting that they stemmed from either the same individual or maternally related individuals (Supplementary Fig. 25 and Supplementary Table 19). Four of these skeletal fragments come from the 1932–1938 collection and one from the 2016–2022 excavation (16/116-159327; Fig. 1), providing additional support to the correlation of layers 9 and 8 with layer X. Four of these fragments (16/116-159327 from the 2016–2022 excavation; R10874, R10879 and R10396 from the 1930s collection) also produced statistically indistinguishable radiocarbon dates (Fig. 2). The morphology and stable isotopic values^20^ of R10874 suggest that it originates from a different individual, consistent with a maternal relation. Notably, whereas nine of the ten reconstructed mtDNA genomes belonged to haplogroup N, one (16/116-159199) was identified as belonging to haplogroup R. When placed onto a phylogenetic tree with other ancient humans, the mtDNA genomes with an N haplogroup cluster together with the mtDNA genome of Zlatý kůň, an individual from the Czech Republic, whose chronological age is around 45,000 years before present on the basis of genetic estimates^3^ (Fig. 3 and Supplementary Figs. 26 and 27). The estimated mean genetic dates of the Ranis mtDNA genomes ranged from 49,105 to 40,918 years before present (Supplementary Table 22 and Supplementary Fig. 26), consistent with the radiocarbon dates from layers 9 and 8.

The hominin remains from the 2016–2022 and 1932–1938 excavations are associated with a range of animal taxa (Supplementary Table 9). Overall, both zooarchaeological and proteomic analyses identified a total of 17 taxa with a predominance of reindeer (*Rangifer tarandus*). Other taxa included Bovinae (*Bos primigenius* and *Bison priscus*), Cervidae (*Cervus elaphus*), horse (*Equus ferus*) and megafauna (*Coelodonta antiquitatis* and *Mammuthus primigenius*). A variety of carnivores were also identified, dominated by cave bear (*Ursus spelaeus*; Supplementary Table 9). This species composition is consistent with the faunal record of central Europe during Marine Isotope Stage 3 (refs. ^21–24^). Our analyses suggest that large carnivores accumulated most bone remains with only occasional, short-term site use by human groups (Supplementary Tables 10–12), which is consistent with the recovered ancient sediment DNA and the relatively low lithic counts (Supplementary Tables 1–3) in these LRJ layers^20^. This is similar to what has been observed at other LRJ sites^25^. Our sedimentological analyses indicate a temperature decline from layer 9 towards colder climatic conditions in layer 7 (Supplementary Table 4). This agrees with stable isotope analyses of equid teeth that indicate a temperature decline with low temperatures and an open steppe environment during all phases of the LRJ occupations. Temperatures reconstructed for the coldest phase, about 45,000–43,000 cal bp (overlapping with both layer 8 and layer 7), were 7–15 °C lower than those of the modern day and are consistent with a highly seasonal subarctic climate in full stadial conditions^26^. On the basis of comparisons with the timing of Greenland stadials and Greenland interstadials in both the North Greenland Ice Core Project and terrestrial sequences in western Germany, the LRJ occupations overlap with a variety of climatic phases including Greenland Stadial 13 (GS13), Greenland Interstadial 12 and GS12, and a temperature decline towards full stadial conditions during the coldest phase is congruent with an interstadial–stadial transition culminating in a pronounced cold phase such as GS12 or GS13 (Extended Data Fig. 3).

In summary, our work shows that the LRJ at Ranis was made by hominins with *H.* *sapiens* mtDNA. This indicates that pioneer groups of *H.* *sapiens* expanded rapidly into the higher mid-latitudes, possibly as far as the modern day British Isles (Fig. 1b), before much later expansions into southwestern Europe, where directly dated Neanderthal remains are documented until about 42,000 cal bp (Fig. 2). Although non-directly dated and non-genetically identified, a human deciduous tooth from Grotte Mandrin^27^ also suggests an *H.* *sapiens* incursion into southeastern France as early as about 54,000 cal bp. If confirmed, this evidence would create a complex mosaic picture of Neanderthal and *H.* *sapiens* groups in Europe between about 55,000 and 45,000 cal bp. On the basis of the archaeological and zooarchaeological evidence, the pioneer *H.* *sapiens* groups were small and possibly left no notable genetic traces in the later Upper Palaeolithic hunter-gatherers in Europe^28^. The early presence of *H.* *sapiens* in the modern day British Isles is further evidenced by the disputed dating of the Kent’s Cavern maxilla^7,8,29,30^, probably associated with LRJ stone tools at this site. Archaeological and mtDNA data further suggest that LRJ *H.* *sapiens* at Ranis were connected to populations of eastern and central Europe. The relationship between the bifacial-point-rich LRJ at Ranis and other chronologically overlapping bifacial point industries of central Europe, such as the Szeletian^31,32^ and Altmühlian^33,34^ (Supplementary Fig. 1b), remains to be explored. If the Initial Upper Palaeolithic (IUP) Bohunician^35^ of Moravia and the LRJ are related technocomplexes^36^, then the LRJ is part of the IUP expansion into Europe. There are no human remains preserved from the Bohunician, but the Zlatý kůň *H.* *sapiens* skull from the Czech Republic overlaps with the dates for both the Bohunician^35,37^ and the LRJ at Ranis. Notably, nine out of ten Ranis mtDNA genomes cluster with the Zlatý kůň individual and one clusters with the Fumane 2 individual, both of whom are other early *H.* *sapiens* individuals who lived around the same time in Europe as the Ranis specimens described here. This connects the LRJ hominins to a wider population network of initial *H.* *sapiens* incursions into Europe. Finally, the demonstration that the LRJ was produced by *H.* *sapiens* fills an important gap in the record of the last Neanderthals and *H.* *sapiens* in northwestern and central Europe around 45,000 cal bp. The hypothesis that Neanderthals disappeared from northwestern Europe well before the arrival of *H.* *sapiens*—which is largely based on the chronological hiatus observed between Neanderthal-made late Middle Palaeolithic assemblages and *H.* *sapiens*-made Aurignacian assemblages^38^—can now be rejected.

## Methods

### Excavation methods

We located and reopened 18 squares on the grid line of the main east–west trench from 1934 and excavated an 8-m-deep sequence of 12 adjacent squares (Extended Data Fig. 1b and Supplementary Figs. 2b and 3–8). Layers were identified using lithological as well as archaeological criteria, if available. Stratigraphic units were subsequently numbered from top to bottom, independently of the 1932–1938 excavation^17^. The lower layers, from layer 6 to bedrock, were excavated over 2.25 m^2^. Large rocks within the sequence (that is, collapsed parts of the former cave roof) prevented us from enlarging the excavation. To access the deeper stratigraphy that includes the transition period of layers 9 and 8 (Hülle: X), we removed a 1.7-m-thick rock (Extended Data Fig. 1c and Supplementary Figs. 6 and 8) that separated these layers from the overlying Upper Palaeolithic layer 6-black/dark spotted (Hülle: VIII)^17^. The same rock extended into squares 35 and 37 from the Hülle excavation^17^. Hülle did not remove this rock and never excavated below it. We therefore incorporated these two squares below the rock into our excavation (1003/1000 and half of 1004/1000; Extended Data Fig. 1b). Although rock removal required substantial efforts, the rockfall provided an ideal situation for the underlying sequence, as it was sealed from post-depositional disturbances by geological processes, humans and animals.

Owing of the depth of the sequence, we secured the walls with wooden planks and metal poles during the progress of the excavation. This work was carried out professionally (by a construction firm) and inspected. Before the walls were closed and secured, all profiles were documented (see below).

We excavated using current standards for Palaeolithic sites. We established an arbitrary excavation grid oriented along the former east–west trench of the 1932–1938 excavation, which was later georeferenced in the global Universal Transverse Mercator coordinate system. We removed sediments in 10-l buckets separated by layer. We recorded the location of each bucket with two sets of three-dimensional (3D) coordinates, one at the beginning and one at the end of each bucket. The sediments were then wet-screened through 4-mm and 2-mm meshes. Single finds >20 mm (lithics and fauna) and samples (sediment and micromorphology) were assigned unique IDs; their layer, date of excavation and excavator were recorded, and 3D coordinates were measured using a Leica total station (5” accuracy). The total station measurement and attribute recording were carried out with EDM-mobile, a self-authored software. At the end of each day, we transferred these data to the primary database for the project. Objects with an identifiable long axis were measured with two coordinates at their endpoints to record bearing and plunge. Stones >10 mm were measured with one coordinate at the base and stones >20 mm were recorded with six coordinates (representing the three axes of the object) to document volume and orientation. Large rocks were documented using multiple measurements.

We documented layers, special features and profiles in highly detailed and precise 3D models using structure from motion (Agisoft Metashape), total station measurements, digital photographs and drawings. The 3D models were georeferenced with control points recorded with the total station to align to the excavation grid.

### Proteomic screening

We proteomically screened 1,322 morphologically unidentified fragmentary bone specimens from layers 7–12 (2016–2022 excavation) and layers IX–XI (1932–1938 excavation) with zooarchaeology by mass spectrometry (ZooMS)^42^ and a subset of 341 bone specimens with the species by proteome investigation (SPIN) workflow^19^. Generally, we targeted specimens >2 cm in length to enable future direct radiocarbon dating and ancient DNA analysis. We measured bone length for most specimens, and recorded anthropogenic, carnivore and taphonomic modifications for all of them. Sampling was carried out using pliers or a dental drill. Negative controls were also included in the study (Supplementary Table 6).

First, all specimens were analysed through ZooMS analysis. A total of 769 specimens were derived from the 1932–1938 excavation (Supplementary Table 24), and 553 specimens derived from the 2016–2022 excavation (Supplementary Table 23). Extraction and analytical protocols followed in ZooMS were previously published^43^. In brief, a small piece of bone (about 5 mg) from each specimen was suspended and denatured in 50 mM ammonium bicarbonate pH 8.0 for 1 h at 65 °C. The samples were digested overnight at 37 °C with 50 mM trypsin solution, acidified using 5% trifluoroacetic acid (TFA) and purified using a HyperSep C18 filter plate (Thermo Scientific). Matrix-assisted laser desorption ionization–time-of-flight MS (MALDI–TOF MS) analysis for the first 649 specimens was conducted at the IZI Fraunhofer in Leipzig, Germany, in an autoflex speed LRF MALDI–TOF (Bruker) with reflector mode, positive polarity and spectra collected in the mass-to-charge range 1,000–3,500 *m*/*z*. The remaining specimens were analysed on a MALDI–TOF 5800AB Sciex instrument at the Ecole Supérieure de Physique et Chimie Industrielle, Paris, France, in positive reflector mode, covering a mass-to-charge range of 1,000 to 3,500 Da. MALDI–TOF MS replicates (*n* = 3) were averaged for each sample and manually inspected for the presence of relevant peptide markers (A–G)^44^ in mMass v5.5.0 (ref. ^45^). MALDI–TOF MS spectra were analysed in comparison to a reference database containing collagen-peptide marker masses of all medium- to larger-sized genera in existence in western Eurasia during the late Pleistocene^42,46,47^. Glutamine deamidation values were calculated using the Betacalc3 package^48^ on the basis of COL1α1 508-519 deamidation.

Second, we proteomically sequenced a subset of 341 fragmentary bone specimens, previously identified by ZooMS, with SPIN^19^. A total of 129 specimens were retrieved during the 1932–1938 excavation and 212 specimens came from the 2016–2022 excavation (Supplementary Table 7). Approximately 5 mg of each bone specimen was suspended and demineralized overnight in 5% hydrochloric  acid (HCl) and a 0.1% nonyl phenoxypolyethoxylethanol (NP-40) solution at room temperature with continuous shaking. Reduction, alkylation and collagen gelatinization were facilitated by adding 0.1 M tris(2-carboxyethyl) phosphine (TCEP) and 0.2 M *N*-ethylmaleimide (NEM) at 60 °C, for 1 h. The protein aggregation capture and digestion took place on a KingFisher Flex (ThermoFisher Scientific) magnetic bead-handling robot. Protein extracts were mixed with magnetic SiMAG-Sulfon beads. Protein aggregation was initiated with the addition of 70% acetonitrile (ACN). The beads and the proteins were washed in 70% acetonitrile, 80% ethanol and 100% acetonitrile consecutively. Then, proteins were released into a solution of 20 mM Tris pH 8.5, 1 µg ml^−1^ LysC and 2 µg ml^−1^ trypsin for proteome digestion. The digestion was finalized outside the robot at 37 °C, overnight. The peptides were acidified with 5% trifluoroacetic acid and purified in a C18 Evotip. The specimens were analysed on an Evosep One (Evosep)^49^ connected in line to an Orbitrap Exploris tandem mass spectrometer (ThermoFisher) at the Centre for Protein Research at the University of Copenhagen, Denmark. The samples were analysed with the 200SPD Evosep One method on a short online liquid chromatography gradient in MS/MS data-independent acquisition (DIA) mode using a homemade 3-μm silica column. Full scans ranged from 350 to 1,400 *m*/*z* and were measured at 120,000 resolution, 45 ms maximum IT, 300% AGC target. Precursors were selected for data-independent fragmentation in 15 windows ranging from 349.5 to 770.5 *m*/*z* and 3 windows ranging from 769.5 to 977.5 *m*/*z*, with 1-*m*/*z* overlap.

DIA MS/MS spectra were loaded into Biognosys Spectronaut^50,51^ v15.6.211220, and analysed using either library-based (libDIA) or library-free DirectDIA (dirDIA) search. The required peptide identification data were generated with a Spectronaut report based on the SPIN.rs^19^ scheme for DIA analysis, and the library-based and the library-free DirectDIA searches were carried out as described in previous studies^19^. The utilized protein sequence databases contained entries for the 20 most common bone proteins from a wide range of species represented in UniProt and Genbank. The species determination was carried out in R v4.1.2 (ref. ^52^) as described previously^19^. Variable modifications included in the search were oxidation (methionine), deamidation (asparagine-N, glutamine-Q), glutamine to pyroglutamic acid, glutamate to pyroglutamate and proline hydroxylation, while NEM derivatization of cysteine was included as a fixed modification. In part, SPIN taxonomic identification was based on gene-wise alignment of protein entries in the searched database and the computation of two quality control markers for estimating the confidence of each taxonomic assignment. As a result, we noted that the SPIN^19^ and ZooMS taxonomic reference databases were composed of partly overlapping but highly different taxonomic entities (Supplementary Table 8), with absences of some taxonomic groups in SPIN resulting in false species assignments despite high-confidence bone proteome data (Supplementary Figs. 11 and 12). The six Ranis specimens subjected to liquid chromatography–MS/MS with data-dependent acquisition were analysed as described previously^53^.

### Morphological identification of hominin remains

All of the faunal remains from the 1932–1938 excavation housed at the Landesamt für Denkmalpflege und Archäologie Sachsen-Anhalt in Halle (Saale) were sorted to potentially identify human remains among them. This consisted of examining each skeletal element of any size and visually assessing characteristics such as overall shape, size, tissue proportions, developmental stage and presence of particular anatomical features to discern the right category for a bone or tooth. Several potentially hominin specimens were isolated in this manner. They come mostly from the upper layers of the site. The human status of those from the lower layers was confirmed through DNA analysis, and they were subsequently directly radiocarbon dated.

### Radiocarbon dating

Radiocarbon dating of 30 samples from layers 11–7 of the 2016–2022 excavation was undertaken to establish a site chronology, including 27 bone specimens and 3 charcoal samples (Supplementary Table 13). The bone specimens included 4 hominin bones and 23 faunal bones (14 of which showed signs of anthropogenic modification including butchery, cut marks and percussion notches). Six hominin bones from the 1930s collections were radiocarbon dated to determine their chronological position in relation to the new site chronology. Two additional hominin bones from the 1930s collection had been conserved with paraffin so they were excluded from dating owing to the risk of contamination (Supplementary Fig. 13 and Supplementary Information section 4.1).

Pretreatment of the bone samples was carried out in the Department of Human Evolution at the Max Planck Institute for Evolutionary Anthropology in Leipzig, Germany. Collagen was extracted from the faunal bone samples using about 300–600 mg material following an acid–base–acid plus ultrafiltration protocol published previously^54,55^, and from the human bones using about 55–160 mg bone material following a previously published protocol for small sample extraction^54^ (Supplementary Information section 4.1). Two faunal bones were pretreated with a second collagen extraction protocol to test for the presence of modern carbon contamination resulting from humic acids (Supplementary Table 14). Suitability for dating was assessed on the basis of the collagen yield (as a percentage of dry bone weight) with a 1% minimum requirement and the elemental values, measured on a ThermoFinnigan Flash elemental analyser coupled to a Thermo Delta plus XP isotope ratio mass spectrometer. To pass the quality threshold, samples were required to fall within the accepted elemental ranges of modern collagen samples (about 35–45% C; about 11–16% N; C/N between 2.9 and 3.6)^56^.

The collagen extracts were graphitized^57^ and dated at the Laboratory for Ion Beam Physics at ETH Zurich, Switzerland on a MICADAS accelerator mass spectrometer (AMS)^58,59^. Sub-samples of two bones that date beyond the limit of the ^14^C method (>50,000 bp) (‘background’ bones) were pretreated and dated alongside the samples to monitor laboratory-based contamination. The accelerator mass spectrometer measurements of the collagen backgrounds were used in the age correction of each batch of samples in BATS^60^, with an additional 1‰ error added, as per the standard practice (Supplementary Table 13).

The three charcoal samples were pretreated at the Curt-Engelhorn-Center for Archaeometry gGmbH (MAMS) using a softened ABOx protocol before being combusted to CO_2_ in an elemental analyser, converted catalytically to graphite and measured on a MICADAS accelerator mass spectrometer^61^. Two of the three charcoal samples had very low percentages of C following combustion and were therefore excluded from the site chronological modelling.

Measured radiocarbon ages are reported in Supplementary Table 13 with the abbreviation bp, meaning radiocarbon years before AD 1950, and are reported with 1*σ* errors, whereas calibrated radiocarbon ranges are denoted as cal bp and are given at the 95.4% (2*σ*) probability range. All ages have been rounded to the nearest 10 yr. Calibration and Bayesian modelling of the radiocarbon dates was carried out in OxCal v4.4 (ref. ^62^) using the IntCal20 calibration curve^63^. A multi-phase model was constructed for the dates from the 2016–2022 excavation using stratigraphic information as a prior. A ‘general’ outlier model was used to assess the likelihood of each sample being an outlier with prior probabilities set to 5%. The date ranges for each layer discussed in the text are the output of the site chronological modelling. Further information and the OxCal code is included in Supplementary Information sections 4.2 and 4.3 and Supplementary Tables 15 and 16.

### Ancient DNA analysis

Eleven skeletal remains were screened for ancient human DNA preservation. Between 6.1 and 63.9 mg of bone powder was drilled from each specimen in a dedicated clean room at the Max Planck Institute for Evolutionary Anthropology in Leipzig, Germany, or the former Max Planck Institute for the Science of Human History in Jena, Germany. DNA extracts were prepared following the protocol described previously^64^ with buffer ‘D’ and subsequently converted into single-stranded and double-indexed libraries following the automated protocol described previously^65^. Libraries from 10 of the 11 remains were subsequently enriched for human mtDNA using the singleplex automated capture protocol described earlier^66^. The resulting libraries were sequenced on either the Illumina NextSeq or MiSeq platforms (2 × 76 cycles). No cross-contamination of DNA molecules due to index hopping was detected in these libraries on the basis of calculations from a previously published method^66^. For one of the specimens, R10873, four single-stranded libraries were prepared from the same extract in the Jena facilities following the protocol described above and pooled together. Shotgun sequencing of the pooled libraries was carried out at the SciLifeLab, on a full Novaseq S4-200 flow-cell using 2 × 75-base-pair paired-end sequencing.

Base calling was carried out with Bustard, and reads were overlap merged using leeHom^67^. Mapping was carried out with BWA^68^ using adjustments for ancient DNA (-n 0.01 –o 2 –l 16500)^69^. All libraries were mapped to the human mtDNA revised Cambridge Reference Sequence (NC_01290)^70^. Reads from the libraries generated from the same skeletal fragment were then merged using Samtools merge^71^. PCR duplicates, reads shorter than 35 base pairs or with a mapping quality less than 25 were removed using bam-rmdup (v0.6.3; https://bitbucket.org/ustenzel/biohazard). Each library was then evaluated for the presence of authentic ancient human DNA (Supplementary Table 25). The proportion of present day human DNA contamination was estimated using AuthentiCT^72^. Support for *H.* *sapiens*, Neanderthal or Denisovan mtDNA among the recovered mtDNA fragments was determined using sets of previously published ‘diagnostic’ positions^73^ that allow differentiation among these hominin mtDNA types (Supplementary Information section 5).

Full mtDNA genomes were reconstructed for 10 of the 11 specimens with >70-fold coverage of the mtDNA genome. Consensus bases were called using either all fragments (for the libraries with the levels of present day human DNA contamination <5%) or deaminated fragments alone (for the libraries with the levels of present day human DNA contamination >5%), requiring each site to be covered with at least five DNA fragments and 80% support while ignoring the C-to-T substitutions on the first and/or the last seven positions from the alignment ends. The haplogroup of each reconstructed mtDNA genome was determined using HaploGrep2 (v2.4.0)^74^. MAFFT (v7.453)^75^ was used to realign all ten newly reconstructed human mtDNA genomes to the rCRS with previously published mtDNA genomes from 54 modern humans, 19 ancient humans and 2 Neanderthals. A phylogenetic tree relating these mtDNA genomes was generated using BEAST2 v2.6.6 (ref. ^76^) with a Bayesian skyline tree model and strict clock model (see Supplementary Information section 5 for model and parameter details; Supplementary Table 21). Molecular dates for the new mtDNA genomes were estimated by calibrating the tree with the mtDNA genomes of individuals with direct radiocarbon dates (Supplementary Table 20). To differentiate between calibrated radiocarbon ranges and molecular dates, we use cal bp before present for the former and years before present for the latter.

### Reporting summary

Further information on research design is available in the Nature Portfolio Reporting Summary linked to this article.

## Online content

Any methods, additional references, Nature Portfolio reporting summaries, source data, extended data, supplementary information, acknowledgements, peer review information; details of author contributions and competing interests; and statements of data and code availability are available at 10.1038/s41586-023-06923-7.

### Supplementary information

Supplementary Information Supplementary Information, including Supplementary Figs. 1–27, Tables 1–12 and 14–22, legends for Tables 13 and 23–26, and References.

Reporting Summary

Supplementary Table 13 Sample pretreatment information and radiocarbon dates from material excavated between 2016 and 2022 from layers 11–7 at Ranis.

Supplementary Table 23 Proteomic taxonomic identification of all bone specimens recovered in Ranis during the 2016–2022 excavation.

Supplementary Table 24 Proteomic taxonomic identification of all bone specimens recovered in Ranis during the 1932–1938 excavation.

Supplementary Table 25 Summary of sequencing data of libraries evaluated for human mtDNA content.

Supplementary Table 26 Provenance information and radiocarbon dating of human remains from Ranis.

## Figures and Tables

**Extended Data Table 1:**
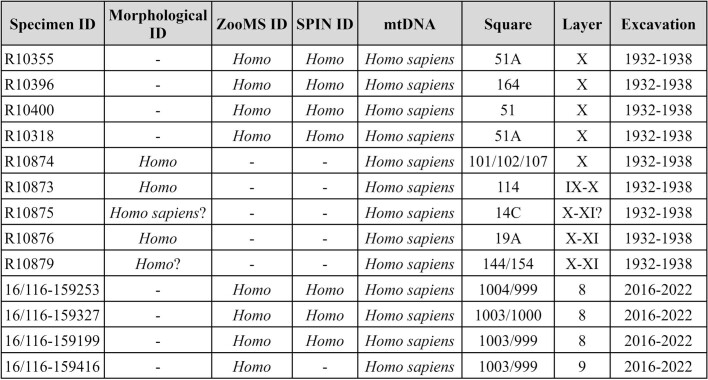
Hominin specimens identified in Ranis

Hominin specimens are organised by their recovery during either the 1932–1938 or 2016–2022 excavations.

## Data Availability

The raw LC–MS/MS proteomics data for the DIA search have been deposited to the ProteomeXchange Consortium through the PRIDE^77^ partner repository under accession code PXD043272. The raw LC–MS/MS and MaxQuant search proteomics data for six bone specimens analysed in DDA mode included in this study have been deposited to the ProteomeXchange Consortium through the PRIDE partner repository with the dataset identifier PXD042321. The MALDI–TOF.mzml and.msd type files included in this study are available at 10.5281/zenodo.8063812 (ref. ^78^). The newly reconstructed mtDNA sequences are available at 10.5061/dryad.1jwstqk0s and the sequencing data are available at the European Nucleotide Archive (PRJEB67776).

## Code availability

The full code description is provided in the Supplementary Information.
