# Supplementary material for: Homo sapiens reached the higher latitudes of Europe by 45,000 years ago
Source: Nature. 2024 Jan 31;626(7998):341–6. doi: 10.1038/s41586-023-06923-7 (PMC10849966; doi:10.1038/s41586-023-06923-7)
Supplement: Supplementary file 1 — Supplementary Information, including Supplementary Figs. 1–27, Tables 1–12 and 14–22, legends for Tables 13 and 23–26, and References. [file 41586_2023_6923_MOESM1_ESM.pdf]

nature portfolio

https://doi.org/10.1038/s41586-023-06923-7

Supplementary information

Homo sapiens reached the higher latitudes of Europe by 45,000 years ago

In the format provided by the authors and unedited

Homo sapiens reached the higher latitudes of Europe by 45,000 years ago

Authors

Dorothea Mylopotamitaki[1,2]*, Marcel Weiss[3,2]*#, Helen Fewlass[4,2]*, Elena Irene Zavala[5,2]*, Hélène Rougier[6], Arev Pelin Sümer[2], Mateja Hajdinjak[2], Geoff M. Smith[7,2], Karen Ruebens[1,2], Virginie Sinet-Mathiot[8,2], Sarah Pederzani[9,2], Elena Essel[2], Florian S. Harking[10], Huan Xia[11,] Jakob Hansen[12,13], André Kirchner[14], Tobias Lauer[15,2], Mareike Stahlschmidt[16,2], Michael Hein[17,18 2], Sahra Talamo[19,2], Lukas Wacker[20], Harald Meller[21], Holger Dietl[21], Jörg Orschiedt[21], Jesper V. Olsen[10], Hugo Zeberg[2,22], Kay Prüfer[2], Johannes Krause[2], Matthias Meyer[2], Frido Welker[12], Shannon P. McPherron[2], Tim Schüler[23], Jean-Jacques Hublin[1,2]#

* Shared first author

\# Corresponding authors. Marcel Weiss: marcel.weiss@fau.de; Jean-Jacques Hublin: hublin@eva.mpg.de.

Affiliations

1 Chair of Paleoanthropology, CIRB (UMR 7241–U1050), Collège de France, Paris, France.

2 Max Planck Institute for Evolutionary Anthropology, Leipzig, Germany.

3 Friedrich-Alexander-Universität Erlangen-Nürnberg, Institut für Ur- und Frühgeschichte, Erlangen, Germany.

4 Ancient Genomics Lab, Francis Crick Institute, London, United Kingdom.

5 Department of Molecular and Cellular Biology, University of California Berkeley, Berkeley, USA.

6 Department of Anthropology, California State University Northridge, Northridge, CA, USA.

7 School of Anthropology and Conservation, University of Kent, Centerbury, United Kingdom.

8 Univ. Bordeaux, CNRS, Ministère de la Culture, PACEA, UMR 5199, Bordeaux, France

9 Archaeological Micromorphology and Biomarker Lab, University of La Laguna, San Cristóbal de La Laguna, Tenerife, Spain.

10 Center for Protein Research, University of Copenhagen, Copenhagen, Denmark.

11 College of Earth and Environmental Sciences, Lanzhou University, Lanzhou, China.

12 Globe Institute, University of Copenhagen, Copenhagen, Denmark.

13 Departament de Prehistòria, Universitat Autònoma de Barcelona, Barcelona, Spain.

14 Department of Soil Protection and Soil Survey, State Authority for Mining, Energy and Geology of Lower Saxony (LBEG), Hannover, Germany.

15 Terrestrial Sedimentology, Department of Geosciences, University of Tübingen, Tübingen, Germany.

16 Department of Evolutionary Anthropology and Human Evolution and Archaeological Sciences (HEAS), University of Vienna, Vienna, Austria.

17 Institute of Ecology, Leuphana University, Lüneburg, Germany.

18 Historical Anthropospheres Working Group, Leipzig Lab, Leipzig University, Leipzig, Germany.
19 Department of Chemistry G. Ciamician, Bologna University, Bologna, Italy.
20 Ion Beam Physics, ETH Zurich, Zurich, Switzerland.
21 Landesamt für Denkmalpflege und Archäologie Sachsen-Anhalt- Landesmuseum für Vorgeschichte, Halle, Germany.
22 Department of Physiology and Pharmacology, Karolinska Institutet, Stockholm, Sweden.
23 Thuringian State Office for the Preservation of Historical Monuments and Archaeology, Weimar, Germany.

Contents

1. Background, fieldwork, and analysis of the cave site Ilsenhöhle in Ranis 7
1.1. The Lincombian-Ranisian-Jerzmanowician 7
Figure S1. Ranis, the LRJ and other MP/UP transitional technocomplexes, Brno Bohunice, Bacho Kiro, and Zlatý kůň. 9
1.2. Site description, fieldwork, and artefacts 10
Table S1. Artefacts from the 1932-1938 collection. 11
Figure S2. The site Ilsenhöhle in Ranis. 16
Figure S3. Plan of the excavation 1932-1938 (Hülle 1977)15 with the location of the 2016-2022 excavation. 17
Figure S4. Plan of the 2016-2022 excavation. 18
Figure S5. Border of the 1934 trench with alternating backfill (right) backed against in situ sediments to the left. 19
Figure S6. Situation at the end of season 2019. 20
Figure S7. Excavating the LRJ layers after the large rock was removed. 21
Figure S8. Excavating the LRJ layers after the large rock (purple) was removed. 22
Table S2. Excavated sediment. 23
Table S3. Artefacts layers 7 to 11. 24
1.3. Micromorphology of layers 7 to 9 27
Figure S9. Micromorphological analysis of thin section RNS 20 4 containing layers 7 Brown, 8 and 9. 27
1.4. Sedimentological results layer 7 to 9 29
1.4.1. Carbon and nitrogen 29
1.4.2. Grain size analysis 29
Table S4: Sedimentological results. 30
1.5. Analysis of the 1930s collection 31
Table S5. List and provenience of the human remains (R10318, R10355, R10873, R10396, R10400, R10874, R10875, R10876, R10879) identified in the old collection at Ranis along with the lithic artefacts (2, xx) found in layer X in the same squares. 32
2. Proteomic screening 34
2.1. Zooarchaeology by Mass spectrometry (ZooMS) 34
2.2. Species by Proteome INvestigation (SPIN) 34
2.3. Hominins 36
2.4. Deamidation 36
Table S6. Blank identifications for both proteomic approaches ZooMS and SPIN. 38
Table S7. List of taxonomic identifications by ZooMS and SPIN and their respective counts for the entire proteomic dataset for layers X (1932-1938 excavation), 9 and 8 (2016-2022 excavation). 39
Table S8. List of non-compatible taxonomic identifications between ZooMS and SPIN and their respective counts for layers X (1932-1938 excavation), 9 and 8

(2016-2022 excavation). 42
Figure S10. Protein degradation for the archaeological bone specimens with ZooMS and directDIA SPIN for layers X (1932-1938 excavation), 9 and 8 (2016-2022 excavation). 43
Figure S11. Pie chart of taxonomic identification obtained through ZooMS and SPIN and their compatible/non-compatible status for layers X (1932-1938 excavation), 9 and 8 (2016-2022 excavation). 45
Figure S12. Pie chart of potentially incompatible taxonomic identifications and the potential reason for the non-matching taxonomic identifications for layers X (1932-1938 excavation), 9 and 8 (2016-2022 excavation). 46
3. Zooarchaeology 47
Table S9. Analysed faunal remains from layers 9-7 at Ranis from 1932-1938 and 2016-2022 excavations. 49
Table S10. Weathering stages (from Behrensmeyer71) for bone material from 1932-1938 and 2016-2022 excavations. 52
Table S11. Bone surface readability for bone remains from 1932-1938 and 2016-2022 excavations. 52
Table S12. Faunal remains from 1932-1938 and 2016-2022 excavations with the number of specimens that record carnivore and human modification. 52
4. Radiocarbon dating 53
4.1. Bone pretreatment 53
Table S14. Comparison of AMS dates for two bones prepared with two collagen extraction protocols. 53
Figure S13. FTIR spectra of human bones R10355 and R10396. 55
4.2. Bayesian modelling 56
Table S15. Modelled boundaries of 14C dates included in the site chronological model shown in Extended Data Figure 3. 57
Table S16. Modelled boundaries of 14C dates in a site chronological model where layers 9 and 8 dates are modelled together in one LRJ phase. 59
Table S17. Radiocarbon and thermoluminescence (TL) (Brno-Bohunice 2) dates from contemporary sites and human remains included in Fig. 2. 61
4.3. OxCal code for site model shown in Extended Data Fig. 3 and Tab. S15. 64
5. Ancient DNA analysis 68
5.1. Lineage determination 68
Figure S14. The frequency of base substitutions relative to the human mtDNA reference genome for filtered DNA fragments (length > 34bp, mapping quality >= 25) recovered from R10879. 68
Figure S15. The frequency of base substitutions relative to the human mtDNA reference genome for filtered DNA fragments (length > 34bp, mapping quality >= 25) recovered from R10874. 68
Figure S16. The frequency of base substitutions relative to the human mtDNA reference genome for filtered DNA fragments (length > 34bp, mapping quality >= 25) recovered from R10876. 69
Figure S17. The frequency of base substitutions relative to the human mtDNA reference genome for filtered DNA fragments (length > 34bp, mapping quality >=

25) recovered from R10875. 69

Figure S18. The frequency of base substitutions relative to the human mtDNA reference genome for filtered DNA fragments (length > 34bp, mapping quality >= 25) recovered from 16/116-159416. 69

Figure S19. The frequency of base substitutions relative to the human mtDNA reference genome for filtered DNA fragments (length > 34bp, mapping quality >= 25) recovered from 16/116-159327. 70

Figure S20. The frequency of base substitutions relative to the human mtDNA reference genome for filtered DNA fragments (length > 34bp, mapping quality >= 25) recovered from 16/116-159253. 70

Figure S21. The frequency of base substitutions relative to the human mtDNA reference genome for filtered DNA fragments (length > 34bp, mapping quality >= 25) recovered from 16/116-159199. 70

Figure S22. The frequency of base substitutions relative to the human mtDNA reference genome for filtered DNA fragments (length > 34bp, mapping quality >= 25) recovered from R10396. 71

Figure S23: The frequency of base substitutions relative to the human mtDNA reference genome for filtered DNA fragments (length > 34bp, mapping quality >= 25) recovered from R10355. 71

Figure S24. The frequency of base substitutions relative to the human mtDNA reference genome for filtered DNA fragments (length > 34bp, mapping quality >= 25) recovered from R10873. 71

Table S18. The estimated percent support for the human lineage using previously published diagnostic positions. 72

5.2. Reconstruction of mtDNA genomes 72

Table S19. Support for consensus base calling at position 16,093 of the human mtDNA genome (rCRS) for ten of the Ranis datasets. 74

Figure S25. The pairwise differences among the ten new mtDNA genomes and the previously published mtDNA genomes from the Zlatý kůň and Fumane 2 individuals. 75

5.3. Tree building and branch shortening estimates 75

Table S20. List of previously published radiocarbon dates and mtDNA genomes for ancient modern humans used for molecular dating with BEAST. 77

Table S21. The marginal log likelihoods for different clock and tree models. 78

Table S22. The estimated tip dates of newly reconstructed mtDNA genomes and the previously published Zlatý kůň and Fumane 2 mtDNA genomes. 78

Figure S26. The estimated 95% HPD of molecular dates from BEAST2 visualised with Tracerv.1.7.1. 79

Figure S27. Tree constructed using BEAST2 of a selection of ancient and modern human mtDNA genomes. 80

6. Additional files provided as supplementary material 81

Supplementary Table S13: Sample pretreatment information and radiocarbon dates from material excavated between 2016-2022 from layers 11 - 7 at Ranis. 81

Supplementary Table S23: Proteomic taxonomic identification of all bones specimens recovered in Ranis during the 2016-2022 excavation. 81

Supplementary Table S24: Proteomic taxonomic identification of all bones

specimens recovered in Ranis during the 1932-1938 excavation. 81
Supplementary Table S25: Summary of sequencing data of libraries evaluated for human mitochondrial DNA content 81
Supplementary Table S26: Provenance information and radiocarbon dating of human remains from Ranis. 81
7. References **82**

1. Background, fieldwork, and analysis of the cave site Ilsenhöhle in Ranis

1.1. The Lincombian-Ranisian-Jerzmanowician

The Lincombian-Ranisian-Jerzmanowician (LRJ)[1–3] is a technocomplex associated with the Middle to Upper Palaeolithic (MP/UP) transition in central and northwestern Europe (for a detailed overview see Jöris et al. 2022[4]) that has previously been associated with Neanderthals[5]. Here, we refer to assemblages characterised by blade points as the main characteristic feature of the LRJ. We do so to strictly distinguish the LRJ from assemblages from the MP/UP transition period that contain no blade points but bifacial points only, such as the Altmühlian and the Szeletian. The LRJ sites spread from Poland in eastern central Europe to Britain in the northwest (Fig. S1, SI) with their southernmost occurrence in Bavaria, Germany[6]. The lithic assemblages are mostly small in number and consist mainly of semi-bifacial Jerzmanowice blade points (Fig. S2d, SI), obtained from (mostly) bidirectional volumetric cores (detailed technological description Flas 2006[3] and Demidenko & Škrdla 2023[7]). As only a handful of cores are preserved from the entire LRJ, the blank production is reconstructed based on the dorsal scar pattern of the blades and blade points[3]. A recent study has shown that the Jerzmanowice blade points potentially served as projectile points[8]. Although in a low frequency, other tools existed as well, like endscrapers, burins, retouched blades, and pointed blades[5,9].

LRJ assemblages are often found in caves, especially in carnivore occupied sites, which are mostly hyaena dens[6,9–11]. Large scale open-air sites with varying site functions were entirely missing until recently[7]. Most of the sites are found in the low mountain ranges ("Mittelgebirge") at the border of the Northern European Plain where topographic relief and geology provide a setting for the formation of caves with good archaeological preservation. Nevertheless, we can infer that the plains connecting the modern-day British Isles and central Europe were travelling routes for LRJ humans. The most important LRJ sites (Fig. S1a, SI) are Nietoperzowa Cave in the Jerzmanowice village, Poland[12], Spy cave in Belgium[13], Beedings in the UK[9,14], and the site of Ilsenhöhle in Ranis in Germany[15] (hereafter: Ranis). The latter is exceptional, as besides Jerzmanowice points, a high number of large bifacial leaf points occurred (Fig. S2c, SI; see below), which were otherwise rather less frequent in the LRJ[9].

The majority of the LRJ assemblages could be interpreted as short-term and ephemeral hunting stays. Some suggest a sympatric relationship between hyenas and humans, the latter sometimes foraging in hyena dens for meat caches[10,11].

A recent paper[7] has revealed that LRJ sites, which were used for other activities than hunting, such as blank production, are located in Moravia, particularly in the area around Brno. The most significant sites, as shown in Fig. S1a, SI, are Želešice III (~46,000 - 44,000 cal BP) and Líšeň Podolí I (~42,000 - 40,000 cal BP). The latter even yielded non-local marine shells stained with black manganese oxides and red hematite, some of which were perforated and interpreted as personal ornaments. Importantly, these LRJ sites advanced our technological knowledge of the LRJ, and the authors demonstrated that they were sharing key

typo-technological features with the Bohunician. These LRJ sites in Moravia post-dated the Bohunician and proposed a seamless technological shift between these two technocomplexes[7], with the major shift related to point production. This shift involved transitioning from Levallois points produced on bi-directional cores to Jerzmanowice points made on large blades from bi-directional volumetric cores. However, other aspects such as bi-directional blank production, faceting to adjust the striking platform instead of core tablets, the central crested blade to initialise the core, and thick endscrapers were retained. With its proposed relation to the Bohunician, the authors demonstrated that the LRJ was part of the IUP in Europe.

Geographically and chronologically overlapping with the Bohunician in Moravia[16] (Fig. S1b, SI), but extending further to the east, is the Szeletian, rich in bifacial leaf points[17,18]. To the west of the Bohunician, in southern Germany, we find comparable bifacial leaf points in the so-called MP/UP transitional Altmühlian[19,20] (Fig. S1b, SI), a technocomplex of the MP/UP transitional period that is usually interpreted as having evolved out of Late Middle Palaeolithic bifacial contexts[4]. The high frequency of large bifacial leaf points at Ranis and their typo-technological and raw material relatedness to the Altmühlian[21] could be interpreted in favour of close contacts to the makers of bifacial leaf point assemblages. The absence of any Neanderthal DNA at Ranis would, however, make it unlikely that these contacts would relate to Neanderthals. Following this line of argument we could infer that at least some of the leaf point assemblages that contain no blade points also might have been made by *H. sapiens*. Future research will have to address if Altmühlian and Szeletian assemblages may further sketch the way of *H. sapiens* into Europe.

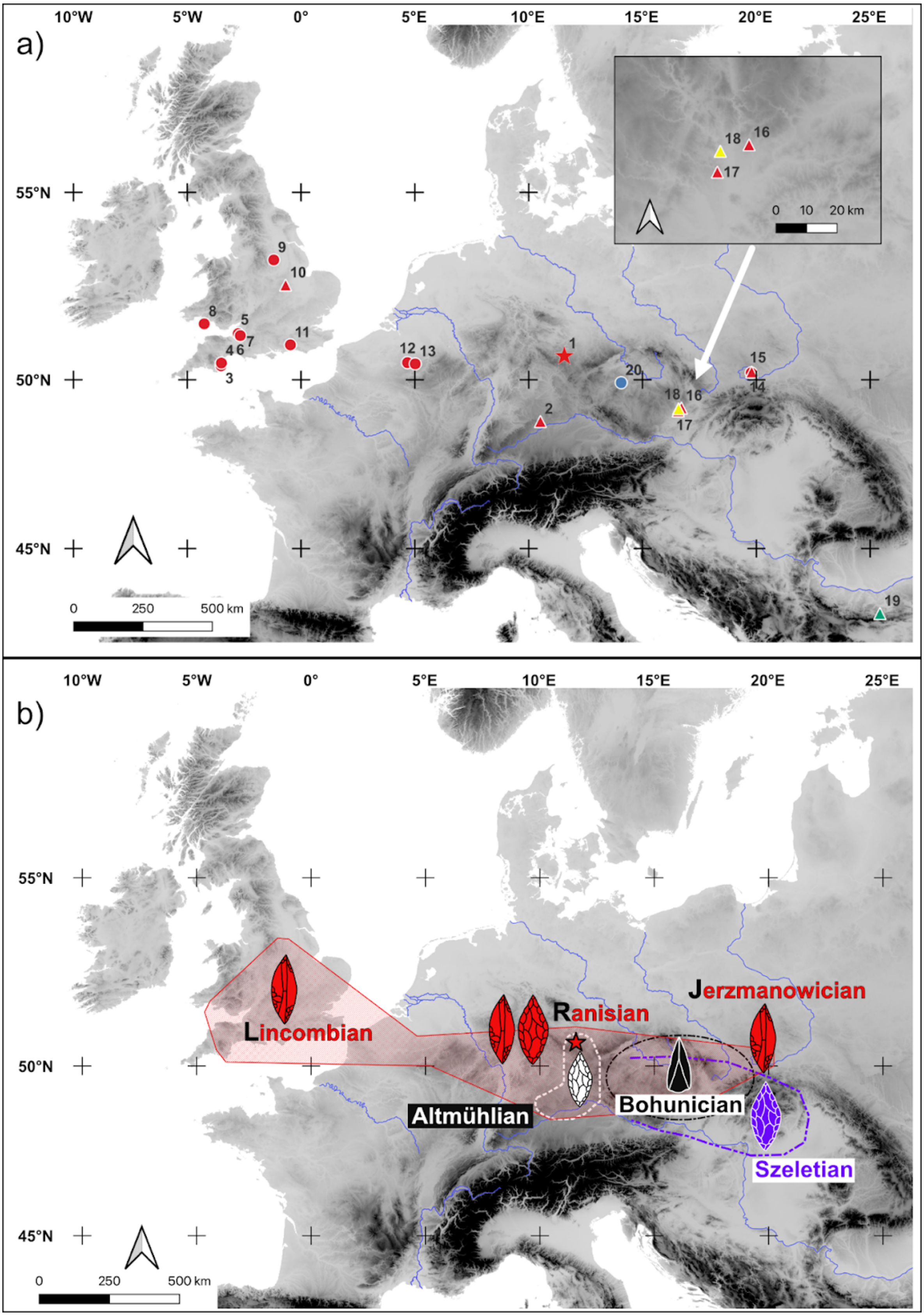

Figure S1. Ranis, the LRJ and other MP/UP transitional technocomplexes, Brno Bohunice, Bacho Kiro, and Zlatý kůň.

a) Location of main LRJ sites (1-7 & 9-15: adopted from Hussain et al. 2022[11], 8: Aldhouse-Green 2000[22], 16-17: Demidenko & Škrdla 2023[7], 18: Škrdla 2017b[23], 19: Hublin et

al. 2020[24], 20: Prüfer et al. 2021[25]): 1 – Ranis, 2 – Schmähingen-Kirchberghöhle, 3 – Bench Quarry, 4 – Kent's Cavern, 5 – Soldier's Hole, 6 – Hyena Den, 7 – Badger Hole, 8 – Paviland Cave, 9 – Robin Hood's Cave, 10 – Grange Farm, 11 – Beedings, 12 – Spy, 13 – Goyet, 14 – Nietoperzowa Cave, 15 – Koziarnia Cave, 16 – Líšeň Podolí I, 17 – Želešice III, 18 - Brno Bohunice, 19 - Bacho Kiro, 20 - Zlatý kůň. Triangles mark dated sites shown in Fig. 2, main text. **b**) Location of Ranis (star) and the main techno-complexes with symbols schematically representing the different point types. Red: LRJ with blade (Jerzmanowice) and bifacial points, White: Altmühlian (Blattspitzengruppe) with bifacial points, Black: Bohunician with elongated Levallois points, Purple: Szeletian with bifacial points. The maps were created with QGIS[26] based on SRTM data V4 (http://srtm.csi. cgiar.org)[27]. b) was edited with Affinity Designer[28].

1.2. Site description, fieldwork, and artefacts

The site Ilsenhöhle at Ranis (50°39.7563'N, 11°33.9139'E) is located in the Orla River valley in Thuringia, Germany, 30 km south of the city of Jena. The cave formed in the south-facing cliff of a Permian limestone reef (Fig. S2a, SI). Only two short chambers remained intact from a formerly large and high chamber that collapsed during the Late Pleistocene[15]. Initial fieldwork was conducted by the then-owner of the Ranis castle (Burg Ranis), Dietrich von Breitenbuch, in the years 1926, 1929, 1931, and 1932. After he discovered the first Pleistocene layers, Werner M. Hülle from the former "Landesanstalt für Vorgeschichte in Halle" took over and excavated the site between 1932-1938[15]. Of special interest for the current study is layer X or the so-called "Graue Schicht" ("Grey layer") found near the base of the sequence, where Hülle discovered 63 tools (no debitage). These consist of 23 bifacial leaf points (Fig. S2c, SI) and 17 Jerzmanowice blade points (Fig. S2d, SI), including their fragments (Tab. S1, SI). The other 24 tools are pointed blades, scrapers, and other retouched pieces. The composition of blade and bifacial leaf points in such high quantity is unique and made Ranis the eponymous site for the Ranisian as part of the Lincombian-Ranisian-Jerzmanowician technocomplex of the MP/UP transition period[1,2].

Table S1. Artefacts from the 1932-1938 collection.

The ID refers to the IDs / Figure numbers given in Hülle 1977[15]. Note that only IDs 45, 75, and 76 are not illustrated in Hülle 1977[15].

ID	Raw Material	Classification	Preservation	Notes
2, 18	flint	Jerzmanowice point	complete	
2, 19	flint	Jerzmanowice point	complete	pointed blade?
2, 20	flint	Jerzmanowice point	complete	made of a flake?
2, 21	flint	bifacial point	complete	from blade: ventral completely retouched
2, 22	flint	Jerzmanowice point	complete	
2, 23	flint	Jerzmanowice point	complete	
2, 24	flint	bifacial point	complete	from blade: ventral completely retouched
2, 25	flint	Jerzmanowice point	complete	
2, 26	flint	pointed blade/ Jerzmanowice point	medial-distal	
2, 27	flint	pointed blade	complete	
2, 28	flint	pointed blade	complete	
2, 29	flint	pointed blade	complete	
2, 30	flint	Jerzmanowice point	medial-distal	
2, 31	flint	pointed blade	complete	
2, 32	flint	Jerzmanowice point	distal	
2, 33	flint	Jerzmanowice point	distal	
2, 34	flint	retouched flake/ blade	complete	
2, 35	flint	bifacial point	distal	
2, 36	flint	Jerzmanowice point	distal	
2, 37	flint	Jerzmanowice point	proximal	
2, 38	flint	bifacial point	distal	
2, 39	flint	Jerzmanowice point	distal	
2, 40	flint	bifacial point	distal	
2, 41	flint	bifacial point	distal	
2 42	flint	bifacial point	distal	
2, 43	flint	Jerzmanowice point	medial-distal/ proximal	
2, 44	flint	bifacial point	distal	
2, 45	flint	Jerzmanowice point	distal or proximal	
2, 46	flint	bifacial point	complete	asymmetric
2, 47	flint	bifacial point	medial-distal	
2, 48	flint	bifacial point	medial-distal	asymmetric
2, 49	flint	bifacial point	complete	Type Moravany Váhom-Dlhá (Szeletian)
2, 50	flint	bifacial point	complete	Type Moravany Váhom-Dlhá (Szeletian)

2, 51	flint	bifacial point	complete	basal tip broken
2, 52	flint	bifacial point	complete	asymmetric
2, 53	flint	bifacial point	complete	
2, 54	flint	bifacial point	complete	basal tip broken
2, 55	flint	bifacial point	complete	
2, 56	flint	bifacial point	complete	
2, 57	flint	bifacial point	complete	
2, 58	flint	bifacial point	complete	
2, 59	flint	Jerzmanowice point	medial	
2, 60	flint	Jerzmanowice point	complete	
2, 61	flint	bifacial scraper	medial-distal	
2, 62	flint	bifacial scraper (leaf-shaped)	complete	
2, 63	flint	bifacial scraper (leaf-shaped)	complete	
2, 64	flint	unifacially shaped scraper (Limace)	complete	
2, 65	flint	point	complete	
2, 66	flint	thick pointed blade	complete	facetted platform
2, 67	flint	retouched blade	medial	
2, 68	flint	retouched pointed blade	proximal-medial	
2, 69	flint	bifacially retouched blade	proximal	fragment of point?
2, 70	flint	retouched pointed blade	distal	
2, 71	flint	retouched blade	medial-distal	
2, 72	flint	bifacial scraper (Keilmesser?)	complete	
2, 73	flint	unifacially shaped tool	medial	fragment of leaf point?
2, 74	flint	Jerzmanowice point	medial	
2, 75	flint	scraper	complete	
2, 76	flint	bifacial point	fragment	
2, 77	Jurassic chert	bifacial point	medial	
2, 78	quartzite	blade	medial	
2, 79	quartzite	bifacial tool	medial	fragment of leaf point?
2, 80	quartzite	chunk	medial	fragment of thick blade?

Ranis lies at the northern boundary of the German low mountain ranges to the Leipzig embayment, a southern extension of the northern European Plain into which glaciers advanced furthest south during the Elsterian glaciation, depositing the Baltic flint which has been most frequently used at Ranis. The Elsterian ice advance reached as close as ~30 km to the north of Ranis. Whereas Jerzmanowice blade points are mainly spread from Poland to Britain[2,5,9], bifacial leaf points occur in the southern part of Germany, in the so-called Altmühlian or Blattspitzengruppe[19,29–31]. Based on the evidence from Ranis, the assemblage looks like a mix of more southern and northwestern and central European elements of transitional period technocomplexes. This is further supported by the raw materials. Whereas most tools are made of large-sized Baltic Flint, found in the lowlands north of Ranis, some quartzite artefacts point to the central and southern regions of the low mountain ranges. One fragment of a bifacial leaf point (Plate 36: 2,77)[15] was made of Jurassic chert, potentially originating from the Bavarian Altmühl Valley[21]. This is the same raw material that the bifacial leaf points of the Altmühlian, specifically from the site of Mauern[32], are made of.

The presence of blade points and bifacial leaf points, as well as the position between northern and southern regions, made Ranis a key site for understanding the MP/UP transition in central and northwestern Europe. Therefore, our team from the Thüringer Landesamt für Denkmalpflege und Archäologie and the Department of Human Evolution at the Max Planck Institute for Evolutionary Anthropology returned to Ranis in 2016 to re-open and re-excavate the site (Fig. S2, SI). Our goals were the clarification of the stratigraphy, the dating of the sequence, as well as the identification of the makers of the LRJ at Ranis. The fieldwork lasted until 2022.

We decided to re-open the 1934 East-West trench (Fig. S3, SI). Based on the published excavation plans, we expected a larger unexcavated area directly south of it. Our aim was to open the trench and to excavate adjacent squares of the southern profile to bedrock (Fig. S4, SI). Due to the presence of large rocks from the collapsed cave roof, our excavation area progressively reduced as we proceeded into the deeper stratigraphy (Fig. S4, SI), finally reaching a depth of 8 m (Fig. S8, SI). In the end we were able to excavate a surface of 2¼ square metres (one complete square, two half squares, and one quarter of a square of the excavation grid).

In 2016, we started with a 3x4 m area close to the cave wall (Figs. S2b and S3, SI). We detected the old trench as well as in-situ sediments at the end of the first week (Fig. S5, SI). During the campaigns in 2017 and 2018, we widened the trench and went down through the upper layers. Thereby, we secured the trench walls with wooden planks and metal poles, done by a professional construction firm. All the layers excavated so far (1 to 5, see Fig. 1, main text) were deposited after the collapse of the cave roof, i.e., on top of the fallen cave roof in an open-air environment.

In 2019, our team reached the first cave deposit, layer 6, containing UP artefacts. However, at the end of the season and a few decimetres into layer 6, we found a large rock extending across our entire trench bottom (Fig. S6, SI), including parts of the 1934 trench. Having the expectation that the basal sequence including the LRJ layer would be still preserved below the rock, we removed the rock in the winter of 2019/2020. Our hypothesis held true, and we additionally gained one and a half squares of intact sediment below the rock from the 1934 trench, as Hülle did not remove parts of the rock and did not excavate below it.

Additionally, the rock fortuitously sealed the LRJ from the overlying UP of layer 6 and prevented post-depositional disturbances by animals (cave bears, hyenas).

Finally, in 2020 and 2021, we excavated the basal sequence of the site, including the LRJ (Figs. S7 & S8, SI). In the following we focus on layers 12 to 7. layers 14 and 13 were two thin (about 10 cm each) layers of imbricated limestone detritus overlying the bedrock without any archaeology. layer 12 was a silty-sandy lens in layer 11 and contained many stones. It had no anthropogenic input, except for 1 bone with human modifications[33]. layer 11 was a slightly humic, clayey loam with a thickness of 70 cm, which contained by far the highest number of stones and rocks in the basal Ranis sequence. In addition to 6 bones with anthropogenic modifications and 10 burnt bones[33], the layer contained 22 artefacts in the upper 30 cm. All these artefacts were undiagnostic and made of non-flint raw material, like Greywacke and crystalline quartz. Most of them were chips <1 cm, but 6 pieces were larger than 1 cm. Especially in terms of raw material, the assemblage of layer 11 matched the assemblage found in Hülle's[15] layer XI. This layer contained only 16 artefacts across the entire cave and was interpreted as Middle Paleolithic[15]. However, among these 16 artefacts, there was one thick blade, a proximal blade fragment, a bifacial pointed scraper (all made of flint), and a chert leaf point, all of which were in fact undiagnostic in this context. Due to the low number of artefacts and given that the mentioned artefacts would not be unusual in the LRJ, we are currently not sure how to interpret layer 11/layer XI. Further analysis (sedimentology, artefact analysis) are still ongoing to resolve this aspect.

Layer 10 was a silty sand which had a maximum thickness of about 20 cm in the south of our trench and became thinner (1 to 8 cm) towards the northern trench border (Extended Data Fig. 1, main text). Except for one quartzite chip <1 cm and a possible hammerstone, there were no anthropogenic inputs into layer 10. As the layer was mostly very thin, it could not be ruled out that the hammerstone and the chip were moved post-depositionally from either layers 9/8 above or layer 11 below. A layer similar to layer 10 was not reported from the 1932-1938 excavation[15].

Layer 9 was a humic silty loam with a thickness of mostly 2 to 5 cm, but with a maximum of 12 cm in some places. This layer contained only 2 small chips <1 cm. Interestingly, one was made of rock crystal. We found 1 rock crystal flake in the 1932-1934 collection, which potentially came from layer X, indicating a connection between the 1932-1938 and the 2016-2022 assemblages. Additionally, layer 9 contained three bones with anthropogenic modifications, and one burnt bone[33]. Based on archaeology and on the overlapping radiocarbon dates with layer 8 on faunal and human remains, we infer that layers 9 and 8 form one archaeological horizon.

Layer 8 was a slightly humic, silty loam that was rather thin on average (about 5 to 12 cm, with a minimum thickness of only 1 cm in the east of the trench), and less stony than layers 11 and 7 (Tab. S2, SI). This layer contained anthropogenic inputs, including charred plant material (see micromorphology section below). We recovered 9 bones with anthropogenic modifications, as well as 1 burnt bone[33]. In layer 8 we found 33 chips <1 cm and 6 artefacts between 1 and 2 cm. This layer has the highest lithic density (Supplementary Tab. S2). Twenty-eight of the chips came from 133 L of excavated sediment. Only 34.5 L of sediment were void of artefacts. The majority of the raw material was Baltic flint (Tab. S3, SI), followed by three quartzite chips, and one piece made of quartz. This matched the raw

material composition of the layer X LRJ assemblage[15]. Two of the larger flint artefacts were fragments of blades (Fig. 1, main text), connecting our assemblage to the LRJ blade component. We identified layer 8 as the main occupation of the LRJ and correlated it with layer X of the 1932-1938 excavation. layer 9 was also part of the LRJ archaeological horizon (see above), when the LRJ occupation of the cave potentially started, and which then intensified in layer 8. This correlation is based on the above described archaeological evidence, together with the presence of modified bones and charred plant remains (see Micromorphology section below), the stratigraphic position of layer 8 below the UP (and separated from it by a large roof collapse), as well as the overlapping radiocarbon dates of the human remains from the 1932-1938 collection and from layers 9 and 8. Most importantly, one human bone from layer 8 (16/116-159327, see Fig. 1, main text) is attributed to the same or maternally related individuals as four skeletal fragments from layer X of the 1932-1938 collection.

Layer 7 was a loamy silt that contained a high number of stones (≥ 10 cm) and rocks (≥ 20 cm; Tab. S2, SI). The layer had a thickness of 1 to 35 cm. In some limited places in the two northern squares of our trench, layer 7 had a thin (1-3 cm) brown humic loamy silty band that directly covered layer 8 (Extended Data Fig. 1, main text). This band was named 7 "Brown". In layer 7, we found 2 bones with anthropogenic modifications and 2 burnt bones[33]. A single artificial chunk, made of an unclear non-flint raw material (Tab. S3, SI), was found in layer 7. Three chips come from 7 Brown, and 7 originate from sediment excavated from the layer boundary of 8 and 7 (Brown) – here the exact layer attribution remains unclear. All these artefacts came from 66 L of sediment volume, and the remaining 558.5 L sediment volume of layer 7 and its boundary to layer 8 was void of artefacts. Based on this evidence, and as these artefacts were all small (<2 mm and 5 mm), we attributed these 10 chips to the archaeological horizon in layers 9 and 8. The layer 7 artefacts from the contact with layer 8 likely represented the final stages of the LRJ. We could not correlate layer 7 with layer IX or "Mittlere Braune Schicht"[15] of the 1932-1938 excavation. Our layer 7 was not a brown cave loam[15] and the large rock between layers 7 and 6 likely prevented the formation of layer IX in the position of our trench.

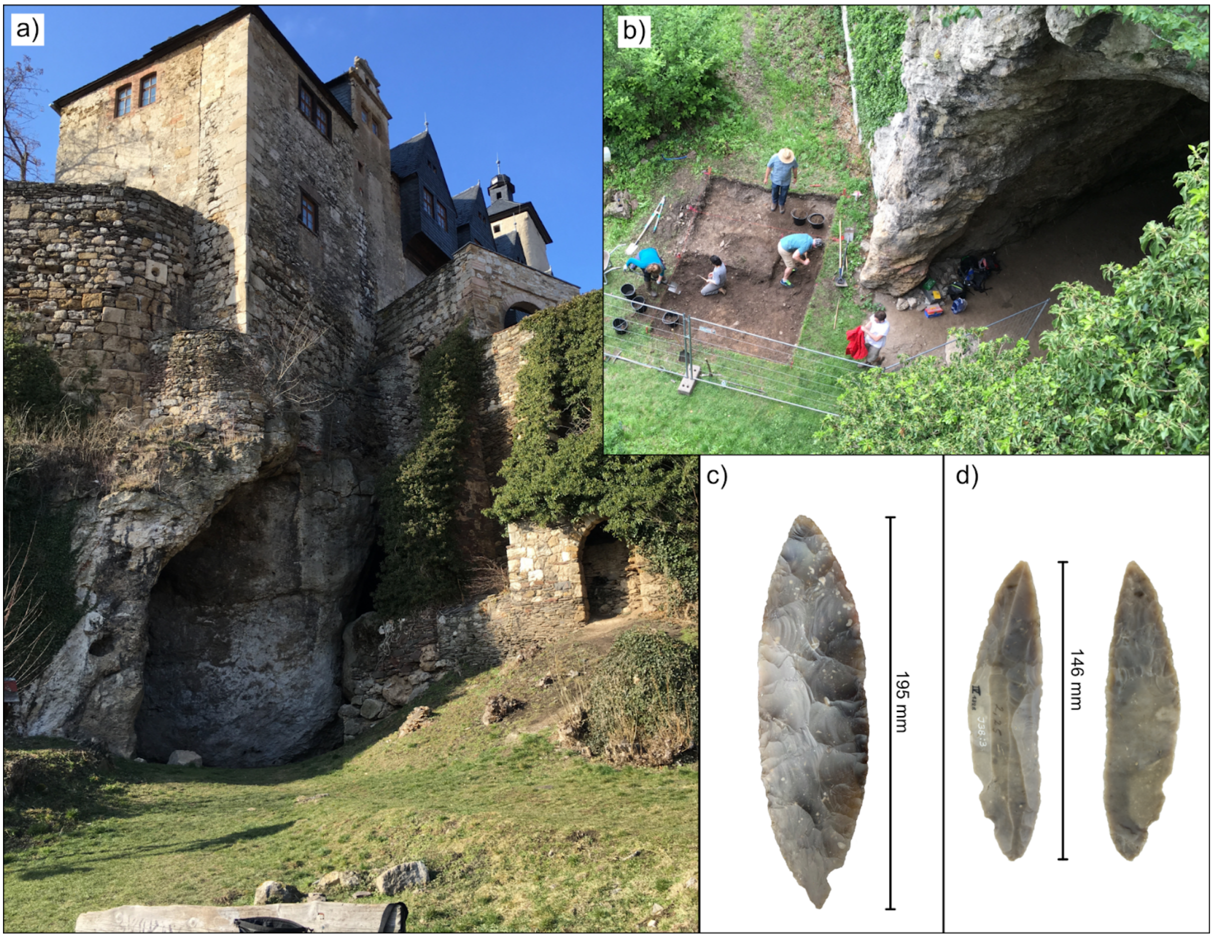

Figure S2. The site Ilsenhöhle in Ranis.

a) Ranis shortly before our excavations began. **b)** The start of the fieldwork in 2016. The entrance to the cave in b corresponds to the left edge of the cave entrance shown in a. a) and b) Photos: M. Weiss. **c)** Bifacial point from the 1932-1934 excavation (Museum Burg Ranis, IV 1319), layer X (Hülle 1977[15]: 2,56). **d)** Jerzmanowice blade point from the 1932-1934 excavation (Museum Burg Ranis, IV 1328), layer X (Hülle 1977[15]: 2, 25). c) and d) are stored in Museum Burg Ranis, photos: J. Schubert.

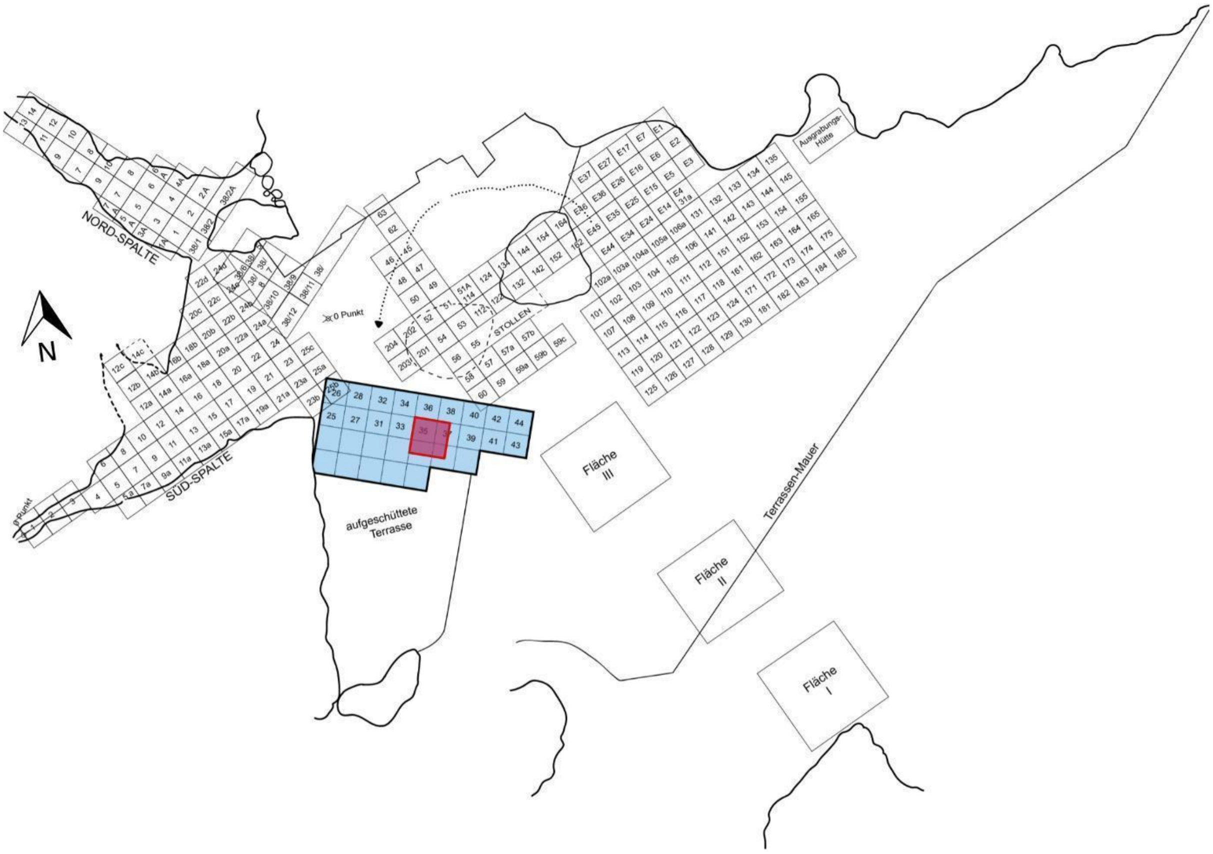

Figure S3. Plan of the excavation 1932-1938 (Hülle 1977)[15] with the location of the 2016-2022 excavation.

Each numbered square is 1 m^2. The red box marks the area that we excavated to the base of the sequence. The squares of the E-W trench in the blue box were re-numbered based on an original drawing filed in the archive of the Landesamt für Denkmalpflege und Archäologie – Landesmuseum für Vorgeschichte Halle (Saale).

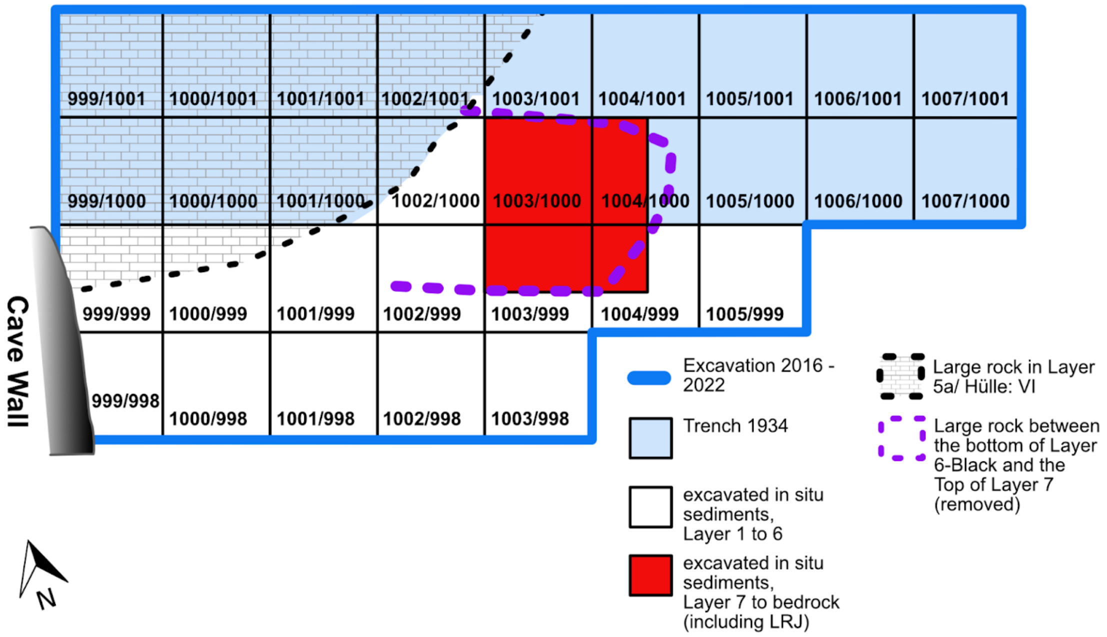

Figure S4. Plan of the 2016-2022 excavation.
Each numbered square is 1 m^2. The basal sequence including the LRJ layers were excavated in the red area of squares 1003/999, 1003/1000, 1004/999, and 1004/1000.

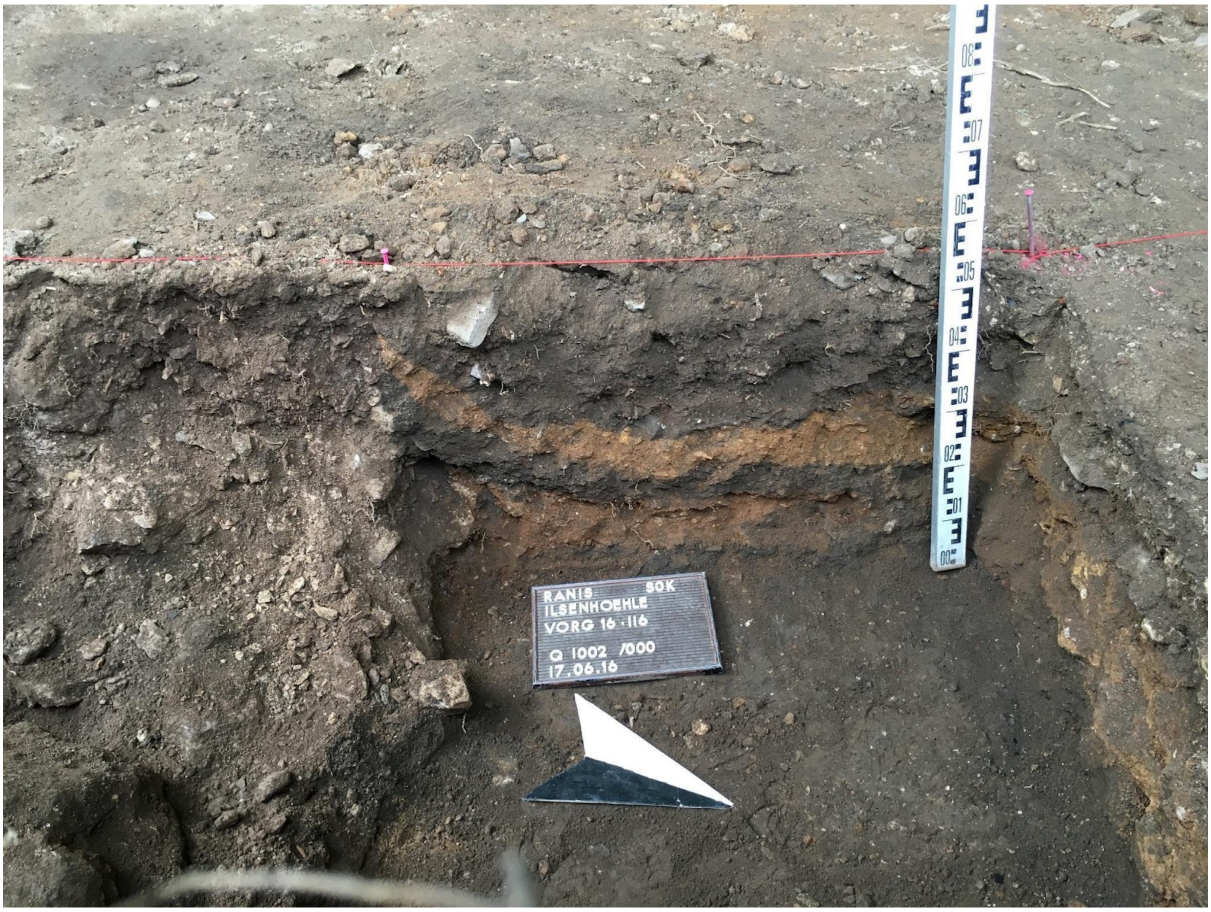

Figure S5. Border of the 1934 trench with alternating backfill (right) backed against in situ sediments to the left.
Photo: M. Weiss.

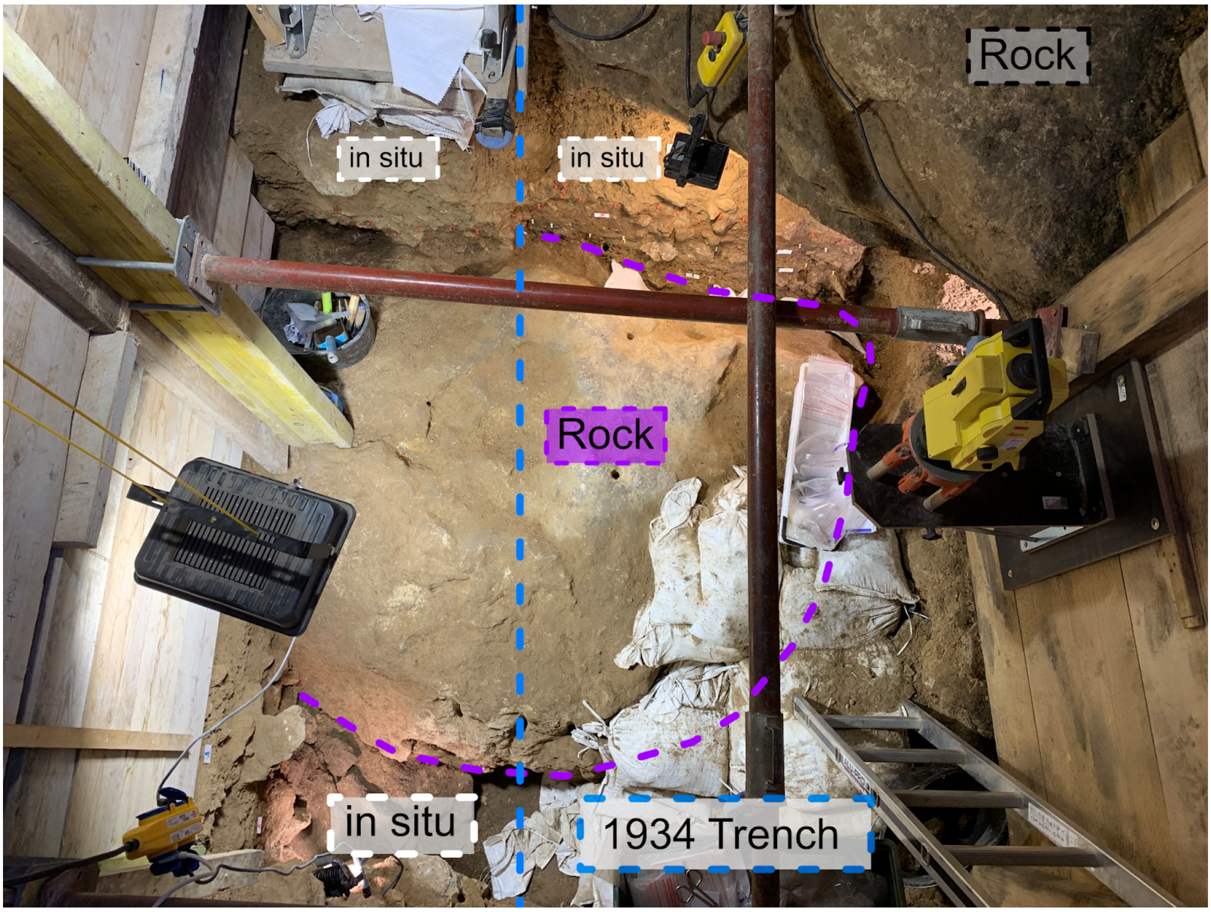

Figure S6. Situation at the end of season 2019.
Rock (grey) is part of the main roof fall within layer 5 (Hülle VI), and parts of the sediment below it were not excavated by Hülle[15]. Rock (purple; thickness 1.7 m) is sealing the entire bottom of our excavation, extending into the 1934 trench. Due to its size, this rock had not been removed by Hülle. Photo: M. Weiss.

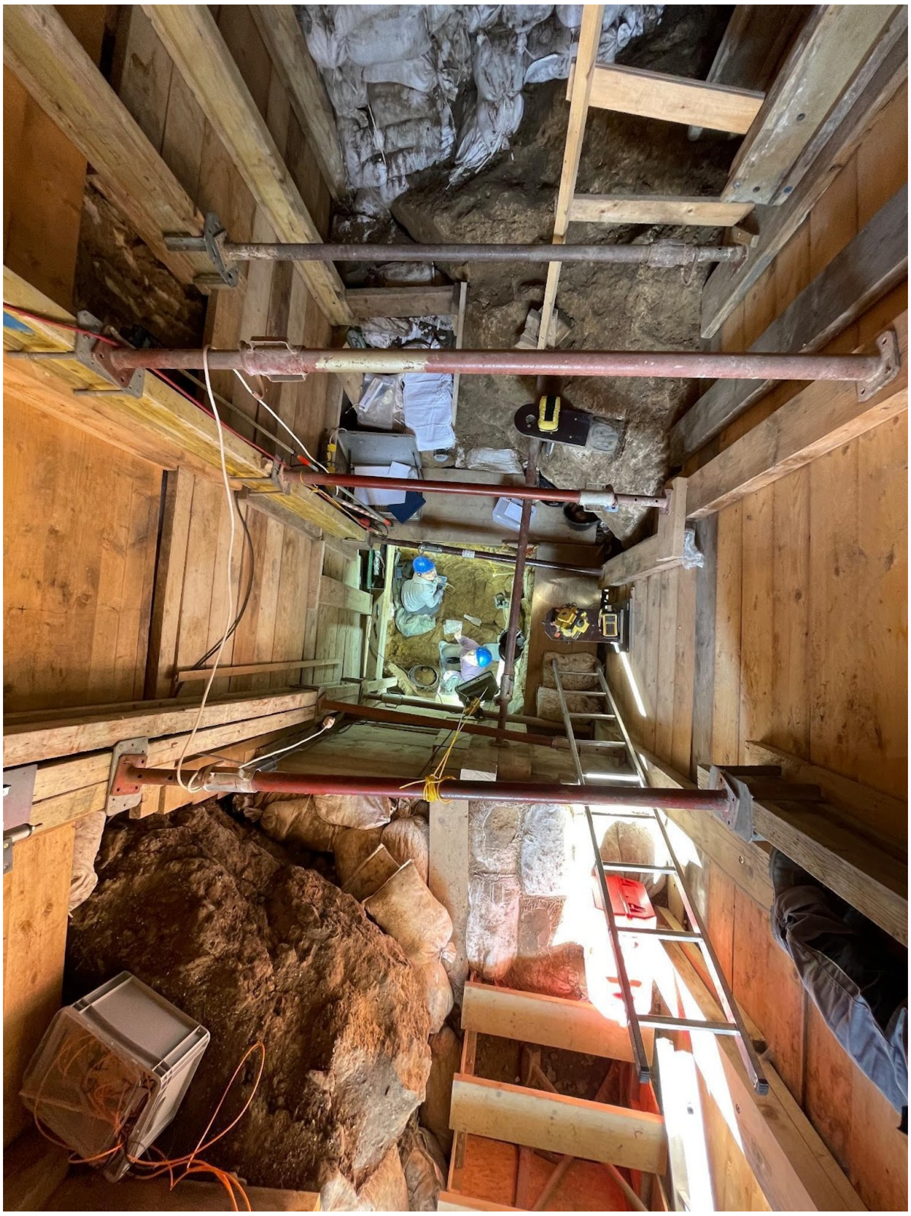

Figure S7. Excavating the LRJ layers after the large rock was removed.
Photo: M. Weiss.

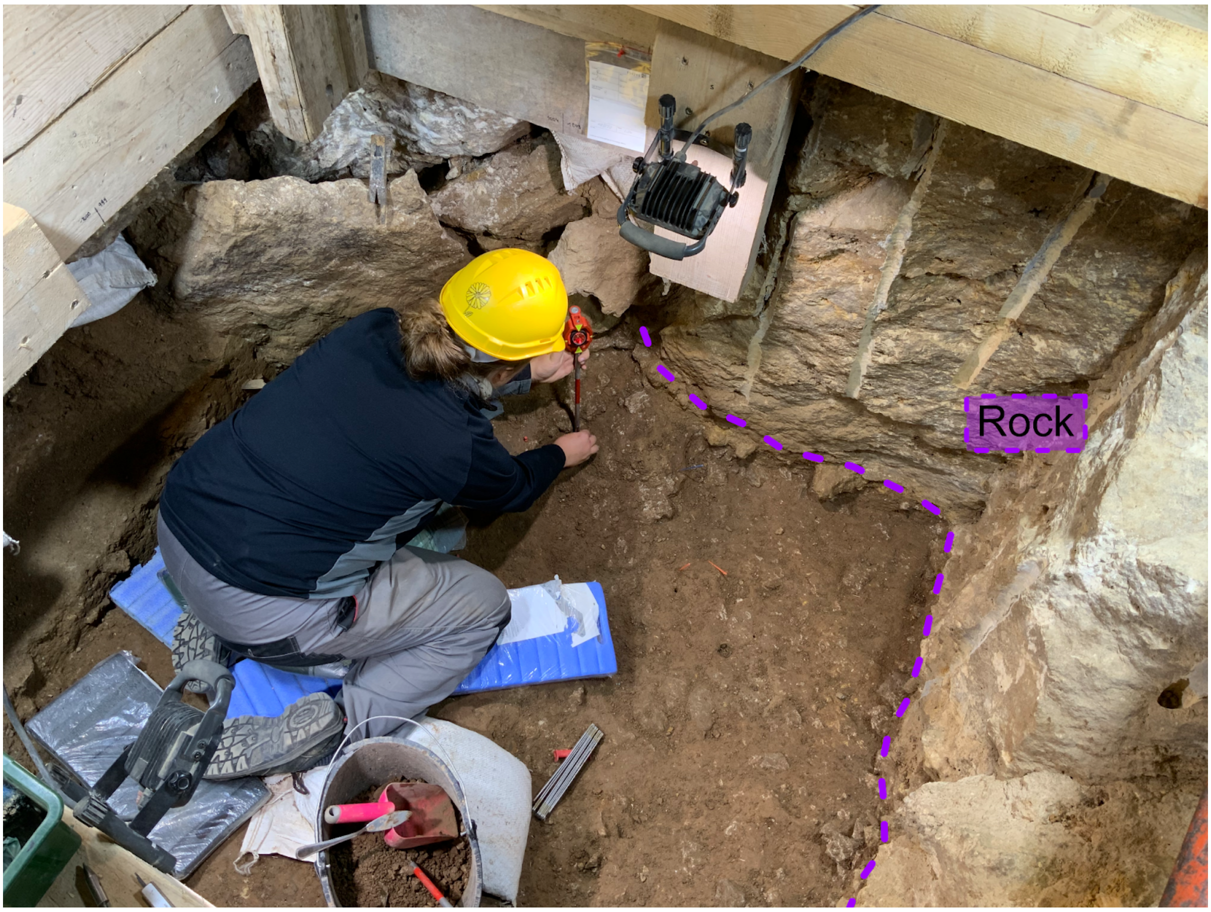

Figure S8. Excavating the LRJ layers after the large rock (purple) was removed.
Photo: M. Weiss.

Table S2. Excavated sediment.

Layer	Sediment description (Munsell Color)	Excavated sediment in litres	Stones ≥ 10 cm	Rocks ≥ 20 cm	Lithic density
**7**	(humic) loamy silt (10YR 5/4 to 7.5YR 4/3)	568.5	87	15	0.002
**Boundary 7/8**	-	56	1	0	0.2 (Layer 7 + Boundary 7/8 = 0.02)
**8**	humic loamy silt (5YR 5/3 to 7.5YR 3/3)	167.5	30	6	0.23
**9**	humic silty loam (5YR 4/4 to 7.5YR 4/4)	151	12	0	0.01
**10**	silty sand (2.5Y 5/6)	150.5	24	0	0.01
**11**	(humic) clayey loam (10YR 4/4 to 5YR 3/3)	1077	169	24	0.02
**12**	silty sand (10YR 5/6 to 2YR 5/6)	44	20	0	0

Table S3. Artefacts layers 7 to 11.

*except one, these lithics come from the screening fraction of sediment buckets from the contact of layers 7 and 8, or 7-Brown, a thin, spatially limited subcontext that was directly overlying ayer 8. It appears most likely they were displaced from layer 8.

Artefact ID	Layer	Subcontext	Count	Raw material	Size class	Classification
**Layer 7**						
16/116-149957	7/8*	Brown	1	Flint	screen (4 mm)	chip
16/116-150406	7		1	N.A. (not Flint)	1-2 cm	chunk
16/116-150224	7*	Brown	1	Flint	screen (2 mm)	chip
16/116-150307	7*	Brown	1	Flint	screen (4 mm)	chip
16/116-150352	7/8*		2	Flint	screen (2 mm)	chips
16/116-151483	7*	Brown	1	Flint	screen (4 mm)	chip
16/116-159227	7/8*		1	Flint	screen (4 mm)	chip
16/116-159257	7/8*		2	Flint	screen (2 mm)	chips
16/116-149964	7/8*	Brown	1	Flint	screen (4 mm)	chip
			**Total: 11**			
**Layer 8**						
16/116-149962	8		1 (broken)	Quartzite	screen (4 mm)	chip
16/116-150357	8		1	Flint	<1 cm	chip
16/116-151453	8		1	Flint	2 cm	blade (proximal fragment)
16/116-159007	8		1	Flint	<1 cm	chip
16/116-159048	8		1	Flint	1-2 cm	blade (medial fragment)
16/116-159051	8		1 (broken)	Quartzite	>1 cm	flake
16/116-159097	8		1	Flint	1-2 cm	blade or flake (distal fragment)
16/116-159108	8		1	Flint	<1 cm	chip
16/116-159156	8		1	Flint	<1 cm	chip
16/116-159163	8		1	Quartzite	1 cm	chip
16/116-159255	8		1	Quartz	1-2 cm	small flake
16/116-159267	8		1	Flint	<1 cm	chip
16/116-151592	8		3	Flint	screen (4 mm)	chips
16/116-151558	8		3	Flint	screen (4 mm)	chips
16/116-151559	8		1	Flint	screen (4 mm)	chip

16/116-151586	8		2	Flint	screen (4 mm)	chips
16/116-159030	8		1	Flint	screen (4 mm)	chip
16/116-159059	8		1	Flint	screen (4 mm)	chip
16/116-159065	8		1	Flint	screen (4 mm)	chip
16/116-159089	8		1	Flint	screen (2 mm)	chip
16/116-159112	8		3	Flint	screen (2 mm)	chips
16/116-159128	8		3	Flint	screen (2 mm)	chips
16/116-159141	8		3	Flint	screen (2 mm)	chips
16/116-159159	8		2	Flint	screen (2 mm, 4 mm)	chips
16/116-159175	8		1	Flint	screen (2 mm)	chip
16/116-159180	8		2	Flint	screen (2 mm)	chips
			**Total: 39**			
**Layer 9**						
16/116-149974	9		1	Flint	screen (4 mm)	chip
16/116-149975	9		1	Rock Crystal	screen (4 mm)	chip
			**Total: 2**			
**Layer 10**						
16/116-149973	10		1	Quartzite	screen (4 mm)	chip
16/116-159360	10		1	N.A. (not Flint)		Hammerstone
			**Total: 2**			
**Layer 11 (currently under analysis)**						
16/116-149971	11		2	N.A. (not Flint)	screen (4 mm)	chips
16/116-149972	11		2	N.A. (not Flint)	screen (2 mm, 4 mm)	chips
16/116-149976	11		1	Greywacke	screen (4 mm), >2 cm	flake
16/116-149977	11		2	N.A. (not Flint)	screen (2 mm)	chips
16/116-149978	11		1	N.A. (not Flint)	screen (2 mm)	chip
16/116-149980	11		2	N.A. (not Flint)	screen (4 mm)	chips
16/116-149983	11		1	N.A. (not Flint)	screen (2 mm)	chip
16/116-149984	11		1	N.A. (not Flint)	screen (4 mm)	chip
16/116-186170	11		1	Quartz (crystalline)	1 cm	chunk
16/116-186259	11		1	Quartz (crystalline)	1-2 cm	small flake

16/116-186387	11		1	Quartz (crystalline)	1 cm	small flake
16/116-186555	11		1	N.A. (not Flint)	1 cm	chip
16/116-189159	11		1	N.A. (not Flint)	screen (2 mm)	chip
16/116-189325	11	Brown	1	Quartz	screen (4 mm)	chip
16/116-186458	11		1	N.A. (not Flint)	screen (4 mm)	chip
16/116-189177	11		2	Carneol?, Greywacke	screen (4 mm)	chips
16/116-189152	11		1	Greywacke?	screen (4 mm)	chip
			**Total: 22**			

1.3. Micromorphology of layers 7 to 9

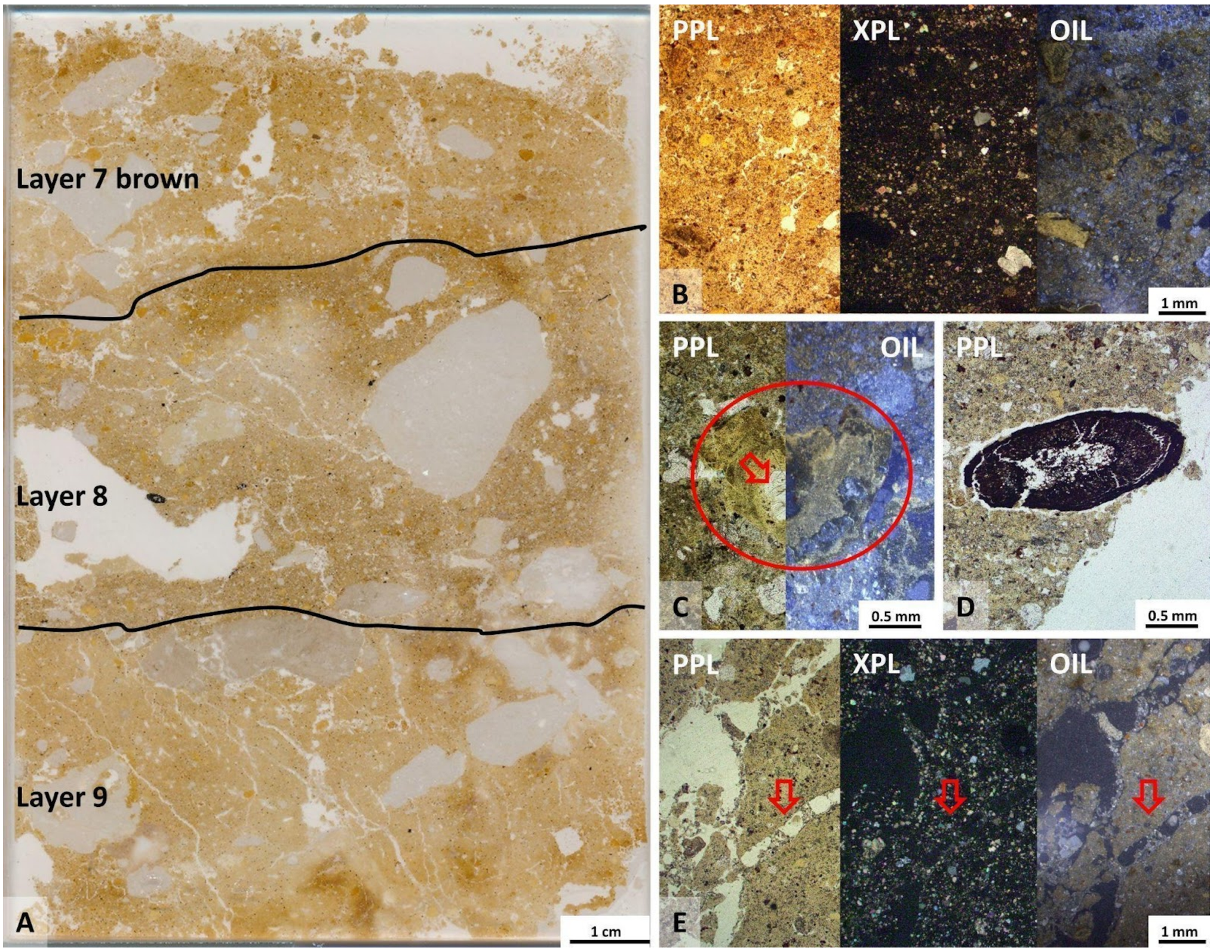

Figure S9. Micromorphological analysis of thin section RNS 20 4 containing layers 7 Brown, 8 and 9.

Here we focus on the thin section of layers 9, 8 and 7 Brown. In the North Profile, where the micromorphology sample was taken, the thin layer 7 Brown was overlying layer 8. Micromorphological analysis were performed following Stoops 2003[34], 2021[35], Nicosia and Stoops, 2017[36] and the thin section was produced by MKfactory, Stahnsdorf, Germany. **A:** Scan of thin section RNS 20 4 containing layers 7 Brown, 8 and 9 showing clear contacts and colour differences (local light and dark patches present thin section production artefacts). layer 9 shows a light brown colour with common subvertical planes/fissures, layer 8 exhibits a spotted greyish colour with some sub-horizontal planes and layer 7 Brown again a light brown colour. Note the small brown spots presenting clay aggregates, carnivore coprolites or bones. They are most frequent in layer 7 Brown. Micromorphological analysis revealed that the 3 layers contain the same materials while their proportions differ between them, and they also exhibit structural differences. Furthermore, the analysis show that the bulk of the sediments in layers 9 to 7 is autochthonous (weathering of the bedrock) with a minor aeolian (quartz and mica), biogenic (coprolites) and anthropogenic (bones, charred organic material) contribution. Disturbance of the sediments is minimal with few planes, fissures and bioturbation traces. **B:** Microphoto layer 7 Brown in plane (PPL), cross-polarized (XPL) and oblique incident (OIL). The fine material is composed of calcitic crystallitic yellowish brown

clay. Coarse materials include limestone sand and gravel stemming from the bedrock, sand to silt-sized quartz grains and mica as aeolian input as well as brown aggregates, most likely presenting hyaena coprolites[33]. The microstructure is massive with few planes indicating limited disturbance. **C:** Microphoto layer 8 with a coprolite fragment (red circle) containing a bone fragment (red arrow), a typical inclusion of hyaena coprolites. **D:** Charcoal in layer 8. Note also the finely commuted dark spots and greyish hue of the massive clay matrix, presenting dark amorphous staining, microcharcoal and charred plant residues indicating increased fire frequency during the deposition of layer 8 compared to layers 9 and 7. This is additional evidence that layer 8 represents the main occupation of the LRJ. **E:** Microphoto layer 9 showing a calcitic crystallitic yellowish brown clay, iron staining of the matrix and common gravel to sand limestone fragments and sand to silt sized quartz grains. Also note the occurrence of a channel filled with needle fiber calcite (red arrow).

1.4. Sedimentological results layer 7 to 9

Sediment samples were collected from the various archaeological layers. Within a certain layer several samples were taken in case of recognisable sedimentological changes (e.g. colour changes). 31 samples were taken from the entire Ranis sequence. For the aim of this study, we analysed the LRJ layers 9 and 8, as well as three samples from the overlying layer 7 (7 Top, 7 Mid, and 7 brown). In a first step air-dried samples were separated into coarse fraction (> 2.0 mm) and fine-earth fraction (≤ 2.0 mm) by sieving. Further analysis were performed using the fine-earth fraction.

1.4.1. Carbon and nitrogen

Total carbon (TC) and total nitrogen (TN) was measured on milled fine-earth samples by dry combustion. An CN-analyser (Elementar vario EL cube) with heating temperatures > 1100°C was used for the determination. Organic carbon (OC) was determined by loss on ignition (LOI) using a Microwave muffle furnace (CEM Phoenix). The loss in mass was divided by the Van Bemmelen constant (1.724) to derive the OC[37,38]. To avoid over-estimates of LOI through structural water loss, a correction procedure was applied subtracting 0.1 mass-percent per one percent clay[37]. Three replicates were measured to ensure reliable results. Inorganic carbon (IC) was calculated by subtracting OC from TC. The C/N ratio was calculated by division (OC/TN).

TC was in the same range (5.12 to 5.78%) in all five analysed sediment samples. The calculated IC was relatively high (1.93 to 3.78%), whereas layers 8 and 7 showed a similar IC content (3.40 to 3.78%), decreasing to 1.93% in layer 9. In contrast, OC showed larger variations and increased markedly from 1.57% and 1.37% (layer 7 Top and Mid) to 2.61% (layer 7 brown), 2.39% (layer 8) and subsequently to 3.21% (layer 9). Similarly, TN was markedly increased in layer 9 (0.42%), whereas layers 8 and 7 revealed similar values between 0.09 and 0.22%. The calculated C/N ratio was increased in layers 8 and 7 (11.86 to 15.6) and strikingly reduced in layer 9 (7.74).

The results indicated an environmental succession from layer 9 to layer 7 with OC and TN values reduced to half and one fourth in layer 7, respectively. MIS 3 was characterised by climatic fluctuations and the interplay between cold stadials and warmer interstadials[39], the latter characterised by a denser vegetation cover and an increased biomass production. The relatively high percentage of OC and TN in layer 9 therefore gave a hint to slightly warmer conditions (interstadial conditions). The decreasing percentages of OC and TN from layer 9 to 7 therefore might have represented a temperature decline to colder climatic conditions. Overall, the archived OC values from the layers 9 to 7 indicated a high biomass production during the formation and consequently, it could be assumed that the excavated area was located close to the cave entrance.

1.4.2. Grain size analysis

Grain size analysis was carried out by a combination of the Köhn-pipette method (silt and clay fractions) and wet sieving (sand fractions). Samples were pre-treated with 17.5% hydrogen peroxide (H_2O_2) to remove OC. $CaCO_3$ was not removed from the samples following several sedimentological studies from carbonate environments[40,41]. Afterwards samples were pre-treated with 0.4 M sodium pyrophosphate ($Na_4P_2O_7$) overnight and

afterwards shaken for six hours. The sand fraction was divided into coarse (2000-630 µm), medium (630-200 µm) and fine (200-63 µm) sand by wet sieving using a vibratory sieve shaker AS 200 (Retsch). Coarse (63-20 µm), medium (20-6.3 µm), fine silt (6.3-2.0 µm) and clay (<2.0 µm) were identified by the Köhn-pipette method using the SEDIMAT 4-12 (UGT). Based on grain size analysis, soil texture was classified according to Jahn et al. 2006[42].

Grain size analysis revealed a sediment texture generally dominated by the silt (especially cSi) and sand (especially fS) fractions, whereas the clay content was comparably low (Tab. S4, SI). The mean total sand content was 39.78% in samples from layer 7 and 8 decreasing to 36.46% in layer 9. In contrast, layer 9 showed a slightly higher total silt content (52.81%) which reduced in layers 8 and 7 to values between 46.39% and 49.38%. The texture class was classified as loam (layer 8 and 7) to silt-loam (layer 9). The considerable proportion of fine sand (20.07 to 22.38%) and coarse silt (21.90 to 24.45%) might have indicated an partially aeolian sediment input into the cave during time of occupation but the fine sand and silt fraction could also derive from the limestone weathering. Further analysis are required to better clarify this.

Table S4: Sedimentological results.

		grain size [%]										carbon and nitrogen [%]				
**layer**	**coarse fraction (> 2.0 mm)**	**coarse sand**	**medium sand**	**fine sand**	**∑sand**	**coarse silt**	**medium silt**	**fine silt**	**∑silt**	**clay**	**texture class**	**total carbon**	**organic carbon**	**inorganic carbon**	**total nitrogen**	**C/N ratio**
7 (Top)	46.4	9.56	10.45	20.07	40.07	23.08	15.12	8.68	46.85	13.08	L (loam)	5.35	1.57	3.78	0.12	13.55
7 (Mid)	40.0	7.91	9.99	21.91	39.81	24.28	16.73	8.37	49.38	10.81	L (loam)	5.12	1.36	3.76	0.09	15.60
7 (brown)	28.8	8.54	9.23	21.21	38.98	24.00	16.74	8.27	49.01	12.01	L (loam)	5.71	2.61	3.10	0.22	11.86
8	33.3	7.84	10.05	22.38	40.27	21.90	15.37	9.13	46.39	13.35	L (loam)	5.78	2.39	3.40	0.18	12.98
9	30.0	7.02	8.93	20.52	36.46	24.45	17.14	11.22	52.81	10.72	SiL (silt loam)	5.14	3.21	1.93	0.42	7.74

1.5. Analysis of the 1930s collection

The new human remains identified in the collection from the 1930s were found among faunal remains contained in boxes labelled with their provenience, including the square, layer, depth, and date of excavation (Tab. S5, SI). Note that squares 51A and 114 are the same square (named 51A when excavated in 1934, and 114 from 1935). For R10875, the date of excavation was assumed from the reading of Hülle (1977)[15] where he reported on his fieldwork year by year. The layers indicated on the fauna boxes were the layer names listed in the column "Layer" of Tab. S5, SI. We translated them into layer numbers (column "Layer #") based on the correspondence between the names and excavation information found in Hülle (1977)[15]. Several of the human remains came from boxes containing a mix of materials from two different layers. For R10875, the correspondence between the layer names and layer numbers was not straightforward hence the question mark after "X-XI". However, it was the most parsimonious interpretation based on the reading of Hülle (1977)[15]. The direct dates of the four human remains coming from boxes with material from different layers overlap with those obtained on materials from layer X, which points to their attribution to layer X as well (see main text).

Table S5. List and provenience of the human remains (R10318, R10355, R10873, R10396, R10400, R10874, R10875, R10876, R10879) identified in the old collection at Ranis along with the lithic artefacts (2, xx) found in layer X in the same squares.

ID #	Identification	Raw material	Date	Site area	Square	Layer	Layer #	Depth (m)
**R10318**	**Rib shaft fragment**		**11.09.1934**		**51A**	**graue Schicht**	**X**	**6.00 - 6.15**
**R10355**	**Rib shaft fragment**		**11.09.1934**		**51A**	**graue Schicht**	**X**	**6.00 - 6.15**
2, 32	Jerzmanowice point (fragment)	flint	11.09.1934		51A		X	6.00 - 6.10
**R10396**	**Long bone diaphysis fragment**		**23.08.1935**		**164**	**graue Schicht**	**X**	
2, 26	Jerzmanowice point / pointed blade	flint	23.08.1935		164		X	5.75
2, 60	Jerzmanowice point	flint	23.08.1935		164		X	5.85
2, 78	Blade fragment	quartzite	23.08.1935		164		X	5.70
**R10400**	**Long bone diaphysis fragment**		**10/11.09.1934**		**51**	**graue Schicht**	**X**	**5.90 - 6.05**
2, 46	Bifacial leaf point	flint	11.09.1934		51		X	5.90 - 6.05
**R10873**	**Metacarpal 1, distal extremity**		**14.08.1935**		**114**	**mittlere schokobraune und graue Schicht**	**IX-X**	
2, 36	Jerzmanowice point (fragment)	flint	15.08.1935		114		X	5.80
2, 37	Jerzmanowice point (fragment)	flint	15.08.1935		114		X	5.95
2, 52	Bifacial leaf point	flint	15.08.1935		114		X	5.65
2, 54	Bifacial leaf point	flint	15.08.1935		114		X	5.80
**R10874**	**Clavicle**		**23.08.1937**	**Fläche A 37**	**101/102/107**	**graue Schicht**	**X**	
2, 31	Pointed blade	flint	24.08.1937	Fl. A	107		X	4.15
2, 56	Bifacial leaf point	flint	1937		101		X	4.00
**R10875**	**Hand proximal phalanx**		**1934?**	**Spalte A37**	**14C**	**graue & schokobraune Schicht**	**X-XI?**	**5.60 - 6.20**

**R10876**	**Clavicle**		**02.07.1934**	**Spalte A37**	**19A**	**untere Schoko- & graue Schicht**	**X-XI**	**6.00 - 6.70**
**R10879**	**Femur diaphysis fragment**		**21/22.08.1935**		**144/154**	**graue und untere braune Schicht**	**X-XI**	
2, 42	Bifacial leaf point (fragment)	flint	20.08.1935		144		X	5.45
2, 43	Jerzmanowice point (fragment)	flint	21.08.1935		144		X	5.50
2, 29	Pointed blade	flint	21.08.1935		144		X	5.75
2, 59	Jerzmanowice point	flint	21.08.1935		144		X	5.75
2, 24	Jerzmanowice point	flint	22.08.1935		154		X	5.75

2. Proteomic screening

We screened 1,322 non-identifiable bone specimens recovered from the layers 7, 7/8, 8, 9, 10, 11, 12, in the 2016-2022 excavation, and layers X + IX ("Graue + Untere Schokoschicht"), layer X ("Graue Schicht"), layer IX + X ("Schokobraune + Graue Schicht") from the 1932-1938 excavation (note that if two layer names are given, these bones come from mixed boxes) using ZooMS[43]. A subset of 341 bone specimens, recovered only from the LRJ layers (layers 9 and 8 from the 2016-2022 excavation and layer X from the 1932-1938 excavation), was also analysed using SPIN[44] to identify hominin remains. Additional aims were to provide more accurate molecular identifications for radiocarbon-dated specimens and to enlarge our understanding of the faunal species composition. The material recovered from Ranis was generally well preserved which allowed us to perform both types of proteomic analytical approaches.

2.1. Zooarchaeology by Mass spectrometry (ZooMS)

Faunal ZooMS identifications revealed that Ursidae (most likely cave bears (*Ursus spelaeus*) were the most abundant species at Ranis[33]. Ursidae (number of identified specimens, NISP = 258), reindeer (*Rangifer tarandus*, NISP = 155) and Equidae (NISP = 136) were identified in all studied layers among the material from the 2016-2022 excavation (Tab. S23, SI). The indeterminate specimens (specimens for which a taxonomic identification could not be obtained) represent 4.3% of the studied part of the assemblage. Within the faunal material from the 1932-1938 excavation, Ursidae (NISP = 276), reindeer (NISP = 248) and Equidae (NISP = 90) were the most abundant taxa in all studied layers. Several carnivore species were also obtained within each layer (Tab. S24, SI). Here, indeterminate specimens represent 9.8% of the total number of bone fragments analysed. Extraction blanks were added during every step of the analysis to evaluate contamination. MALDI-TOF-MS analysis of these blanks showed no presence of COL1 peptides (Tab. S6, SI).

2.2. Species by Proteome INvestigation (SPIN)

The required DIA peptide identification data for SPIN was generated with Spectronaut[45,46]. SPIN spectra were analysed without a spectral library by directDIA (DirDIA) and with a spectral library-based DIA (libraryDIA) approach. LibraryDIA is a DIA approach using a dedicated spectral library generated for several species of interest by DDA analysis of peptide extracts from specimens with known taxonomic identities[47]. Final SPIN species assignments were based on an alignment around an amino acid site-based species comparison, which selected the highest number of identified peptide precursors (site counts, the absolute sequence coverage) per sample between the first and the second-best matching species, including filtering based on the relative protease intensity of each .raw file. We performed several types of Spectronaut data analysis for taxonomic identification (341 specimens analysed in a combined search, separated search by extraction plate and search of low-quality specimens re-analysed in the LC-MS/MS). Even though in Ruether et al. 2022[44], the Spectronaut search was performed in all analyzed samples in the entire study in the species inference pipeline together, for this study we concluded that separate Spectronaut

searches by extraction plate were more efficient. The manual selection of taxonomic identification derived from the separate Spectronaut searches by plate was based on the highest number of site counts and generated the most reliable conclusions, reducing the number of non-compatible identifications.

Molecular contamination is an important issue when studying ancient biomolecules, especially when it concerns ancient hominins. Extraction blanks were included throughout all analysis stages to monitor the introduction of potential contamination during the laboratory analysis. We observed carryover on the first analysed blanks, but with a low signal, unlikely to influence the taxonomic identifications for sample extracts (Tab. S6, SI). However, one lab blank (Plate Blank_103_03) of the SPIN approach was probably mixed up or cross-contaminated while processing due to high number of site counts (>2500) and taxonomic identification as a cave bear.

As SPIN is a recently developed approach, the protein sequence reference database as well as the library DDA datasets, are incomplete for studying Late Pleistocene bone assemblages, which are often composed of a large variety of mammalian taxa. For example, in the existing SPIN database, only *Odocoileus virginianus* is present as a member of the cervids (Cervidae). Consequently, further discrimination cannot be performed among the cervids. As a result, this identification could indicate any member of this family. Assignment to the genera *Rangifer*, *Alces*, *Capreolus*, *Cervus*, *Megaloceros*, or *Dama* is therefore possible.

SPIN results assigned most of the specimens to the same species as ZooMS (Tab. S7, SI), resulting in 79% directly compatible taxonomic identifications of the analysed specimens for layers X (1932-1938 excavation) and 8 (2016-2022 excavation); Fig. S11, SI). For example, we observed that cave bears and cervids were abundant in specimens analysed by SPIN, in agreement with the ZooMS data. Moreover, SPIN provided more specific identifications even among subfamily groups or genera. For example, within bovines, the European bison (*Bison bonasus,* 10 Number of Individual SPecimens; NISP) could be distinguished from the domestic cattle (*Bos taurus*) and from the aurochs (*Bos primigenius*), but not from American bison (*Bison bison*), yak (*Bos mutus*) and zebu *(Bos taurus indicus*). Within equines, caballine horses, represented by the domestic horse (*Equus ferus caballus*) and the Przewalski horse (*Equus ferus przewalskii* or *Equus przewalskii*), can be discriminated from asine horses, represented by the donkey (*Equus africanus asinus*), which cannot be done by standard ZooMS collagen PMF approaches[44]. At Ranis, this allowed us to establish a dominant presence of caballine horses (15 NISP) compared to asine horses (1 NISP), with some specimens assigned to the genus *Equus* generally (9 NISP).

Among the fauna remains, we recovered 56 specimens with non-compatible taxonomic identifications between ZooMS and SPIN, and 14 specimens that were not assigned a taxonomic identity with SPIN (Tab. S8 and Fig. S11, SI). Specimens that were identified only by one method and where the other one provided a low signal spectrum are counted as NA. The conflicting identifications are possibly related to the absence of the relevant species in the SPIN reference database (Fig. S12, SI). This might be explained due to the variety in completeness and quality of protein sequence databases between taxa. Additionally, PTMs, missing genes, gaps, and stretches of incorrect amino acid sequences can introduce a bias towards well-annotated species. In most of the cases, in SPIN results, 38

specimens were assigned to the wrong species, or their phylogenetically closest group represented in the database. In addition, 13 of them were falsely identified due to database limitation, cross-contamination or low-quality spectra, and 5 of the conflicted identifications were potential sample mix-up or low signal spectra. Approximately 84% of the rhinos, carnivores and cervids map to the wrong group species because the reference database (either DirDIA or LibDIA) is incomplete for this order, so far. For example, 34 specimens were identified as Rhinocerotidae by ZooMS. As this family is absent from the SPIN database, SPIN was not able to assign their taxonomic identities correctly. Instead, 26 (76%) were assigned to a member of the horse family (Equidae), which represents the phylogenetically closest entry in the searched database. Two of the identified Rhinocerotidae by ZooMS had low signal in SPIN, while five specimens were assigned to Urisdae and one to Mustelidae by SPIN. Likewise, the absence of various carnivore and cervid entries in the SPIN database and existing DDA libraries is the likely root cause of the large number of incompatible identifications found for SPIN, when compared to ZooMS identifications. An updated reference database is therefore clearly required in order to optimise future analysis using SPIN.

2.3. Hominins

From this study we recovered eight hominin bone specimens with proteomic analysis. Three of them came from layer 8 and one came from layer 9 of the 2016-2022 excavation. Four hominin specimens were located in layer X from the 1932-1938 excavation. The hominin bone fragment from layer 9 was not included in the SPIN analysis (Extended Data Tab. 1, main text). In all other cases (n = 7), SPIN and ZooMS both identified the generated peptide extracts as belonging to the genus *Homo*. One additional specimen was analyzed twice with SPIN and was identified as Ursidae first and then as *Pan paniscus/Pan troglodytes* through this method, while no taxonomic identity could be assigned through ZooMS. Based on these conflicting SPIN identifications, the low amount of protein sites assigned confidently, especially the relative protease intensity, we did not process this specimen further for aDNA or radiocarbon dating analysis. For SPIN, the existing reference database only contains *Homo sapiens* in the context of hominins and, as a result, this identification should be taken as implying hominin. Therefore, specimens identified as "*Homo sapiens*" in the SPIN approach, both DirDIA and LibDIA, were tentatively assigned to the genus *Homo*. These specimens could therefore represent other hominin populations, such as Neanderthals, based on their proteomic taxonomic identification.

2.4. Deamidation

Deamidation is a post-translational modification (PTM) that refers to the loss of an amide group, which converts asparagine to aspartic acid, and glutamine to glutamic acid, resulting in a +0.98 Da mass shift[48]. Values displaying the stage of conversion of the unmodified to the modified variant have been used as an indicator of molecular collagen type I preservation. Some studies have suggested that deamidation is a potential approach to distinguish between contaminant and authentic ancient proteins within complex proteomes[49–54]. For ZooMS, deamidation values were calculated based on the collagen type I

deamidation for COL1α1 508-519 isotopic multiplets of both peptide variations (glutamine/Q and glutamic acid/E), that overlap in MALDI-TOF spectra[49]. The Q deamidation results in a shift of the isotope distribution and the second peak in the isotope distribution for a peptide containing one Q residue coincides with the first peak of the isotope distribution for the peptide in which the residue is deamidated[54]. For ZooMS, a value of 1 would indicate no deamidation while a value of 0 would indicate complete deamidation. For SPIN, deamidation values were calculated by site-specific deamidation of proteins within a sample[55]. Differences in deamidation rate of glutamine (Q) and asparagine (N) based on the presence of specific neighbouring amino acids[56]. Deamidation for SPIN analysis was carried out based on the directDIA results generated by Spectronaut search for each plate separately by counting the occurrences of deamidated asparagine and glutamine residues, respectively, divided by the total occurrence of deamidated and undeamidated asparagine and glutamine residues, respectively. Calculated values are therefore a PSM-based ratio, not taking into account peptide intensities. For SPIN based Spectronaut output, a value of 0 would indicate no measurable deamidation, while a value of 1 would indicate complete deamidation of glutamine or asparagine residues. Deamidation analysis of both proteomic datasets (ZooMS and SPIN) suggests the presence of diagenetically altered collagen and proteomes (Fig. S10, SI).

We analysed the deamidation rates for the fauna and the hominins present at Ranis using both proteomic approaches, divided between the 2016-2022 and 1932-1938 excavations. Detailed analysis of the generated deamidation values revealed that in our study the DirDIA deamidation values were similar to the LibDIA approach and rerun .raw files. Protein degradation for SPIN was measured by the presence of identified deamidated sequence per specimen both for Q and N amino acids. Deamidation rates for ZooMS were 0.74 ± 0.07 S.D. and 0.68 ± 0.09 S.D. for the 2016-2022 and 1932-1938 excavations respectively (F value = 21.251, Pr(>F) < 2.2e-16; Fig. S10a, SI). Deamidation percentage for Q using SPIN were 0.51 ± 0.02 S.D. and 0.53 ± 0.02 S.D. for the 2016-2022 and 1932-1938 excavations respectively based on the DirDIA results (F value = 56.854, Pr(>F) = 4.668e-13; Fig. S10b, SI). Deamidation percentage for N using directDIA SPIN were 0.43 ± 0.02 S.D. and 0.49 ± 0.04 S.D. for the 2016-2022 and 1932-1938 excavations respectively (F value = 478.568, Pr(>F) < 2.2e-16; Fig. S10c, SI). For all the hominin bones found in all layers the average deamidation values were 0.73 ± 0.07 S.D. for ZooMS analysis, Q deamidation 0.52 ± 0.02 S.D. for SPIN directDIA and N deamidation 0.43 ± 0.02 S. D for SPIN directDIA (Extended Data Fig. 4, main text). The deamidation average for both proteomic approaches for the hominins fell between the deamidation values of the fauna remains from both the 2016-2022 and 1932-1938 excavations and suggested that the hominin specimens have experienced the same diagenetic influence as the fauna specimens with which they were recovered.

Table S6. Blank identifications for both proteomic approaches ZooMS and SPIN.

Specimen Name	ZooMS ID	SPIN ID
Plate Blank_86_01	signal too low	-
Plate Blank_87_01	signal too low	-
Plate Blank_88_01	signal too low	-
Plate Blank_94_01	signal too low	-
Plate Blank_102_01	-	*Felis catus*
Plate Blank_103_01	-	signal too low
Plate Blank_103_02	-	below FDR
Plate Blank_103_03	-	*Ursus americanus;Ursus arctos*
Plate Blank_104_01	-	signal too low
Plate Blank_104_02	-	below FDR
Plate Blank_104_03	-	signal too low
Plate Blank_104_04	-	*Ursus americanus;Ursus arctos;Ursus maritimus*
Plate Blank_105_01	-	*Ursus americanus;Ursus arctos;Ursus maritimus*

Table S7. List of taxonomic identifications by ZooMS and SPIN and their respective counts for the entire proteomic dataset for layers X (1932-1938 excavation), 9 and 8 (2016-2022 excavation).

SPIN results assigned most of the specimens to the same species as ZooMS. SPIN was performed only in a subset of 336 specimens, 17% of them were not compatible between the ZooMS and SPIN approaches and 4% of the analysed specimens did not receive a taxonomic assigned by either or both proteomic methods. The conflicting identifications are related to the absence of the relevant species in the SPIN reference database (SPIN), or cross-contamination of proteomic extractions (ZooMS and SPIN), or carryover during the LC-MS/MS runs (SPIN).

ZooMS ID	SPIN ID	Count
Elephantidae	*Felis catus*	1
Elephantidae	*Loxodonta africana*	3
Elephantidae	*Loxodonta africana;Ursus arctos*	1
Elephantidae	*Loxodonta africana;Ursus arctos;Ursus maritimus*	1
Rhinocerotidae	below FDR	1
Rhinocerotidae	*Equus asinus*	12
Rhinocerotidae	*Equus asinus;Equus caballus;Equus przewalskii*	14
Rhinocerotidae	*Mustela erminea;Mustela putorius*	1
Rhinocerotidae	*Odocoileus virginianus*	1
Rhinocerotidae	signal too low	1
Rhinocerotidae	*Ursus americanus;Ursus arctos*	2
Rhinocerotidae	*Ursus americanus;Ursus arctos;Ursus maritimus*	1
Rhinocerotidae	*Ursus arctos*	1
Equidae	*Bison bison;Bison bonasus;Bos indicus;Bos mutus*	1
Equidae	*Bison bison;Bison bonasus;Bos indicus;Bos mutus;Bos primigenius;Bos taurus;Bubalus bubalis;Odocoileus virginianus*	1
Equidae	*Equus asinus*	1
Equidae	*Equus asinus;Equus caballus;Equus przewalskii*	9
Equidae	*Equus caballus;Equus przewalskii*	15
Equidae	*Odocoileus virginianus*	2
Felinae	*Lynx canadensis;Lynx pardinus*	1
Canidae (not vulpes vulpes)	*Canis lupus*	1
Canidae (not vulpes vulpes)	*Vulpes vulpes*	1
Ursidae	*Equus asinus;Equus caballus;Equus przewalskii*	2
Ursidae	*Equus caballus;Equus przewalskii*	1
Ursidae	*Odocoileus virginianus*	1
Ursidae	signal too low	2
Ursidae	*Ursus americanus;Ursus arctos*	33
Ursidae	*Ursus americanus;Ursus arctos;Ursus maritimus*	2
Ursidae	*Ursus arctos*	56

Ursidae	*Ursus arctos;Ursus maritimus*	5
Rangifer	*Bison bison;Bison bonasus;Bos indicus;Bos mutus;Bos primigenius;Bos taurus*	5
Rangifer	*Capra hircus;Odocoileus virginianus*	1
Rangifer	*Equus caballus;Equus przewalskii*	1
Rangifer	*Odocoileus virginianus*	82
Rangifer	signal too low	1
Rangifer	*Ursus arctos*	1
Cervid/Saiga	*Odocoileus virginianus*	15
Cervid/Saiga/Capreolus	*Odocoileus virginianus*	4
Cervid/Saiga/Capreolus	*Ursus americanus;Ursus arctos*	1
Rangifer/Caprinae	*Odocoileus virginianus*	1
Bos/Bison	*Bison bison;Bison bonasus;Bos indicus;Bos mutus*	10
Bos/Bison	*Bison bison;Bison bonasus;Bos indicus;Bos mutus;Bos primigenius;Bos taurus*	10
Bos/Bison	*Equus caballus;Equus przewalskii*	1
Bos/Bison	signal too low	1
Homo sp.	*Homo sapiens*	7
Felinae/Ursidae	*Ursus americanus;Ursus arctos*	1
Hyaenidae/Felidae	*Acinonyx jubatus;Lynx pardinus;Puma concolor*	1
Hyaenidae/Felidae	*Lynx canadensis;Lynx pardinus*	1
Hyaenidae/Pantherinae	*Acinonyx jubatus;Lynx canadensis;Lynx pardinus*	1
Hyaenidae/Pantherinae	*Felis catus;Puma concolor*	1
Hyaenidae/Pantherinae	*Lynx canadensis*	1
Hyaenidae/Pantherinae	*Lynx canadensis;Lynx pardinus*	4
Hyaenidae/Pantherinae	signal too low	1
Carnivora	*Canis lupus*	1
Carnivora	*Ursus arctos*	1
Carnivora	*Vulpes vulpes*	1
Sample mix-up	*Equus asinus;Equus caballus;Equus przewalskii*	1
Indeterminate	*Odocoileus virginianus*	1
Empty	*Equus asinus;Equus caballus;Equus przewalskii*	1
Empty	*Odocoileus virginianus*	2
Empty	*Pan paniscus;Pan troglodytes*	1
Empty	*Ursus americanus;Ursus arctos*	1

**TOTAL**		**336**

Table S8. List of non-compatible taxonomic identifications between ZooMS and SPIN and their respective counts for layers X (1932-1938 excavation), 9 and 8 (2016-2022 excavation).

We observed 56 fauna specimens with non-compatible taxonomic identifications. Low quality spectra (ZooMS and SPIN) were characterised as NA. The conflicting identifications are possibly related to the absence of the relevant species in the SPIN reference database (SPIN), cross-contamination during the extraction process (ZooMS and SPIN) or carryover between LC-MS/MS runs (SPIN).

ZooMS ID	SPIN ID	Count
Elephantidae	*Felis catus*	1
Rhinocerotidae	*Equus asinus;Equus caballus;Equus przewalskii*	13
Rhinocerotidae	*Equus asinus*	12
Rhinocerotidae	*Ursus americanus;Ursus arctos*	2
Rhinocerotidae	*Mustela erminea;Mustela putorius*	1
Rhinocerotidae	*Odocoileus virginianus*	1
Rhinocerotidae	*Ursus americanus;Ursus arctos;Ursus maritimus*	1
Rhinocerotidae	*Ursus arctos*	1
Equidae	*Odocoileus virginianus*	2
Equidae	*Bison bison;Bison bonasus;Bos indicus;Bos mutus*	1
Equidae	*Bison bison;Bison bonasus;Bos indicus;Bos mutus;Bos primigenius;Bos taurus;Bubalus bubalis;Odocoileus virginianus*	1
Canidae (not vulpes vulpes)	*Vulpes vulpes*	1
Ursidae	*Equus asinus;Equus caballus;Equus przewalskii*	2
Ursidae	*Equus caballus;Equus przewalskii*	1
Ursidae	*Odocoileus virginianus*	1
Rangifer	*Bison bison;Bison bonasus;Bos indicus;Bos mutus;Bos primigenius;Bos taurus*	5
Rangifer	*Equus caballus;Equus przewalskii*	1
Rangifer	*Ursus arctos*	1
Cervid/Saiga/Capreolus	*Ursus americanus;Ursus arctos*	1
Bos/Bison	*Equus caballus;Equus przewalskii*	1
Hyaenidae/Pantherinae	*Lynx canadensis;Lynx pardinus*	4
Hyaenidae/Pantherinae	*Acinonyx jubatus;Lynx canadensis;Lynx pardinus*	1
Hyaenidae/Pantherinae	*Lynx canadensis*	1
**TOTAL**		**56**

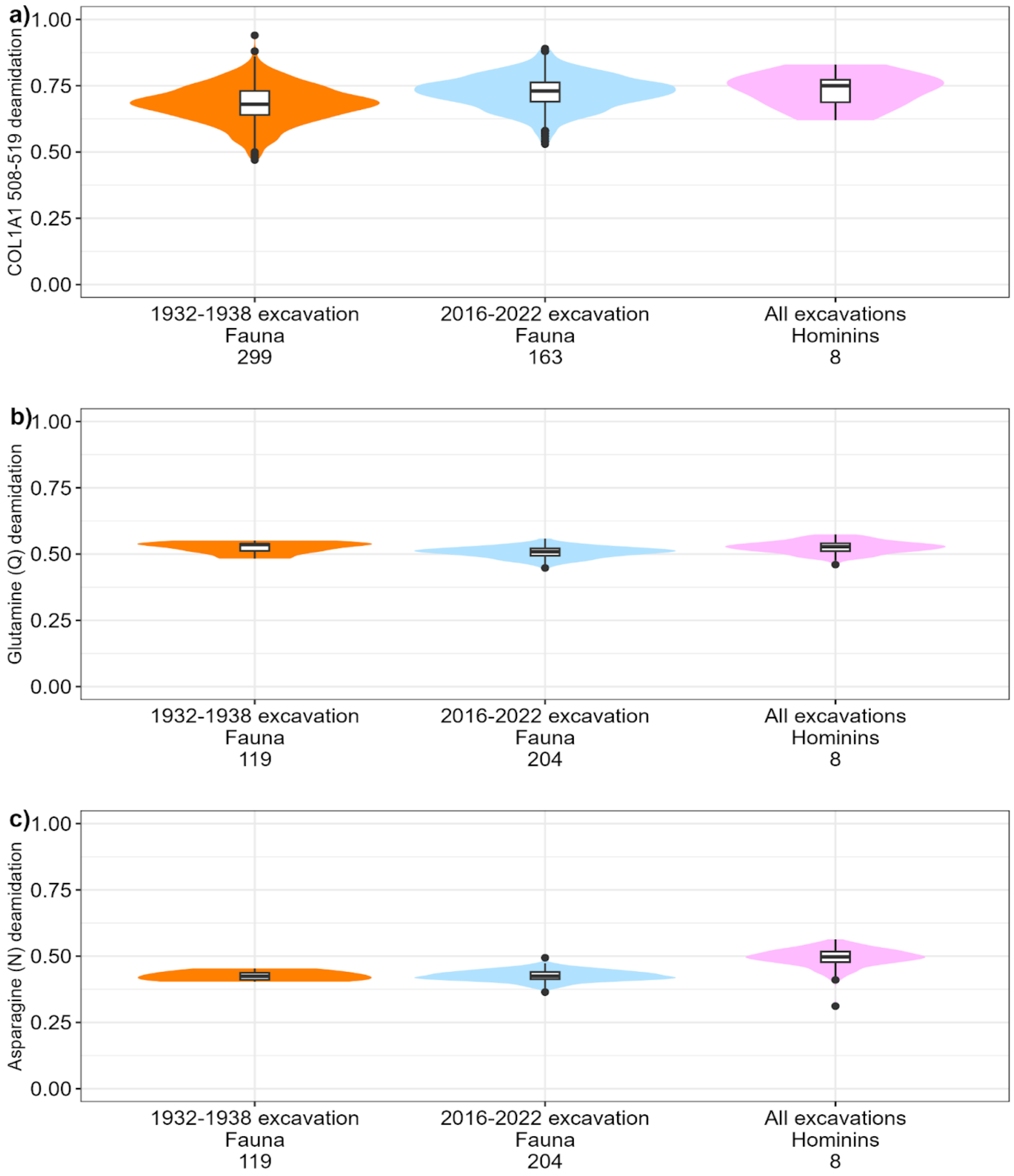

Figure S10. Protein degradation for the archaeological bone specimens with ZooMS and directDIA SPIN for layers X (1932-1938 excavation), 9 and 8 (2016-2022 excavation).
a) Deamidation rate for the 1932-1938 (orange) and 2016-2022 (light blue) excavations faunal remains, and for all the hominin remains (pink) from all excavations analysed with ZooMS. 131 specimens analysed by ZooMS generated non-finite values and were removed from the violin plot by default, **b)** Glutamine (Q) deamidation values for the 1932-1938 (orange) and 2016-2022 (light blue) excavations faunal remains, and for all the hominin remains (pink) from all excavations, analysed with directDIA SPIN. and **c)** Asparagine (N) deamidation values for the 1932-1938 (orange) and 2016-2022 (light blue) excavations faunal remains, and for all the hominin remains (pink) from all excavations, analysed with directDIA

SPIN. For panel a, 1 indicates no deamidation of N or Q, while 0 indicates complete deamidation of N or Q. For panels b and c, 0 indicates no deamidation of N or Q, while 1 indicates complete deamidation of N or Q. The box plots within the violin plots define the range of the data (whiskers extend to 1.5× the interquartile range), outliers (black dots, beyond 1.5× the interquartile range), 25th and 75th percentiles (boxes), and medians (centre lines). The sample size (*n*=) used to derive statistics for each boxplot within the violin plots is shown in the x- axis.

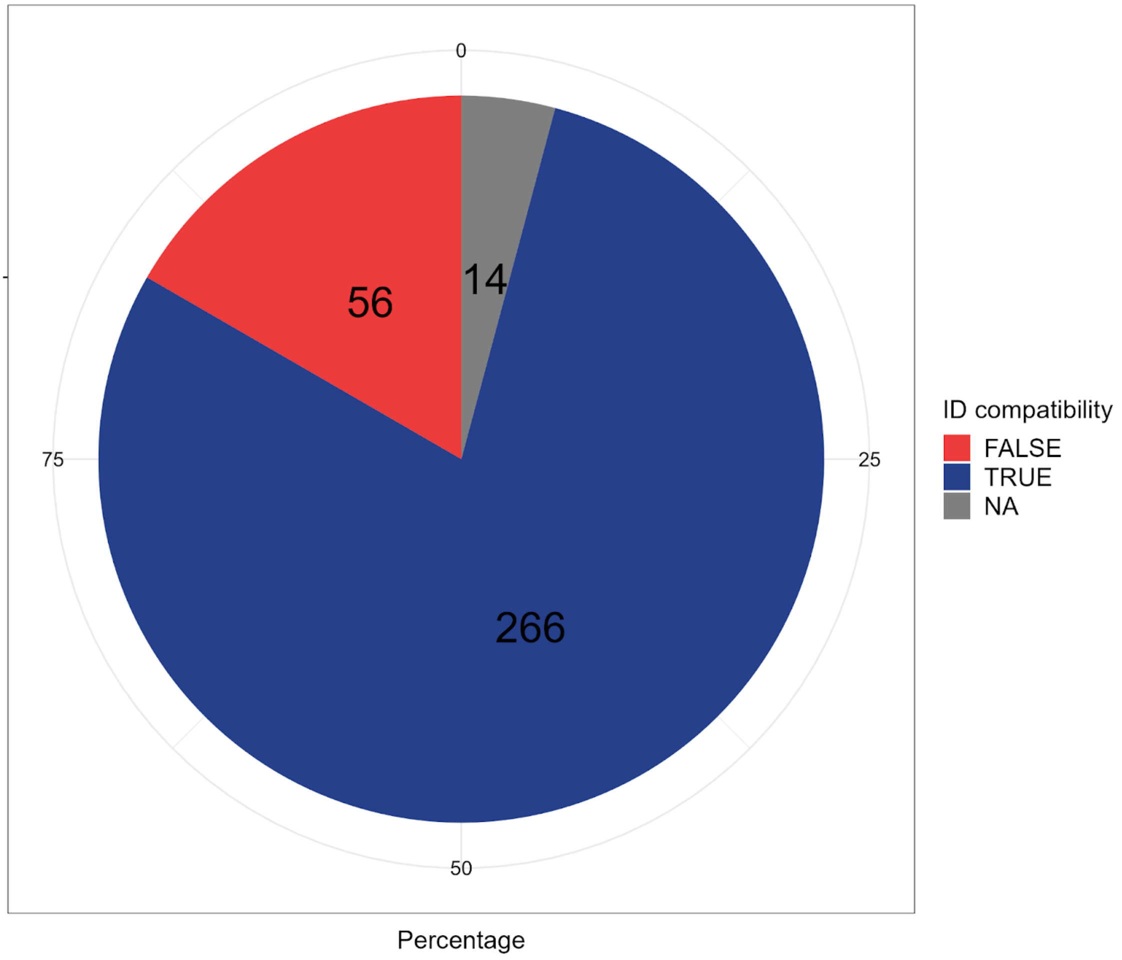

Figure S11. Pie chart of taxonomic identification obtained through ZooMS and SPIN and their compatible/non-compatible status for layers X (1932-1938 excavation), 9 and 8 (2016-2022 excavation).

SPIN results assigned most of the samples to the same species as ZooMS. Specimens that were identified by only one method while the other method provided a low signal spectrum are characterised as NA. Numbers within the pie charts indicate the NISP for each category.

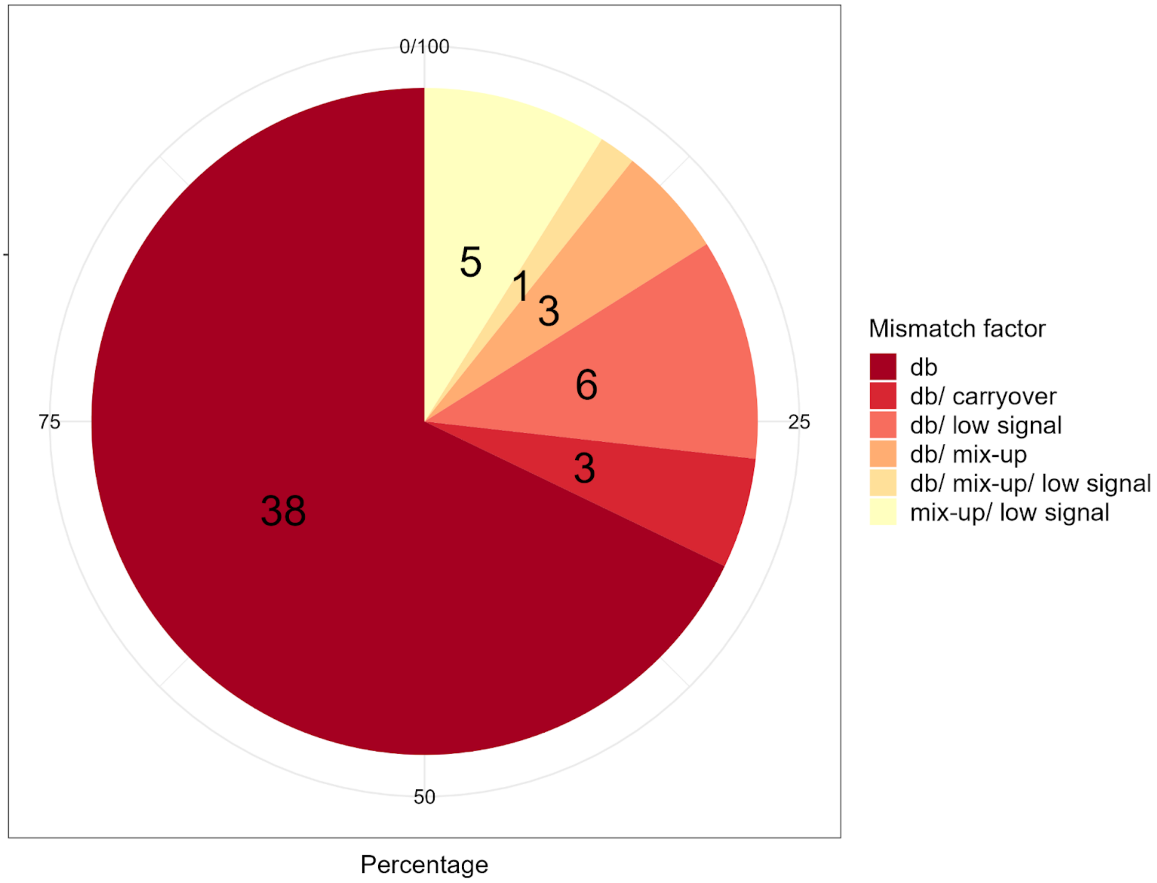

Figure S12. Pie chart of potentially incompatible taxonomic identifications and the potential reason for the non-matching taxonomic identifications for layers X (1932-1938 excavation), 9 and 8 (2016-2022 excavation).

Numbers within the pie charts indicate the NISP for each category. db stands for "database".

3. Zooarchaeology

The faunal reference collection stored at the Max Planck Institute for Evolutionary Anthropology (Leipzig) alongside reference atlases were used to assign fragments to species and skeletal elements, where possible[57,58]. The bone surfaces of all specimens were studied to locate and identify specific bone surface modifications (cut marks, impact points, percussion notches, carnivore tooth pits etc.) using a 20x magnification hand lens using an oblique light source following previous protocols[59–64].

For the purpose of this study, we focused on layers 9 and 8. Additionally, we included the overlying layer 7, to see what changed in terms of species composition and human modification of bones after the LRJ. For a detailed comparison of zooarchaeological, taphonomic and human behaviour for layers 12-7 the reader should refer to Smith et al.[33].

Excavations in 2016-2022 at Ranis have recovered a large quantity of piece plotted faunal material (>20 mm) from layers correlated with the LRJ (layers 9 and 8) plus the overlying layer 7 (n = 1,082). Further, a sub-sample of faunal material from the 1932-1938 excavation were also analysed (n = 1,194) from two squares where hominins were identified through ZooMS (Squares 51A and 164) or from archaeological layers correlated to the LRJ through the stratigraphy of the 2016-2022 excavations ("X" and "Graue Schicht"; see main text for correlation).

Fauna from 2016-2022 and 1932-1938 excavations from layers 9-7 included at least 17 taxa dominated by Cervidae (including *Rangifer tarandus* and *Cervus elaphus*) horse (*Equus ferus*) and *Bos/Bison* (Tab. S9, SI). A more diverse carnivore fauna was also identified from both 2016-2022 and 1932-1938 excavations dominated by cave bear (*Ursus spelaeus*) and Canidae (*Canis lupus, Vulpes lagopus, Vulpes vulpes*) alongside smaller quantities of felidae (*Panthera leo spelaeus*), cave hyaena (*Crocuta crocuta spelaea*) and wolverine (*Gulo gulo*). Overall, the taxa identified morphologically were consistent with those obtained through ZooMS analysis, though as noted there were some differences in abundance for specific species or family groups[33]. The faunal spectrum was characteristic of the biostratigraphic zone Mammal Neogene Quaternary (MNQ) 26[65] and was consistent between 2016-2022 and 1932-1938 excavations and correlates with ZooMS identifications.

Fauna from layers 9-7 contained taxa from the Mammuthus-Coelodonta Faunal Complex[66] including Elephantidae (most probably species *Mammuthus primigenius*), woolly rhinoceros (*Coelodonta antiquitatis*), reindeer (*Rangifer tarandus*), arctic fox (*Vulpes lagopus*) and wolverine (*Gulo gulo*). Taken together, these species were indicative of extremely cold climates and elements of this faunal complex recovered from layers 9-7 suggested accumulation and occupation during an extremely cold phase. However, the faunal assemblage from 2016-2022 and 1932-1938 excavations illustrated a mix of species adapted to both warmer and colder environments and associated shifts in local environments[24,59,67]. This was also consistent with sedimentological data indicating a cooling of climates within the LRJ layers and the layer above. The presence of open, more steppic environments associated with colder climates was also suggested by the presence of equidae (*Equus ferus*) and steppe bison (*Bison priscus*). Although there were some species identified that were more adapted to closed (*Bos primigenius* and canids) or mixed environments overall the pattern illustrates a more consistent cold signal. Further work on the micro-mammal fauna along with

stable isotopes from herbivore teeth would help to further clarify the climate and environment signal from the macro mammal assemblage[68]. Overall, the macro-mammal assemblage from Ranis illustrated that the Late Pleistocene climate around the site was considerably cooler than present day, potentially with a mix of environmental conditions at both a local and broader regional scale.

Overall, the faunal material recovered from layers 9-7 was well preserved with a low degree of sub-aerial weathering (>90% low weathering; Tab. S10, SI) and high degree of bone surface readability (>85% having high bone surface readability; Tab. S11, SI). This permitted the identification of both carnivore and anthropogenic bone surface modifications. Alongside the varied carnivore taxa identified within layers 9-7 (through both traditional comparative morphology and ZooMS analysis) there was a strong signal of carnivore activity (20-35% of bones modified) on the bones throughout all layers correlated to the LRJ (Tab. S12, SI). In contrast, anthropogenic modifications were recovered in lower proportions throughout all LRJ layers (1-5% of bones modified) with the highest proportion from layer 8. Modifications were recorded across large herbivore taxa including *Bos/Bison, Equus* and Cervidae including skinning, scraping and deliberate fracturing of bones to extract bone marrow. Skinning marks on carnivore species including cave bear (*Ursus spelaeus*), wolf (*Canis lupus*) and fox (*Vulpes vulpes*) could reflect exploitation of these species for furs, though other bone modifications could suggest an additional use for nutritional purposes that cannot be excluded especially in light of evidence from sites such as Bacho Kiro Cave[59].

No bone tools or personal ornaments were identified in either piece plotted material or from the smaller, screened fraction, from old or 2016-2022 excavations. This was in direct contrast to other *Homo sapiens* sites of similar age in Europe[24,59,69]. However, from layer X[15] a bone point and perforated ivory disc fragment were reported which unfortunately are now lost. This hints at the presence of worked bone items in the LRJ that appear visually similar to material from recently described tools and ornaments from Bacho Kiro Cave, which are of comparable age and associated with early *Homo sapiens* groups[59,69]. Overall, zooarchaeological and taphonomic analysis of both morphologically and ZooMS identified specimens suggested that large carnivores such as cave hyaena played the major role in bone accumulation. There was limited evidence for long-term human occupation at Ranis within the LRJ layers with current evidence suggesting short, ephemeral visits perhaps during time frames when the site was not being used by other large carnivores. Indeed, such short-term human occupation combined with intensive site use by large carnivores such as cave bear and cave hyaena appeared to be a feature of many LRJ sites[5,11,33,70].

Table S9. Analysed faunal remains from layers 9-7 at Ranis from 1932-1938 and 2016-2022 excavations.

This table includes all the identifications using traditional morphological comparisons and those identified through ZooMS. With ZooMS certain families or taxa are not distinguishable based on the current peptide marker system, which explains the combined categories such as Cervid/Saiga. %NISP = percentage of number of identified specimens.

	1932-1938 excavations		2016-2022 excavations					
**Taxon**	**Layer X**	**%NISP**	**Layer 9**	**%NISP**	**Layer 8**	**%NISP**	**Layer 7**	**%NISP**
Homo sp.	9	1.1	1	1.2	3	1.3	0	0.0
Aves sp.	9	1.1	0	0.0	3	1.3	6	4.7
Leporidae sp.	0	0.0	0	0.0	1	0.4	6	4.7
Felinae	0	0.0	0	0.0	1	0.4	0	0.0
Felinae/Ursidae	6	0.7	0	0.0	0	0.0	0	0.0
Crocuta spelaea	4	0.5	1	1.2	0	0.0	2	1.6
Hyaenidae/Felidae	1	0.1	0	0.0	0	0.0	0	0.0
Hyaenidae/Pantherinae	14	1.7	1	1.2	2	0.9	0	0.0
Canis lupus	6	0.7	3	3.7	0	0.0	1	0.8
Canidae sp.	2	0.2	0	0.0	0	0.0	2	1.6
Canidae (not *Vulpes vulpes*)	4	0.5	0	0.0	1	0.4	1	0.8
Vulpes vulpes	0	0.0	0	0.0	1	0.4	0	0.0
Vulpes lagopus	4	0.5	1	1.2	0	0.0	6	4.7
Gulo gulo	0	0.0	0	0.0	1	0.4	0	0.0
Ursus spelaeus	22	2.7	1	1.2	7	3.1	9	7.0
Ursidae sp.	226	28.2	22	27.2	55	24.6	9	7.0
Elephantidae sp.	16	2.0	7	8.6	3	1.3	1	0.8
Coelodonta antiquitatis	3	0.4	0	0.0	0	0.0	0	0.0
Rhinocerotidae sp.	29	3.6	5	6.2	28	12.5	3	2.3
Equus ferus	10	1.2	0	0.0	0	0.0	4	3.1
Equidae sp.	59	7.4	7	8.6	18	8.0	14	10.9
Cervus elaphus	5	0.6	0	0.0	1	0.4	1	0.8

Cervid/Saiga	8	1.0	5	6.2	9	4.0	1	0.8
Cervid/Saiga/*Capreolus*	27	3.4	0	0.0	1	0.4	0	0.0
Rangifer tarandus	241	30.0	13	16.0	67	29.9	27	20.9
Rangifer/Caprinae	1	0.1	1	1.2	1	0.4	0	0.0
Rangifer/Bovidae	6	0.7	0	0.0	0	0.0	0	0.0
Cervidae sp.	22	2.7	4	4.9	3	1.3	29	22.5
Bison priscus	1	0.1	1	1.2	0	0.0	0	0.0
Bos primigenius	2	0.2	0	0.0	0	0.0	1	0.8
Bos/Bison sp.	54	6.7	8	9.9	18	8.0	6	4.7
Bos/*Bison*/Muskox	4	0.5	0	0.0	0	0.0	0	0.0
Bovidae/Cervidae	5	0.6	0	0.0	0	0.0	0	0.0
Equidae/Cervidae/Saiga	2	0.2	0	0.0	0	0.0	0	0.0
**Total identified**	**802**	**100.0**	**81**	**100.0**	**224**	**100.0**	**129**	**100.0**
**Identification rate**	**67.2**		**70.4**		**91.8**		**17.8**	
Carnivora	34		0		1		0	
carnivore large	1		1		0		0	
carnivore medium	2		1		2		2	
carnivore small	0		0		0		1	
carnivore unknown	1		0		0		3	
ungulate large	19		1		1		17	
ungulate medium large	9		1		0		25	
ungulate small medium	0		0		0		8	
mammal unknown	284		29		14		536	

Indeterminate	28		0		0		1	
unidentified	0		0		0		1	
Empty	14		1		2		0	
**Total unidentified**	**392**		**34**		**20**		**594**	
**Number of specimens**	**1194**		**115**		**244**		**723**	

Table S10. Weathering stages (from Behrensmeyer[71]) for bone material from 1932-1938 and 2016-2022 excavations.

Weathering stage (Ws) 0 = unweathered while Ws 4 = heavily weathered; the bone material from both excavations illustrates that >85% of bone material is lightly weather WS 0-2 and appears consistent between the two excavations.

1932-1938 excavations												2016-2022 excavations											
**Layer**	**NSP**	**0**	**%**	**1**	**%**	**2**	**%**	**3**	**%**	**4**	**%**	**Layer**	**NSP**	**0**	**%**	**1**	**%**	**2**	**%**	**3**	**%**	**4**	**%**
												7	723	565	78.1	143	19.8	15	2.1	0	0.0	0	0.0
X	420	342	81.4	58	13.8	14	3.3	5	1.2	1	0.2	8	244	200	82.0	40	16.4	4	1.6	0	0.0	0	0.0
												9	115	91	79.1	18	15.7	4	3.5	2	1.7	0	0.0

Table S11. Bone surface readability for bone remains from 1932-1938 and 2016-2022 excavations.

This measure relates to proportion of bone surface renaming and readable (0% = no original surface remaining; 100% = all original surface remaining)[59]; all bone remains from layers 9-7 have >75% of original bone surfaces indicating a high level of preservation.

1932-1938 excavations										2016-2022 excavations									
**Layer**	**NSP**	**0 %**	**%**	**<50 %**	**%**	**>50 %**	**%**	**100 %**	**%**	**Layer**	**NSP**	**0 %**	**%**	**<50 %**	**%**	**>50 %**	**%**	**100 %**	**%**
										7	723	6	0.8	101	14.0	303	41.9	313	43.3
X	419	5	1.2	52	12.4	110	26.3	252	60.1	8	244	2	0.8	28	11.5	82	33.6	132	54.1
										9	115	0	0.0	22	19.1	43	37.4	50	43.5

Table S12. Faunal remains from 1932-1938 and 2016-2022 excavations with the number of specimens that record carnivore and human modification.

Carnivore modifications: tooth pits, tooth scratches, crenelation, digested pieces. Human modifications: chop marks, skinning, disarticulation cut marks, deliberate marrow fractures. For detailed discussion of human behaviour see Smith et al. 2023[33].

1932-1938 excavations						2016-2022 excavations					
**Layer**	**NSP**	**carnivore**	**%**	**human**	**%**	**Layer**	**NSP**	**carnivore**	**%**	**human**	**%**
						7	723	166	23.0	2	0.3
X	1189	74	6.2	22	1.9	8	244	46	18.9	9	3.7
						9	115	41	35.7	3	2.6

4. Radiocarbon dating

4.1. Bone pretreatment

All ^{14}C dated bones from the 2016-2022 collection (Tab. S13 in additional .xlsx file) were newly excavated and had no conservatives applied to their surfaces. The bones samples were pretreated with the collagen extraction protocol published in Fewlass et al. 2019[72], Talamo et al. 2021[73], including treatment with 0.1M NaOH to remove humic acid contamination and an ultrafiltration step (Sartorius VivaSpin Turbo 15) to concentrate the large molecular weight fraction of the extract (>30 kDa MWCO). To test the effect of these two purification steps, two bones were first pretreated with the standard protocol (A), and then again but omitting the NaOH and ultrafiltration steps (B; Tab. S14, SI). Both extracts were dated, and the results were in agreement between the different pretreatment protocols (statistically identical for 16/116-151564 and overlapping at 95.4% probability for 16/116-124430). This implied that the level of contamination of these two collagen extracts was low, and that the standard pretreatment protocol used was sufficiently removing any traces of exogenous carbon. This was further indicated by the elemental values of all extracts (Tab. S13, SI), which fall within the ranges of modern collagen samples[74–76], and the stratigraphic consistency of the dates (see below). The molecular dating of the human mtDNA provides additional independent support for the accuracy of the ^{14}C results. Furthermore, four direct ^{14}C dates from five *Homo sapiens* bones that originate either from the same or maternally related individuals based on their mtDNA are statistically indistinguishable (R10396, R10874, R10879 and 16/116-159327 were dated, whilst R10355 was not). When the four dates are combined in OxCal (Combine function), they give a combined age of 45,000 - 44,180 cal BP with an agreement index (Acomb) of 157.1% (X^2 test: df = 3, T = 0.864 (5% 7.8)). When taken together with the exceptionally high level of collagen preservation from all samples at the site (100% success rate for collagen extraction), we consider the ^{14}C data produced from these samples to be highly robust and reliable.

Table S14. Comparison of AMS dates for two bones prepared with two collagen extraction protocols.

	**Standard protocol (A)**					**Modified protocol (B)**					
**Sample ID**	**Weight (mg)**	**Coll yld (%)**	**AMS lab code**	**^{14}C BP ± 1sd**	**95.4% cal BP**	**Weight (mg)**	**Coll yld (%)**	**AMS lab code**	**^{14}C BP ± 1sd**	**95.4% cal BP**	**X^2 test (df=1)**
16/116-124430	414.8	8.4	ETH-111928	39,330 ± 280	43,060-42,480	305.2	13.0	ETH-111938	38,310 ± 250	42,600-42,160	T=7.4 (5% 3.8)
16/116-151564	361.1	8.5	ETH-111932	43,750 ± 470	47,300-45,160	338.3	10.6	ETH-111939	43,110 ± 430	46,300-44,730	T=1.0 (5% 3.8)

In contrast, two of the hominin bones identified from the 1932-1938 excavation (R10355 and R10318) showed signs of residue on their surfaces. Werner Hülle's notebooks[15] from the 1930s recorded four methods of conservation applied to bones excavated from the site, so that the bones from the 1930s collection were in five conditions:

1. Untreated
2. Conserved with bone collagen-based glue
3. Conserved by heating in paraffin (1934)
4. Conserved with Zaponlac (1935)
5. Conserved with Geißeltal-Lack

Unfortunately, it was not recorded on which bones these conservative treatments were applied. Therefore, the residue from R10355 was analysed with FTIR to determine its origin and was identified as paraffin (Fig. S13, SI). An excerpt from Werner Hülle's notebook from 1934 (p. 29)[15] supported this identification, as he recorded a new method for conserving moist bones by slowly heating them in paraffin, followed by heating to 100°C in air.

Given this evidence, the small size of both contaminated fragments (<1 g weight) and the high number of hominin remains recovered from the site, these two fragments were excluded from direct ^{14}C dating. Following mtDNA analysis, it was determined that R10355 may originate from the same individual or be maternally related to four other fragments with statistically indistinguishable dates, which therefore provide a reliable indicator for the age of this bone fragment. The other human remains from the 1930s collection showed no visible signs of conservatives and given their agreement in elemental values and dates with the un-treated hominin remains from the 2016-2022 excavation, we conclude that these were untreated.

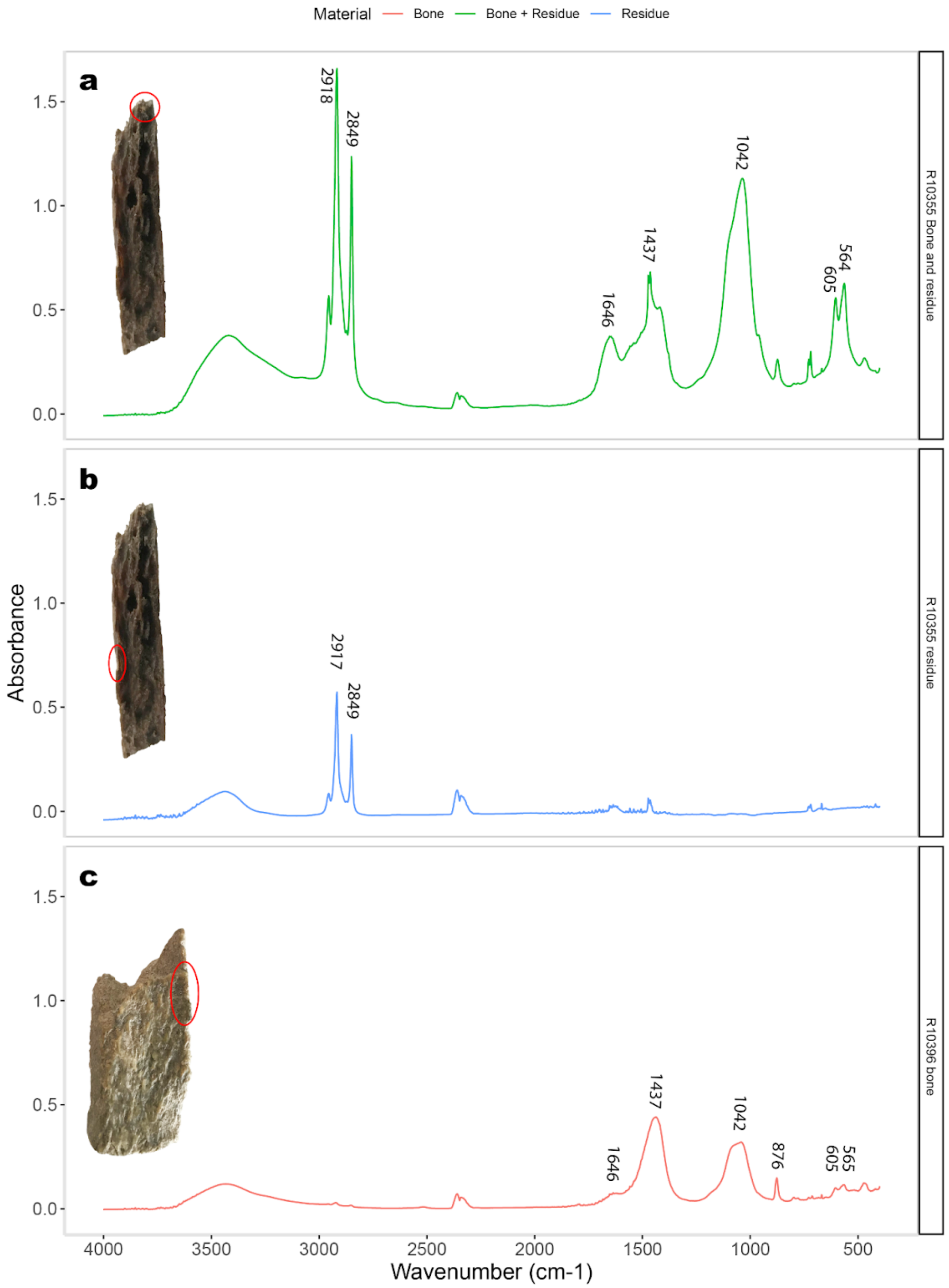

Figure S13. FTIR spectra of human bones R10355 and R10396.

a) Spectra obtained from R10355 showing characteristic bone peaks - 1646 cm^{-1}, 1437 cm^{-1}, 1042 cm^{-1} as well as the 605 cm^{-1} and 564 cm^{-1} doublet - and additional peaks at ~2918 cm^{-1} and ~2849 cm^{-1}. **b**) Spectra of the residue from the surface of R10355 showing only the additional peaks at ~2918 cm^{-1} and ~2849 cm^{-1}, which are consistent with paraffin[77]. **c**) Spectra from R10396 only showing peaks characteristic of bone mineral. The location of the sampled area is indicated by the red circle on the image on the left.

4.2. Bayesian modelling

Calibration and Bayesian modelling of the radiocarbon dates was carried out in OxCal v4.4.4[78] using the IntCal20 calibration curve[79]. We report all calibrated age estimates with 95.4% confidence intervals with ages rounded to the nearest 10 years. We first constructed a multi-phase Bayesian model for the layer 11-7 dates from the 2016-2022 excavation using the stratigraphic layers as prior information. Of the 30 dates obtained from the new excavation, only two of the charcoal dates (MAMS-48368 and MAMS-48369) were excluded from the model based on their small sample size and low C% following pretreatment. Two dates from the base of the sequence in layer 11 provided infinite ages (F14C < 2sd), which are included in the model with their F14C values, thus providing a minimum start date for layer 11. A 'General' outlier model was used with prior probabilities for each date set to 5%[80]. This formally assesses the posterior likelihood of each sample being an outlier and downweights outliers in the final model. Extended Data Fig. 3 (main text) and Tab. S15 (SI) show the results of the site modelling, with the individual agreement index (A) and the posterior outlier probability (O) of each date. Only one date (ETH-111935, bone 116-159318) from layer 9 was identified as an outlier, which overlaps with the dates from the overlying layer 8. Statistically we would expect 1 date in 20 to be an outlier so one outlier is expected for a dataset of this size (n = 28). The outlier was a horse bone with signs of human modifications on the surface, and the chemical data showed no sign of contamination which could lead to an inaccurate date. In the eastern part of square 1004/1000, where the bone was found in layer 9, layer 8 was only a few millimetres thick, and we cannot exclude that the bone was mixed into layer 9 by post-depositional processes (geological or animal). The model was run more than three times to ensure that the results were consistent. Our model agreement (Amodel) was 40.8. Generally, an overall model agreement index >60 indicates that the posterior distribution agrees with the prior distribution, and the model is considered acceptable, however when an Outlier Model is applied, the effect of outliers are down-weighted in the final model and this threshold criteria does not apply.

A second model was constructed, grouping the dates from layers 9 and 8 together in one LRJ phase (Tab. S16, SI). This model identified no outliers, thus resulting in a higher model agreement index (82.7) compared to the model with layers 9 and 8 grouped separately. This model provides further support for the close link between layers 9 and 8 and indicates that the single outlier in the first model does indeed originate from layer 8. However, the boundaries of the LRJ do not change significantly between the models (1st model: 47,500 - 43,260 cal BP; second 9+8 model: 47,620 - 43,350 cal BP) so we refer to the first model that includes all the stratigraphic information. Overall, the dating and outlier analysis indicate good stratigraphic integrity of layers 11-7.

Table S15. Modelled boundaries of ^{14}C dates included in the site chronological model shown in Extended Data Figure 3.

The directly dated hominin remains from the 2016-2022 excavation are highlighted in pink and asterisks (*) mark bones with anthropogenic surface modifications. Modelled boundaries are shown in bold. A indicates the Agreement indices where A > 60 indicates the sample is in agreement with the model. O shows the posterior outlier probability. One outlier (16/116-159318, ETH-111935) was identified (coloured red). The model agreement is 40.8, but as we applied a General Outlier Model, the effect of outliers are down-weighted in the final model.

Arch ID	AMS lab code	Unmodelled calibrated range (cal BP)				Modelled calibrated range (cal BP)				A (%)	O (%)
		68.3% probability		95.4% probability		68.3% probability		95.4% probability			
		from	to	from	to	from	to	from	to		
**End Layer 7**						**42240**	**39950**	**42340**	**39110**		
16/116-151382 *	ETH-121935	40900	40420	41050	40070	42610	40340	43730	40070	69	36
16/116-150212	MAMS-48367	42250	42000	42360	41850	42270	42010	42400	41820	102	3
16/116-151393	ETH-111930	43800	42880	44030	42810	43450	42870	44000	42790	108.3	3
16/116-150274	ETH-111929	42840	42570	42980	42450	42850	42560	43040	42420	101.8	3
16/116-124430	ETH-111928	42900	42610	43060	42480	42910	42610	43110	42460	102.2	2
16/116-124429	ETH-111927	42560	42340	42700	42250	42560	42340	42710	42240	103.5	1
16/116-150209 *	ETH-121936	44280	43400	44500	43130	43930	43170	44420	42980	96	4
**Transition Layer 8/Layer 7**						**45650**	**43350**	**45890**	**43260**		
16/116-159091 *	ETH-118367	44080	43190	44430	42930	46000	43760	46190	43540	52.7	33
16/116-159327	ETH-127620	45380	44040	45970	43200	45900	44390	46140	43980	96.6	3
16/116-159253	ETH-127619	46020	44580	47480	44100	46030	44940	46330	44370	117.4	2
16/116-159223 *	ETH-118368	46750	45350	47680	44850	46170	45400	46490	44890	121.7	3
16/116-159070 *	ETH-118366	46600	45360	47420	44870	46150	45420	46470	44940	122	3
16/116-151564	ETH-111932	46610	45600	47300	45160	46170	45550	46520	45180	116.8	3
16/116-150358 *	ETH-111931	46490	45500	47130	45080	46140	45530	46480	45130	119.1	3
16/116-159155	ETH-111933	47070	45970	47860	45630	46320	45730	46640	45360	86.5	5
16/116-159199	ETH-127621	47900	45700	49560	44850	46270	45460	46570	44850	97.7	4
**Transition Layer 9/Layer 8**						**46550**	**46010**	**46820**	**45770**		
16/116-159318 *	ETH-111935	44760	44260	45140	43920	46730	46140	47110	45900	5.4	100
16/116-159416	ETH-127622	47390	45330	48680	44640	46740	46160	47110	45910	135	2

16/116-159380 *	ETH-121939	47480	46140	48250	45790	46750	46170	47120	45950	120.8	2
16/116-159376 *	ETH-121938	46120	45180	46800	44810	46720	46150	47000	45880	46.4	4
16/116-159296	ETH-111934	47230	46060	48010	45780	46740	46170	47090	45950	127.5	2
**Transition Layer 10/Layer 9**						**46960**	**46270**	**47500**	**46030**		
16/116-159523	ETH-121941	46920	45750	47750	45350	47610	46590	48310	46210	64.2	7
16/116-159508	ETH-121940	54240	49600	...	48860	50750	48840	52000	48030	90.6	7
**Transition Layer 11/Layer 10**						**51920**	**49860**	**53790**	**48710**		
16/116-186405 *	ETH-121944	54400	49920	...	49270	52640	50560	54530	50060	113	3
16/116-186171 *	ETH-121943	51160	48610	54700	47870	52370	50470	54690	49600	67.8	6
16/116-159586 *	ETH-121942	54350	49810	...	49050	52620	50550	54530	50040	112	4
16/116-189239 *	ETH-127632	...	52670	...	54940	53510	51200	54760	50380	71.3	5
16/116-186285 *	ETH-127631	...	52590	...	52960	53470	51130	54690	50340	74.5	5
**Start Layer 11**						**54960**	**51850**	**55860**	**50530**		

Table S16. Modelled boundaries of ^{14}C dates in a site chronological model where layers 9 and 8 dates are modelled together in one LRJ phase.

The directly dated hominin remains from the 2016-2022 excavation are highlighted in pink and asterisks (*) mark bones with anthropogenic surface modifications. Modelled boundaries are shown in bold. A indicates the Agreement indices where A > 60 indicates the sample is in agreement with the model. O shows the posterior outlier probability. No outliers are identified, and the model agreement is 82.7, higher than the model above.

Arch ID	AMS lab code	Unmodelled calibrated range (cal BP)				Modelled calibrated range (cal BP)				A (%)	O (%)
		68.3% probability		95.4% probability		68.3% probability		95.4% probability			
		from	to	from	to	from	to	from	to		
**End Layer 7**						**40940**	**39830**	**42290**	**38880**		
16/116-151382 *	ETH-121935	40900	40420	41050	40070	41010	40490	43190	40020	88.3	16
16/116-150212	MAMS-48367	42250	42000	42360	41850	42260	42000	42370	41840	101	4
16/116-151393	ETH-111930	43800	42880	44030	42810	43450	42870	43950	42800	106.4	4
16/116-150274	ETH-111929	42840	42570	42980	42450	42850	42570	43000	42440	101	4
16/116-124430	ETH-111928	42900	42610	43060	42480	42910	42610	43080	42480	101.1	4
16/116-124429	ETH-111927	42560	42340	42700	42250	42560	42340	42710	42240	101.1	4
16/116-150209 *	ETH-121936	44280	43400	44500	43130	43880	43220	44240	43040	97.8	5
**Transition Layer 9+8/Layer 7**						**44370**	**43750**	**44600**	**43350**		
16/116-159091 *	ETH-118367	44080	43190	44430	42930	44540	43980	44980	43520	64.2	8
16/116-159327	ETH-127620	45380	44040	45970	43200	45300	44270	46040	43910	111.2	4
16/116-159253	ETH-127619	46020	44580	47480	44100	45920	44670	46710	44320	110	4
16/116-159223 *	ETH-118368	46750	45350	47680	44850	46490	45390	46910	44860	112.4	4
16/116-159070 *	ETH-118366	46600	45360	47420	44870	46390	45360	46850	44920	110.1	4
16/116-151564	ETH-111932	46610	45600	47300	45160	46450	45570	46870	45210	109.1	4
16/116-150358 *	ETH-111931	46490	45500	47130	45080	46360	45500	46820	45150	107.8	4
16/116-159155	ETH-111933	47070	45970	47860	45630	46690	45960	47080	45520	112.8	4
16/116-159199	ETH-127621	47900	45700	49560	44850	46730	45580	47150	44880	111.8	5
16/116-159318 *	ETH-111935	44760	44260	45140	43920	44780	44300	45130	44050	104.6	4

16/116-159416	ETH-127622	47390	45330	48680	44640	46630	45390	47040	44720	116	4
16/116-159380 *	ETH-121939	47480	46140	48250	45790	46740	46010	47210	45600	104.3	5
16/116-159376 *	ETH-121938	46120	45180	46800	44810	46090	45210	46650	44850	104.5	4
16/116-159296	ETH-111934	47230	46060	48010	45780	46710	46010	47150	45610	110.4	5
**Transition Layer 10/Layer 9+8**						**47180**	**46470**	**47620**	**46220**		
16/116-159523	ETH-121941	46920	45750	47750	45350	47730	46770	48230	46430	51.6	6
16/116-159508	ETH-121940	54240	49600	...	48860	50700	48850	51870	48170	90.5	5
**Transition Layer 11/Layer 10**						**51900**	**49840**	**53840**	**48720**		
16/116-186405 *	ETH-121944	54400	49920	...	49270	52680	50570	54570	50100	111	4
16/116-186171 *	ETH-121943	51160	48610	54700	47870	52380	50480	54750	49590	66.8	5
16/116-159586 *	ETH-121942	54350	49810	...	49050	52650	50540	54580	50080	109.9	5
16/116-189239 *	ETH-127632	...	52670	...	54940	53610	51280	54800	50490	73.8	5
16/116-186285 *	ETH-127631	...	52590	...	52960	53600	51220	54760	50450	76.9	5
**Start Layer 11**						**55000**	**52110**	**56010**	**50570**		

Table S17. Radiocarbon and thermoluminescence (TL) (Brno-Bohunice 2) dates from contemporary sites and human remains included in Fig. 2.

This table includes the TL dates and uncalibrated radiocarbon dates reported in the original publications. Radiocarbon dates have been re-calibrated with IntCal20 for Fig. 2, with Kernel Density Estimations for each site shown in Fig. 2a and calibrated ranges for each individual shown in Fig. 2b.

**Fig. 2a**					
**Context/Site**	**Material (method)**	**Lab code**	**AMS/TL date**	**Error**	**Ref. #**
N1-I, Bacho Kiro Cave, Bulgaria	Bone (^{14}C)	ETH-86769	39,750	380	81
N1-I, Bacho Kiro Cave, Bulgaria	Bone (^{14}C)	ETH-86783	40,340	280	81
N1-I, Bacho Kiro Cave, Bulgaria	Bone (^{14}C)	ETH-86771	40,600	420	81
N1-I, Bacho Kiro Cave, Bulgaria	Bone (^{14}C)	ETH-86780	40,760	290	81
N1-I, Bacho Kiro Cave, Bulgaria	Bone (^{14}C)	ETH-71316	40,790	250	81
N1-I, Bacho Kiro Cave, Bulgaria	Bone (^{14}C)	ETH-71318	41,080	260	81
N1-I, Bacho Kiro Cave, Bulgaria	Bone (^{14}C)	ETH-71327	41,170	260	81
N1-I, Bacho Kiro Cave, Bulgaria	Bone (^{14}C)	ETH-71331	41,200	260	81
N1-I, Bacho Kiro Cave, Bulgaria	Bone (^{14}C)	ETH-71322/MAMS-29483	41220	210	81
N1-I, Bacho Kiro Cave, Bulgaria	Bone (^{14}C)	ETH-71315/MAMS-28677	41,310	180	81
N1-I, Bacho Kiro Cave, Bulgaria	Bone (^{14}C)	ETH-71320	41,450	270	81
N1-I, Bacho Kiro Cave, Bulgaria	Bone (^{14}C)	ETH-71325	41,480	270	81
N1-I, Bacho Kiro Cave, Bulgaria	Bone (^{14}C)	ETH-86784	41,660	320	81
N1-I, Bacho Kiro Cave, Bulgaria	Bone (^{14}C)	ETH-71329	41,730	280	81
N1-I, Bacho Kiro Cave, Bulgaria	Bone (^{14}C)	ETH-71314	41,770	210	81
N1-I, Bacho Kiro Cave, Bulgaria	Bone (^{14}C)	ETH-71324/MAMS-28681	41,820	250	81
N1-I, Bacho Kiro Cave, Bulgaria	Bone (^{14}C)	ETH-86770	41,850	480	81
N1-I, Bacho Kiro Cave, Bulgaria	Bone (^{14}C)	ETH-71328	41,850	280	81
N1-I, Bacho Kiro Cave, Bulgaria	Bone (^{14}C)	ETH-71323/MAMS-28680	41,950	250	81
N1-I, Bacho Kiro Cave, Bulgaria	Bone (^{14}C)	ETH-71330	42,270	300	81
N1-I, Bacho Kiro Cave, Bulgaria	Bone (^{14}C)	ETH-86772	42,450	510	81
N1-J Upper, Bacho Kiro Cave, Bulgaria	Bone (^{14}C)	ETH-93194	42,670	370	81
N1-J Upper, Bacho Kiro Cave, Bulgaria	Bone (^{14}C)	ETH-86789	42,900	370	81
Brno-Bohunice 2, Czech Republic	Flint (TL)	EVA-LUM 06/13	48,900	4700	82
Brno-Bohunice 2, Czech Republic	Flint (TL)	EVA-LUM 06/12	46,400	5300	82

Brno-Bohunice 2, Czech Republic	Flint (TL)	EVA-LUM 06/11	50,200	5100	[82]
Brno-Bohunice 2, Czech Republic	Flint (TL)	EVA-LUM 06/10	49,800	5500	[82]
Brno-Bohunice 2, Czech Republic	Flint (TL)	EVA-LUM 06/9	49,400	4900	[82]
Brno-Bohunice 2, Czech Republic	Flint (TL)	EVA-LUM 06/8	46,700	4900	[82]
Brno-Bohunice 2, Czech Republic	Flint (TL)	EVA-LUM 06/7	44,200	4900	[82]
Brno-Bohunice 2, Czech Republic	Flint (TL)	EVA-LUM 06/6	43,400	4300	[82]
Brno-Bohunice 2, Czech Republic	Flint (TL)	EVA-LUM 06/5	51,100	5000	[82]
Brno-Bohunice 2, Czech Republic	Flint (TL)	EVA-LUM 06/4	48,100	5400	[82]
Brno-Bohunice 2, Czech Republic	Flint (TL)	EVA-LUM 06/3	46,400	5100	[82]
LRJ Bone bed, Glaston Grange Farm, UK	Bone (^{14}C)	OxA-22149	38,400	900	[10]
LRJ Bone bed, Glaston Grange Farm, UK	Bone (^{14}C)	OxA-21311 & OxA-21312	37,984	265	[10]
LRJ Bone bed, Glaston Grange Farm, UK	Bone (^{14}C)	OxA-21310	38,800	390	[10]
LRJ Bone bed, Glaston Grange Farm, UK	Bone (^{14}C)	OxA-21309	38,120	360	[10]
LRJ Bone bed, Glaston Grange Farm, UK	Bone (^{14}C)	OxA-21308	35,610	300	[10]
Líšeň/Podolí I, Czechia	Charcoal (^{14}C)	Poz-87130	39,400	1000	[23]
Líšeň/Podolí I, Czechia	Charcoal (^{14}C)	Poz-87129	35,800	600	[23]
Líšeň/Podolí I, Czechia	Charcoal (^{14}C)	Poz-87128	36,900	600	[23]
Líšeň/Podolí I, Czechia	Charcoal (^{14}C)	Poz-87126	37,100	800	[23]
Líšeň/Podolí I, Czechia	Charcoal (^{14}C)	Poz-87125	37,900	700	[23]
Líšeň/Podolí I, Czechia	Charcoal (^{14}C)	Poz-37344	38,400	700	[23]
Želešice III, Czechia	Charcoal (^{14}C)	OxA-27342	41,300	700	[83]
Želešice III, Czechia	Charcoal (^{14}C)	Poz-51617	42,500	1500	[83]
Želešice III, Czechia	Charcoal (^{14}C)	Poz-37821	37,770	800	[83]
Layer D, Kozarnia Cave, Poland	Ursus tooth (^{14}C)	Poz-99816	39,000	1000	[84]
Layer D, Kozarnia Cave, Poland	Ursus tooth (^{14}C)	Poz-99773	37,650	900	[84]
Schmähingen, Germany	Bone (^{14}C)	Erl-20425	42,660	1380	[6]
Schmähingen, Germany	Bone (^{14}C)	Erl-20424	36,950	640	[6]
Schmähingen, Germany	Bone (^{14}C)	Erl-20423	35,940	625	[6]
**Fig. 2b**					
**Human specimen**	**Species**	**AMS lab code**	**AMS date (BP)**	**error**	**Ref. #**
Bacho Kiro Cave AA7-738, Bulgaria	*Homo sapiens*	ETH-86769	39,750	380	[24]; [81]

Bacho Kiro Cave BB7-240, Bulgaria	*Homo sapiens*	ETH-86770	41,850	480	[24]; [81]
Bacho Kiro Cave CC7-2289, Bulgaria	*Homo sapiens*	ETH-86771	40,600	420	[24]; [81]
Bacho Kiro Cave CC7-335, Bulgaria	*Homo sapiens*	ETH-86772	42,450	510	[24]; [81]
Ust'Ishim femur, Siberia	*Homo sapiens*	OxA-25516/OxA-30190 (R_combine)	41,400	953	[85]
Grotte du Renne AR-14, France	Neanderthal	MAMS-25149	36,840	660	[86]
St-Césaire SP28, France	Neanderthal	OxA-18099	36,200	750	[87]
La Ferrassie 8 (ZooMS LF-86), France	Neanderthal	ETH-99102	36,170	220	[88]
Fonds-de-Forêt femur, Belgium	Neanderthal	OxA-38322/OxA-X-2767-13 (R_Combine)	39,080	679	[89]
Engis 2, Belgium	Neanderthal	OxA-38394	39,900	1700	[89]
Spy 94a, Belgium	Neanderthal	OxA-X-2762-21/OxA-X-2778-15 (R_Combine)	40,567	1422	[89]
Spy 589a, Belgium	Neanderthal	OxA-38790	41,700	2300	[89]
Spy 737a, Belgium	Neanderthal	OxA-X-2762-6	41,600	2400	[89]

4.3. OxCal code for site model shown in Extended Data Fig. 3 and Tab. S15.

```
Plot()
 {
  Outlier_Model("General",T(5),U(0,4),"t");
  Sequence("Ranis")
  {
   Boundary("Start Layer 11");
   Phase("Layer 11")
   {
    R_F14C("ETH-127631", 0.00104, 0.00054)
    {
     Outlier(0.05);
     color="green";
    };
    R_F14C("ETH-127632", 0.00086, 0.00054)
    {
     Outlier(0.05);
     color="green";
    };
    R_Date("ETH-121942", 48380, 940)
    {
     Outlier(0.05);
     color="green";
    };
    R_Date("ETH-121943", 47260, 830)
    {
     Outlier(0.05);
     color="green";
    };
    R_Date("ETH-121944", 48480, 950)
    {
     Outlier(0.05);
     color="green";
    };
   };
   Boundary("Transition Layer 11/Layer 10");
   Phase("Layer 10 (sterile)")
   {
    R_Date("ETH-121940", 48240, 920)
    {
     Outlier(0.05);
    };
```

```
 R_Date("ETH-121941", 44000, 550)
 {
  Outlier(0.05);
 };
};
Boundary("Transition Layer 10/Layer 9");
Phase("Layer 9")
{
 R_Date("ETH-111934", 44410, 510)
 {
  Outlier(0.05);
 };
 R_Date("ETH-121938", 43320, 510)
 {
  Outlier(0.05);
  color="green";
 };
 R_Date("ETH-121939", 44560, 590)
 {
  Outlier(0.05);
  color="green";
 };
 R_Date("ETH-127622", 43888, 1037)
 {
  color="pink";
  Outlier(0.05);
 };
 R_Date("ETH-111935", 41620, 360)
 {
  Outlier(0.05);
  color="green";
 };
};
Boundary("Transition Layer 9/Layer 8");
Phase("Layer 8")
{
 R_Date("ETH-127621", 44334, 1093)
 {
  color="pink";
  Outlier(0.05);
 };
 R_Date("ETH-111933", 44260, 500)
 {
  Outlier(0.05);
```

```
  };
  R_Date("ETH-111931", 43670, 460)
  {
   Outlier(0.05);
   color="green";
  };
  R_Date("ETH-111932", 43750, 470)
  {
   Outlier(0.05);
  };
  R_Date("ETH-118366", 43580, 610)
  {
   Outlier(0.05);
   color="green";
  };
  R_Date("ETH-118368", 43640, 700)
  {
   Outlier(0.05);
   color="green";
  };
  R_Date("ETH-127619", 42733, 914)
  {
   color="pink";
   Outlier(0.05);
  };
  R_Date("ETH-127620", 41804, 802)
  {
   color="pink";
   Outlier(0.05);
  };
  R_Date("ETH-118367", 40560, 480)
  {
   Outlier(0.05);
   color="green";
  };
 };
 Boundary("Transition Layer 8/Layer 7");
 Phase("Layer 7")
 {
  R_Date("ETH-121936", 40850, 380)
  {
   Outlier(0.05);
   color="green";
  };
```

```
  R_Date("ETH-111927", 38540, 250)
  {
   Outlier(0.05);
  };
  R_Date("ETH-111928", 39330, 280)
  {
   Outlier(0.05);
  };
  R_Date("ETH-111929", 39200, 270)
  {
   Outlier(0.05);
  };
  R_Date("ETH-111930", 40090, 300)
  {
   Outlier(0.05);
  };
  R_Date("MAMS-48367", 37570, 270)
  {
   Outlier(0.05);
  };
  R_Date("ETH-121935", 35440, 200)
  {
   Outlier(0.05);
   color="green";
  };
 };
 Boundary("End Layer 7");
};
};
```

5. Ancient DNA analysis

5.1. Lineage determination

A total of fifteen libraries from eleven hominin skeletal fragments and three libraries from negative controls were evaluated for the presence of ancient human DNA. Between 4,415 and 175,668 unique reads mapping to the human mtDNA reference genome were found in the fifteen hominin libraries, compared to between 11 and 101 in the accompanying negative controls. All fifteen hominin libraries also contained elevated C-to-T substitutions on their 5' and 3' ends, confirming the presence of authentic ancient DNA. None of the negative controls contained evidence for ancient human DNA (Tab. S25 and Fig. S14-S24, SI). Multiple libraries generated from the same skeletal element (four libraries from R10396 and three from R10355) were merged and reprocessed as described in the methods section, resulting in one dataset per specimen. The estimated modern human contamination varied from 0.1% to 25.77% based on deamination patterns[90] (Tab. S25).

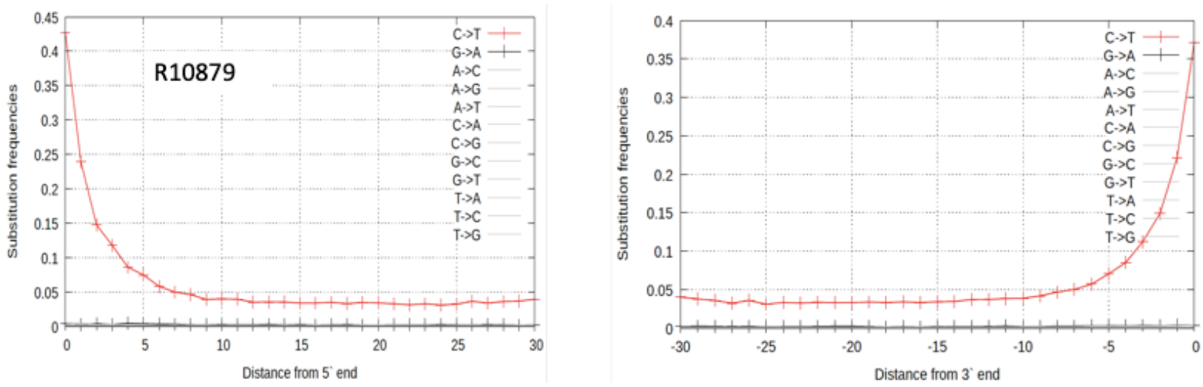

Figure S14. The frequency of base substitutions relative to the human mtDNA reference genome for filtered DNA fragments (length > 34bp, mapping quality >= 25) recovered from R10879.

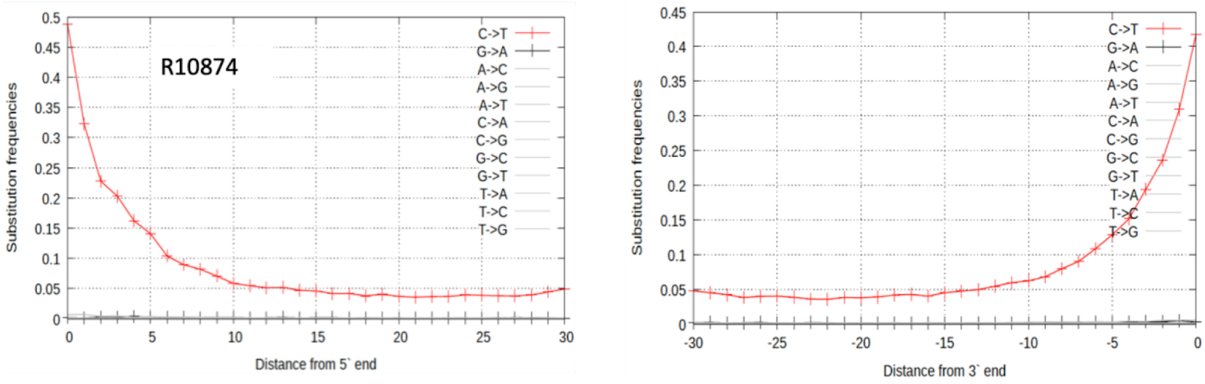

Figure S15. The frequency of base substitutions relative to the human mtDNA reference genome for filtered DNA fragments (length > 34bp, mapping quality >= 25) recovered from R10874.

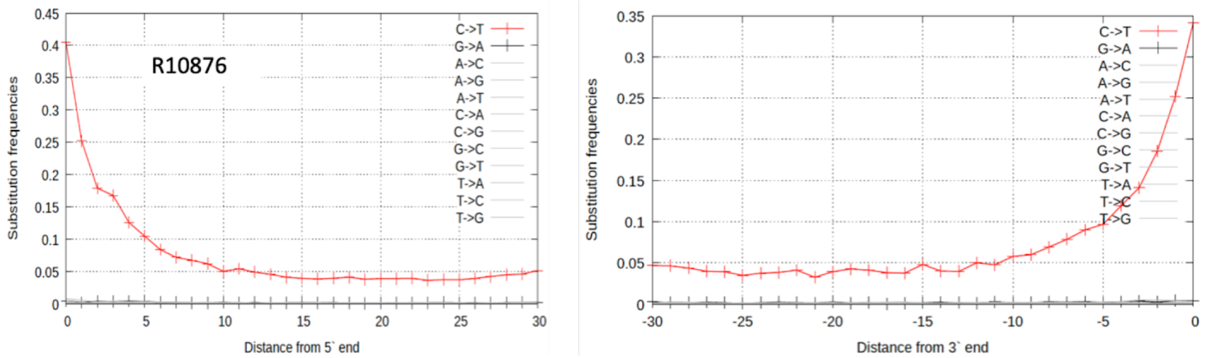

Figure S16. The frequency of base substitutions relative to the human mtDNA reference genome for filtered DNA fragments (length > 34bp, mapping quality >= 25) recovered from R10876.

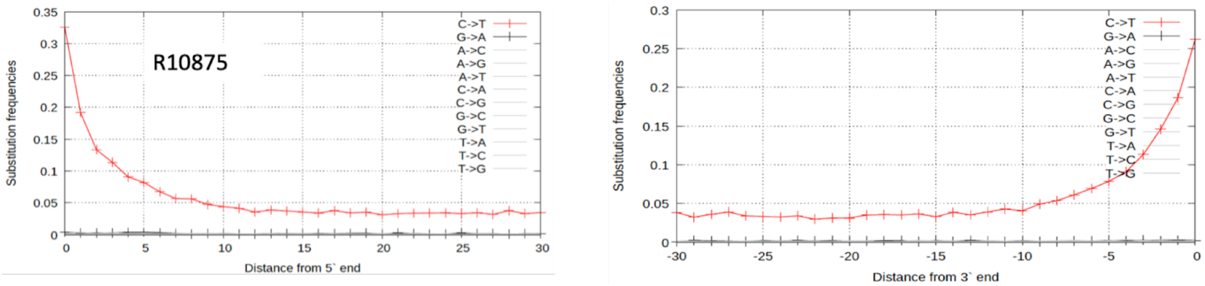

Figure S17. The frequency of base substitutions relative to the human mtDNA reference genome for filtered DNA fragments (length > 34bp, mapping quality >= 25) recovered from R10875.

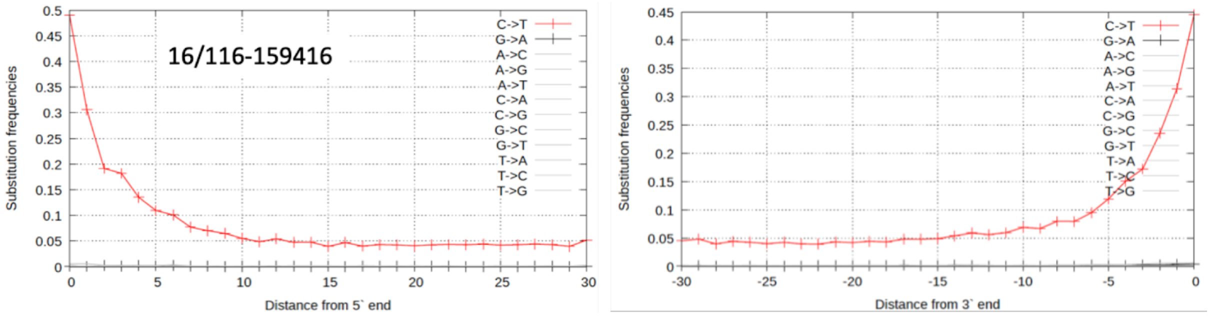

Figure S18. The frequency of base substitutions relative to the human mtDNA reference genome for filtered DNA fragments (length > 34bp, mapping quality >= 25) recovered from 16/116-159416.

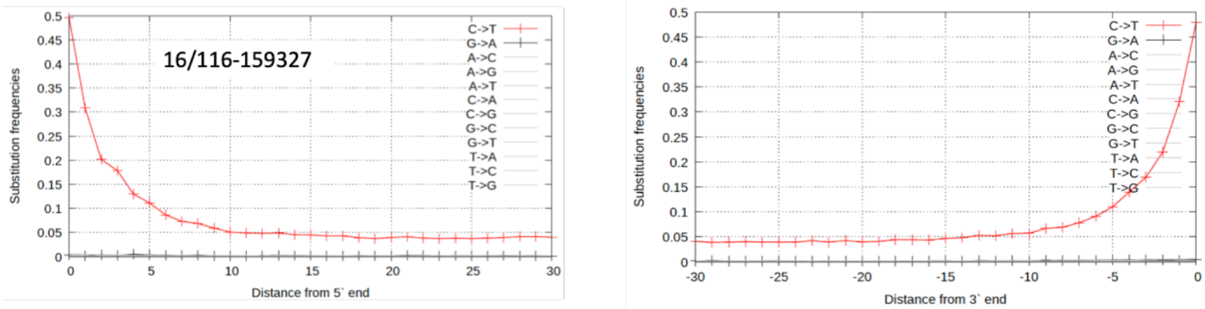

Figure S19. The frequency of base substitutions relative to the human mtDNA reference genome for filtered DNA fragments (length > 34bp, mapping quality >= 25) recovered from 16/116-159327.

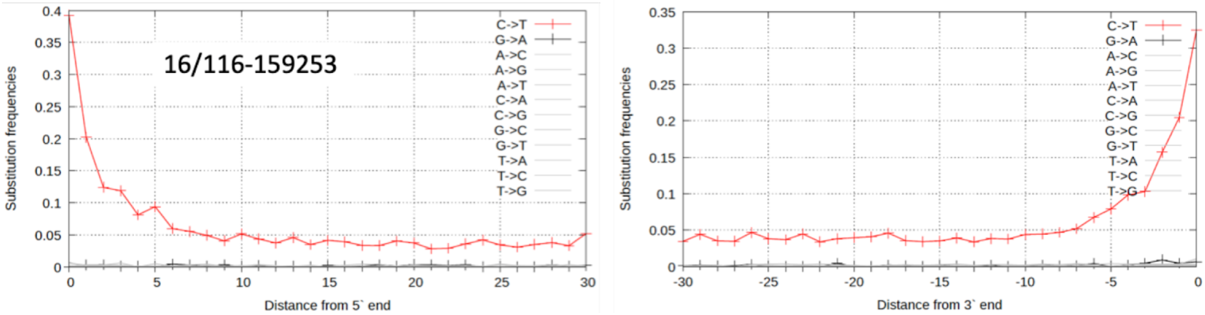

Figure S20. The frequency of base substitutions relative to the human mtDNA reference genome for filtered DNA fragments (length > 34bp, mapping quality >= 25) recovered from 16/116-159253.

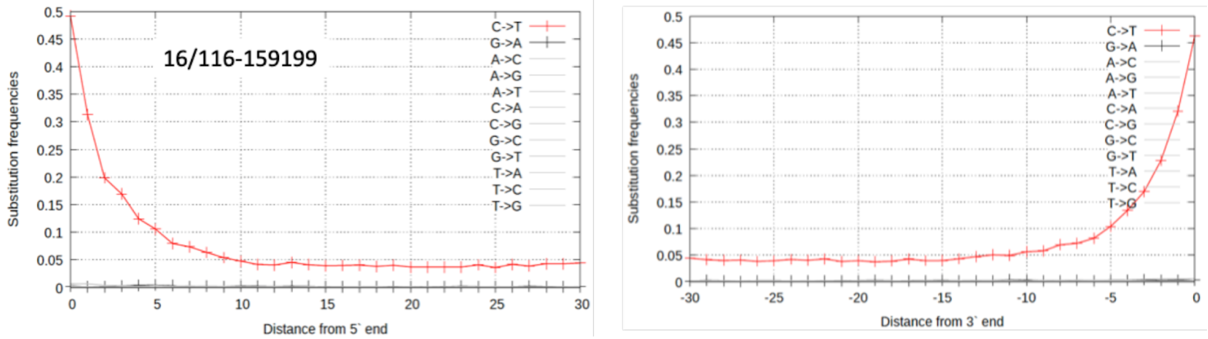

Figure S21. The frequency of base substitutions relative to the human mtDNA reference genome for filtered DNA fragments (length > 34bp, mapping quality >= 25) recovered from 16/116-159199.

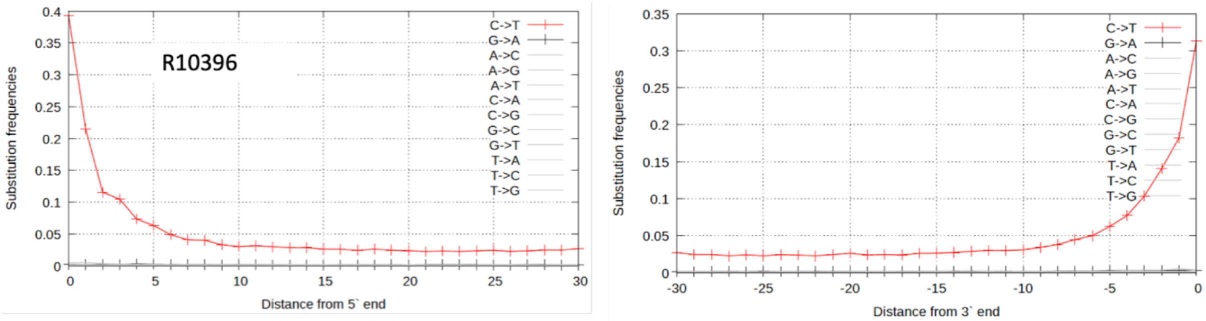

Figure S22. The frequency of base substitutions relative to the human mtDNA reference genome for filtered DNA fragments (length > 34bp, mapping quality >= 25) recovered from R10396.

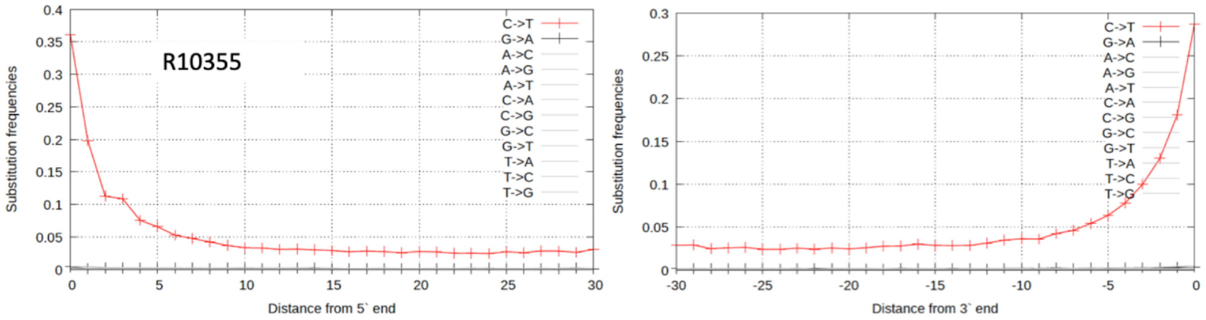

Figure S23: The frequency of base substitutions relative to the human mtDNA reference genome for filtered DNA fragments (length > 34bp, mapping quality >= 25) recovered from R10355.

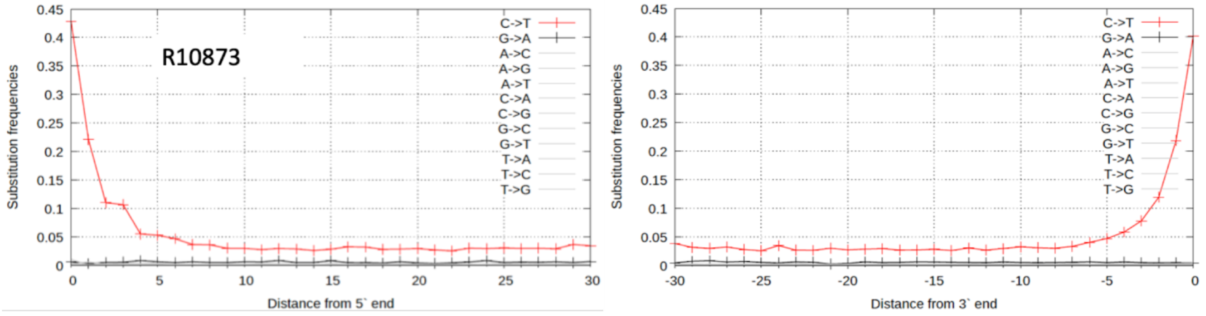

Figure S24. The frequency of base substitutions relative to the human mtDNA reference genome for filtered DNA fragments (length > 34bp, mapping quality >= 25) recovered from R10873.

We used previously determined lineage informative sites or diagnostic positions to determine the support for the presence of *Homo sapiens*, Neanderthal, or Denisovan mtDNA fragments among the recovered data from the Ranis specimens. These positions were determined using a chimpanzee mtDNA as an outgroup and requiring 311 human mtDNA genomes from across the world to share the same state[91]. We then counted the number of sequences overlapping these positions that shared the human state. In order to minimise the impact of deamination, lineage support was calculated by ignoring C-to-T substitutions on the forward strands and G-to-A substitutions on the reverse strands in the terminal 7 positions.

When calculating the human lineage support using all fragments and when limiting only to putatively deaminated fragments, the support was over 95% for the human state (Tab. S18, SI).

Table S18. The estimated percent support for the human lineage using previously published diagnostic positions.

The number of diagnostic positions covered by reads and total number of positions covered are shown in parentheses.

ID	% Human support (all fragments)	% Human support (deaminated fragments)
16/116-159416	98.59 (976/990)	97.73 (344/352)
16/116-159327	98.58 (2,699/2,738)	98.67 (964/977)
16/116-159253	97.55 (199/204)	100 (50/50)
16/116-159199	97.57 (1,365/1,399)	96.5 (469/486)
R10875	97.13 (1,183/1,218)	97.4 (300/308)
R10876	98.57 (967/981)	98.56 (274/278)
R10874	98.89 (2,595/2,624)	98.91 (906/916)
R10879	98.77 (6,120/6,196)	98.36 (1,680/1,708)
R10396	99.18 (7,786/7,850)	98.61 (1,838/1,864)
R10355	98.71 (3,378/3,422)	98.54 (741/752)
R10873	95.98 (980/1021)	96.52 (277/287)

5.2. Reconstruction of mtDNA genomes

The DNA recovered from ten of the eleven fragments contained at least 76-fold coverage of mtDNA genome when using all fragments and at least 20-fold coverage when restricting to putatively deaminated fragments, enabling the reconstruction of almost complete mtDNA genomes. A consensus base was called if at least five fragments overlapped the position and there was at least 80% support among them. To overcome drops in coverage around the mtDNA start and end, reads were also re-mapped to a version of the rCRS where positions 0-1000bp were copied over to the end and then lifted back to the original rCRS coordinates. Reconstruction of mtDNA genomes was limited to putatively deaminated fragments when contamination among all fragments was estimated to be greater than 5% (for the libraries of the specimens R10396, R10355, R10875, R10876, R10879). Finally, T's on the forward strands and A's on the reverse strands in the terminal seven positions were changed to an "N" to limit the impact of deamination in mtDNA reconstruction. We decided to perform this masking for the terminal seven positions in order to ensure deamination substitutions would be expected to be less than 10% based on the substitution plots (Fig. S14-24, SI).

All reconstructed mtDNA genomes contained an unresolved position in the C-stretch regions (positions 303-315). Interestingly, five of the mtDNA genomes also shared an unresolved call at position 16,093 due to low support (58.65 - 78.12% for the C allele, Tab.

S19, SI). If the correct call at this position were a C, deamination beyond the masked seven terminal positions could decrease this support. Therefore, we repeated consensus calling at this position by masking all T's on forward strands and A's on reverse strands, which resulted in an increase of support to between 91.41 and 100% (Tab. S19, SI). This position is also a known heteroplasmic hotspot, in particular when the predominant base is a C as may be the case in this scenario[92–94]. No evidence of cross-contamination due to index swapping was detected (see methods). Moreover, these libraries were produced in two different laboratories and sequenced in three different batches across three years. However, we cannot exclude the possibility of spurious low-level contamination from laboratory or non-laboratory sources impacting this position. Therefore, we decided to use a more conservative approach and keep the call as unresolved ("N") at this position for these five mtDNA genomes.

Among the newly reconstructed mtDNA genomes presented here, four that had less than five missing positions (R10396, R10879, R10874 and 16/116-159327) had no pairwise differences among them (Fig. S25, SI). This is consistent with these four skeletal fragments either coming from the same individual or from individuals that are part of the same maternal lineage. In addition, R10355 had no differences with these four mtDNA genomes and could therefore also be from the same individual or the same maternal lineage. This final association is more tentative due to the 13 missing positions in R10355's mtDNA genome and that it has no differences with other mtDNA genomes. R10875, R10876, and 16/116-159199 also had no observed differences among them, but this may be driven by the relatively higher number of missing positions in the first two mtDNA genomes (40 and 26, respectively). It should also be noted that like all the Ranis mtDNA genomes, the Zlatý kůň mtDNA genome is also missing one position in the C-stretch region. Haplogroups of the reconstructed mtDNA genomes were then determined using HaploGrep2 (v2.4.0)[95]. Nine of the mtDNA genomes were determined to belong to the haplogroup N and one (16/116-159199) was identified as belonging to the haplogroup R.

Table S19. Support for consensus base calling at position 16,093 of the human mtDNA genome (rCRS) for ten of the Ranis datasets.

Sample information in the first eight columns is from Table S25. Masking refers to not calling T's on forward strands or A's on reverse strands for the selected position. L.L.B. - Location of library build; Lej.- Leipzig; Est. Cont. - estimated contamination based on AuthentiCT; M.P. - total number of missing positions (positions without a consensus base called); Sup. - Support for the dominant base

ID	L.L.B	Lab batch	Seq. batch (year)	Est. Con. (%)	Data	~ mtDNA cov.	M.P.	Position 16,093 (Ref: T)					
								Masking (7 terminal positions)			Masking (all positions)		
								C.B.	Cov.	Sup. (%)	C.B.	Cov.	Sup. (%)
16/116-159199	Lej.	1	1 (2023)	2.4	all	112	1	t	103	98.06	t	58	96.55
16/116-159416	Lej.	1	1 (2023)	4.8	all	78	4	n	41	78.05	c	35	91.43
R10876	Jena	2	2 (2022)	8.0	deam	22	26	n	13	61.54	c	8	100
R10873	Jena	2	4 (2022)	2.9	all	81	3	c	78	94.87	c	75	98.67
R10875	Jena	2	2 (2022)	7.1	deam	23.1	40	c	18	100	c	18	100
R10874	Jena	2	2 (2022)	0.1	all	198	1	c	140	89.29	c	128	97.66
16/116-159327	Lej.	1	1 (2023)	3.7	all	220	1	c	186	80.11	c	163	91.41
R10396	Lej.	3	3 (2021)	24.0	deam	142	4	n	133	58.65	c	82	95.12
R10879	Jena	2	2 (2022)	5.29	deam	135	2	n	128	78.12	c	108	92.59
R10355	Lej.	3	3 (2021)	23.7	deam	61	13	n	52	73.08	c	41	92.68

	# missing positions	Fumane 2	Zlatý Kůň	R10875	**R10396***	**R10355***	**16/116-159327***	**R10874***	R10879*	16/116-159199	16/116-159416	R10876
Fumane 2	0											
Zlatý Kůň	1	9										
R10875	40	7	1									
**R10396***	4	8	1	2								
**R10355***	13	6	0	0	**0**							
**16/116-159327***	1	9	1	2	**0**	**0**						
**R10874***	1	9	1	2	**0**	**0**	**0**					
**R10879***	2	9	1	2	**0**	**0**	**0**	**0**				
16/116-159199	1	2	6	5	6	4	7	7	6			
16/116-159416	4	5	1	0	2	0	2	2	2	3		
R10876	26	7	1	0	2	0	2	2	2	4	0	
R10873	3	8	0	1	1	0	1	1	1	6	1	6

Figure S25. **The pairwise differences among the ten new mtDNA genomes and the previously published mtDNA genomes from the Zlatý kůň and Fumane 2 individuals.** The first column denotes the number of missing positions in the reconstructed mtDNA genome. Those with no differences among them are highlighted in bold and annotated with an asterisk. Zlatý kůň and Fumane 2 are depicted here as we determined that they were the previously published mtDNA genomes with the least number of pairwise differences to these new mtDNA genomes.

5.3. Tree building and branch shortening estimates

All ten newly reconstructed human mtDNA genomes were realigned to the rCRS with 54 modern human, 19 ancient human, and two Neanderthal mtDNA genomes using MAFFT (v7.453)[96]. BEAST2 v.2.6.6[97] was then used for tree building and estimating the dates of the ten new mtDNA genomes. All present-day modern humans were set at a time of present-day (date=0). For the previously published ancient modern humans and Neanderthals with direct radiocarbon dates, the dates were calibrated with IntCal20[79] in OxCal4.4[78] (Tab. S20, SI). For Zlatý Kůň, Fumane 2 and the newly reconstructed mtDNA genomes, the ages were restricted to a range of 0 to 60,000 years. The substitution model Tamura Nei 1993[98] was used, which has previously been shown to be the best model for ancient and archaic humans[99] as well as a mean mutation rate of 2.53×10^{-8} with a normal distribution and sigma of 1.0×10^{-10} [100].

A path sampling approach from the MODEL_SELECTION[101] package was used to determine the best fitting clock and tree model by comparing the resulting marginal log likelihoods. For this test, we used 40 path steps with chain lengths of 25,000,000 and parameter alpha of 0.3. A pre-burn of 75,000 iterations and entire chain length burn in of 80% was used. Both models with a Bayesian skyline tree model fit better than those with a constant population based on comparisons of their marginal log likelihoods (Bayes factors 8.2 to 13.0, Tab. S21, SI). As there was no significant difference between the Bayesian skyline models with a relaxed log normal clock or strict clock (Bayes factor 1.26, Tab. S21, SI), the simpler model (a strict clock with a Bayesian skyline tree model) was selected.

Three Markov chain Monte Carlo (MCMC) runs of 75,000,000 iterations with 10,000,000 pre-burn iterations were run on the selected model while sampling every 2,000 trees. The resulting log and tree files were combined using Logcombiner2 from BEAST2 and the program tree annotator was used to create a single summary tree. Tracer v.1.7.1 from the BEAST2 package was used to estimate and visualise the resulting tip dates from the

combined log files for all mtDNA genomes without radiocarbon dates (Tab. S22 and Fig. S26, SI). The resulting 95% highest posterior densities (HPDs) overlapped with each other and the estimated molecular date for Zlatý Kůň with the mean ages ranging from 40,918 to 49,105 years before present. FigTreev.1.4.4 was then used to visualise the resulting tree, which showed that nine of the new mtDNA genomes clustered with Zlatý Kůň, while one (16/116-159199) clustered with Fumane 2, Tianyuan, and one of the Bacho Kiro Cave individuals (CC7_2289; Fig. S27, SI and Fig. 3, main text).

Table S20. List of previously published radiocarbon dates and mtDNA genomes for ancient modern humans used for molecular dating with BEAST.

IntCal20[79] and OxCal 4.4[78] were used to re-calibrate the published radiocarbon dates.

Individual	Country	Radiocarbon AMS lab code	Raw Radiocarbon Date (BP)	Date Type	Raw Radiocarbon Date Reference	Radiocarbon Date (cal BP, 95.4% confidence interval)	Reference for mtDNA
Ust'-Ishim	Russia	OxA-25516; OxA-30190	41,400±950	Direct-UF	Fu et al., 2014[85]	42,900 - 45,930	Fu et al., 2014[85]
Boshan 11	China	MAMS-13530	7,368+-34	Direct	Fu et al., 2013[102]	8,030 - 8,320	Fu et al., 2013[100]
Loschbour	Luxembour g	OxA-7338	7,205+-50	Direct	Higham et al., 2007[103]	7,930 - 8,170	Fu et al., 2013[100]
Tianyuan	China	BA-03222	34,430±510	?	Fu et al., 2013[102]	38,070 - 40,850	Fu et al., 2013[102]
Kostenki 14	Russia	OxA-X-2395-15	33,250±500	HYP	Marmon et al., 2012[104]	36,670 - 39,380	Krause et al., 2010[105]
Iceman	Italy	OxA-3371–6; OxA-3419-21	4,540+-55	Direct	Kutschera et al.,1994[106]	4,970 - 5,450	Ermini et al., 2008[107]
Saqqaq	Greenland	OxA-20656	4,044 ± 31	Direct	Rasmussen et al., 2010[108]	4,410 - 4,790	Gilbert et al., 2008[109]
Oberkassel 998	Germany	OxA-4790	11,570+-100	Direct	Hedges et al., 1998[110]	13,180 - 13,730	Fu et al., 2013[100]
Dolní Věstonice 14	Czechia	Aix-12028	26,760±100	Direct-UF	Fewlass et al., 2019[111]	30,860 - 31,150	Fu et al., 2013[100]
Dolní Věstonice 13	Czechia	Aix-12027	27,040±100	Direct-UF	Fewlass et al., 2019[111]	31,030 - 31,240	Fu et al., 2013[100]
Bacho Kiro F6_620	Bulgaria	ETH-86773; Aix-12025	31660±140	Direct-UF	Fewlass et al., 2020[81]	42,580 - 43,930	Hublin et al., 2020[24]
Bacho Kiro BB7_240	Bulgaria	ETH-86770	41,850±480	Direct-UF	Fewlass et al., 2020[81]	43,940 - 45,550	Hublin et al., 2020[24]
Bacho Kiro CC7_335	Bulgaria	ETH-86772	42,450±510	Direct-UF	Fewlass et al., 2020[81]	44,420 - 45,930	Hublin et al., 2020[24]
Bacho Kiro CC7_2289	Bulgaria	ETH-86771	40,600±420	Direct-UF	Fewlass et al., 2020[81]	42,990 - 44,400	Hublin et al., 2020[24]
Bacho Kiro 1653	Bulgaria	ETH-86768; Aix-12024	30,570±120	Direct-UF	Fewlass et al., 2020[81]	34,610 - 35,290	Hublin et al., 2020[24]
Oase 1	Romania	GrA-22810; OxA-11711	34,950±900	Direct; Direct-UF	Trinkaus et al., 2003[112]	37,950 - 41,860	Fu et al., 2015[113]
Fumane 2	Italy	NA	NA	NA	NA	NA	Benazzi et al., 2015[114]
Salkhit	Mongolia	OxA-X-2717-25	30,430 ± 300	HYP	Devièse et al., 2019[115]	34,330 - 35,410	Devièse et al., 2019[115]

Vindija 33.19	Croatia	Ox-X-2717-11	44300±1200	HYP	Devièse et al., 2017[116]	44,690 - 49,930	Green et al., 2008[117]
Spy 94a	Belgium	OxA-X-2762-21	41,500 ± 1,800	HYP	Devièse et al., 2021[89]	42,330 - 49,660	Hajdinjak et al., 2019[118]

Table S21. The marginal log likelihoods for different clock and tree models.

Clock Model	Tree Model	Marginal log likelihood
Strict	Constant	-31,454.7
	Bayesian Skyline	-31,427.6
Relaxed log normal	Constant	-31,446.4
	Bayesian Skyline	-31,424.7

Table S22. The estimated tip dates of newly reconstructed mtDNA genomes and the previously published Zlatý kůň and Fumane 2 mtDNA genomes.
The asterisk (*) denotes those with no pairwise differences among their mtDNA genomes.

ID	Mean tip date (ka)	95% HPD Interval (ka)	ESS
Fumane 2	36,772	26,759 - 45,727	4,514
Zlatý kůň	45,461	36,457 - 54,081	655
R10875	48,300	40,441 - 56,130	732
R10876	48,643	40,694 - 56,608	753
**R10874***	41,310	29,358 - 51,010	698
**R10879***	41,318	29,379 - 51,057	694
**R10396***	41,335	29,270 - 50,888	681
**R10355***	45,121	34,092 - 55,468	992
16/116-159199	40,918	32,625 - 48,124	4,679
**16/116-159327***	41,317	29,212 - 50,864	671
16/116-159416	49,105	40,461 - 57,361	1,283
R10873	45,467	36,464 - 54,043	664

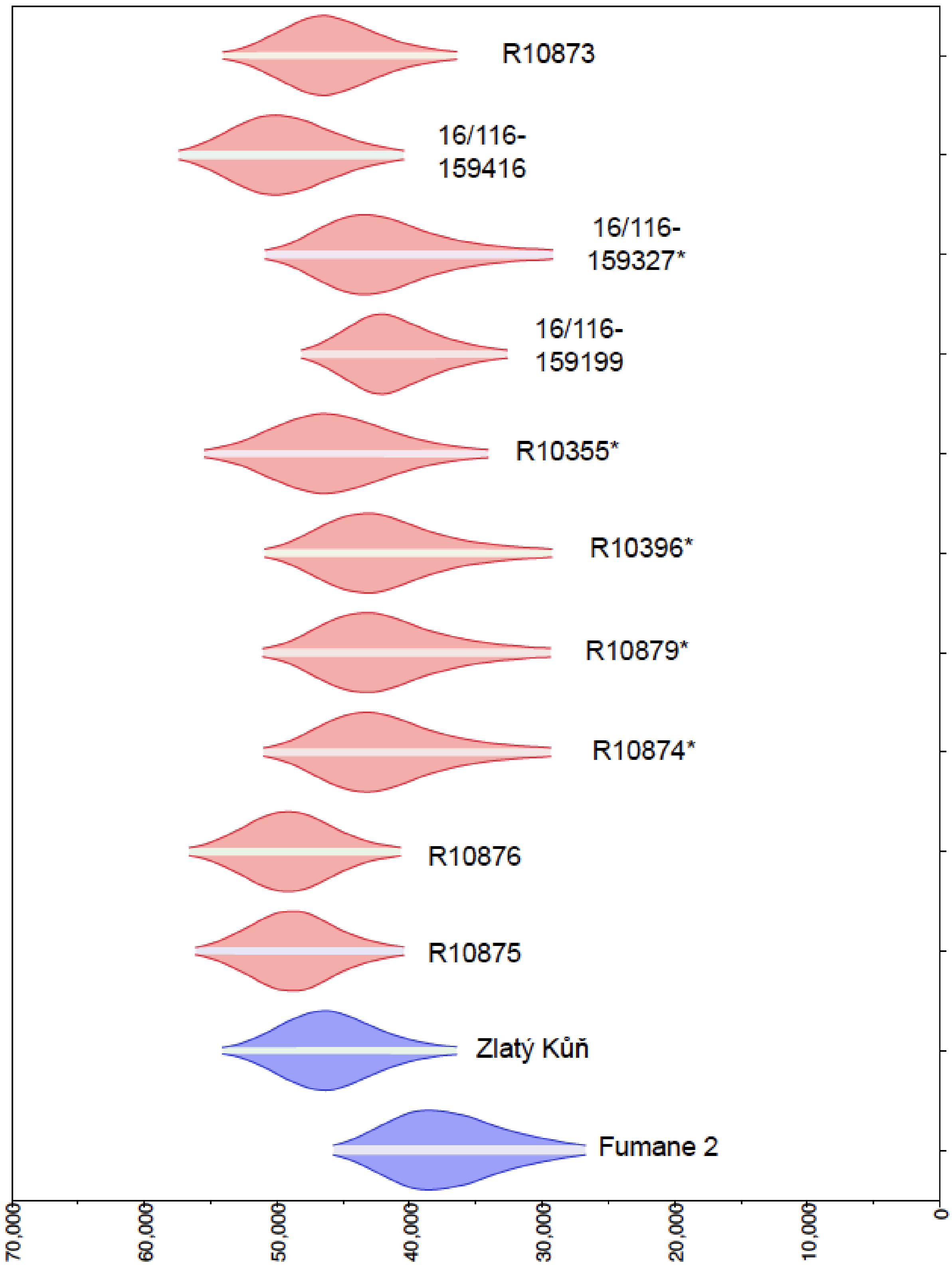

Figure S26. The estimated 95% HPD of molecular dates from BEAST2 visualised with Tracerv.1.7.1.

The asterisk (*) denotes mtDNA genomes with no pairwise differences. Dates for mtDNA genomes from this paper are coloured in red and those for previously published mtDNA genomes are coloured in blue.

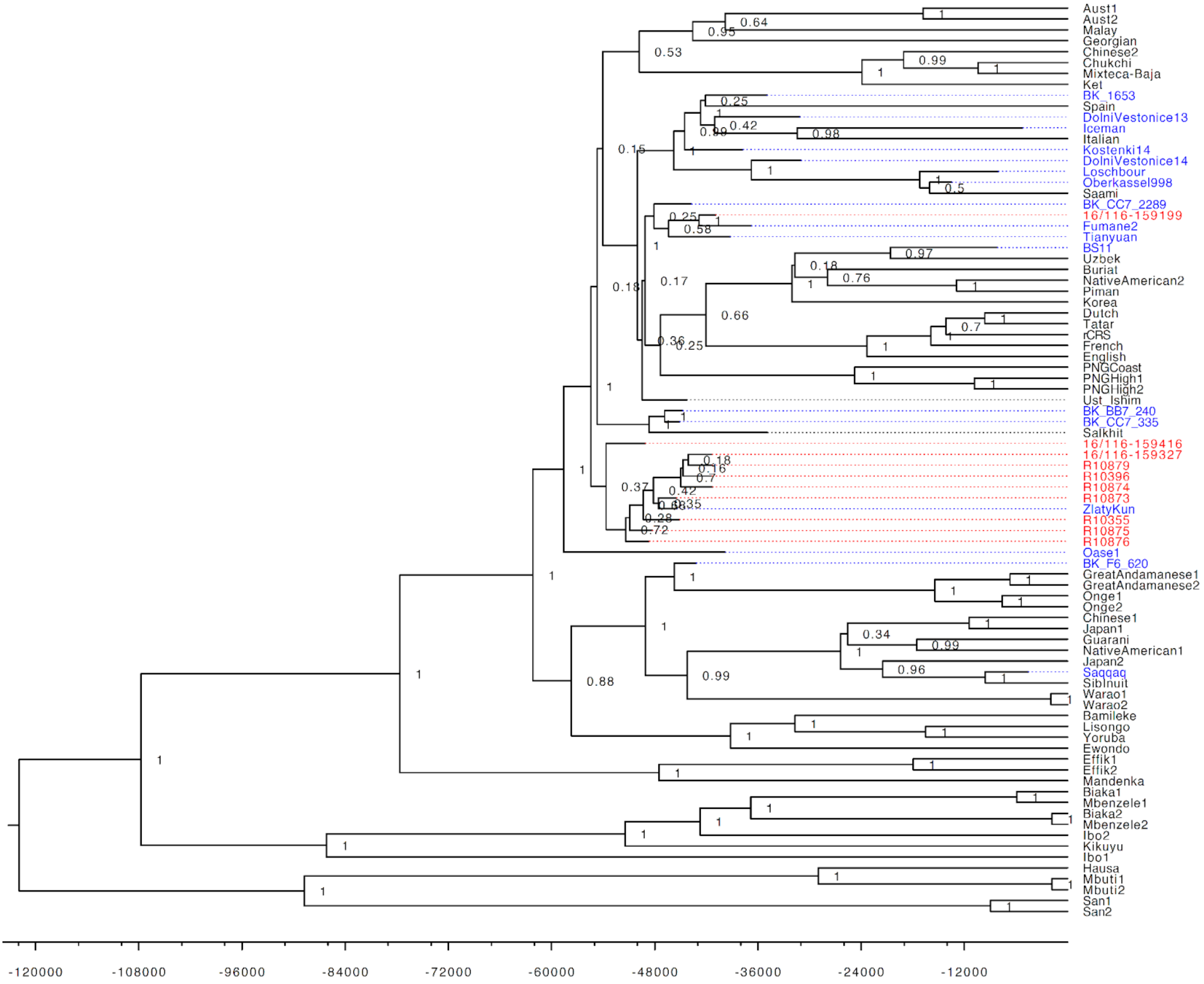

Figure S27. Tree constructed using BEAST2 of a selection of ancient and modern human mtDNA genomes.

The two Neanderthal mtDNA genomes used to root the tree are not shown. Branch tips in red represent new mtDNA genomes from this paper and those in blue represent previously published ancient modern humans. Nodes are labelled with the posterior and the x-axis shows the number of years in the past.

6. Additional files provided as supplementary material

Supplementary Table S13: Sample pretreatment information and radiocarbon dates from material excavated between 2016-2022 from layers 11 - 7 at Ranis.

Supplementary Table S23: Proteomic taxonomic identification of all bones specimens recovered in Ranis during the 2016-2022 excavation.

Supplementary Table S24: Proteomic taxonomic identification of all bones specimens recovered in Ranis during the 1932-1938 excavation.

Supplementary Table S25: Summary of sequencing data of libraries evaluated for human mitochondrial DNA content

Supplementary Table S26: Provenance information and radiocarbon dating of human remains from Ranis.

7. References

1. Desbrosse, R. & Kozłowski, J. K. *Hommes et climats à l'âge du mammouth: le paléolithique supérieur d'Eurasie centrale*. (Masson, 1988).
2. Flas, D. La transition du Paléolithique moyen au supérieur dans la plaine septentrionale de l'Europe. *Anthropologica & Praehistorica; 119* (2008).
3. Flas, D. La transition du Paléolithique moyen au supérieur dans la plaine septentrionale de l'Europe. Les problématiques du Lincombien-Ranisien-Jerzmanowicien. https://orbi.uliege.be/bitstream/2268/107122/1/Th%C3%A8se%20D.%20Flas%20vol.1%20texte.pdf (2006).
4. Jöris, O., Neruda, P., Wiśniewski, A. & Weiss, M. The Late and Final Middle Palaeolithic of Central Europe and Its Contributions to the Formation of the Regional Upper Palaeolithic: a Review and a Synthesis. *Journal of Paleolithic Archaeology* **5**, 17 (2022).
5. Flas, D. The Middle to Upper Paleolithic transition in Northern Europe: the Lincombian-Ranisian-Jerzmanowician and the issue of acculturation of the last Neanderthals. *World Archaeol.* **43**, 605–627 (2011).
6. Uthmeier, T., Hetzel, E. & Heißig, K. Neandertaler im spätesten Mittelpaläolithikum Bayerns? Die Jerzmanovice-Spitzen aus der Kirchberghöhle bei Schmähingen im Nördlinger Ries. *Bericht Der Bayerischen Bodendenkmalpflege* (2018).
7. Demidenko, Y. E. & Škrdla, P. Lincombian-Ranisian-Jerzmanowician Industry and South Moravian Sites: a Homo sapiens Late Initial Upper Paleolithic with Bohunician Industrial Generic Roots in Europe. *J. Paleolit. Archaeol.* **6**, 17 (2023).
8. Wiśniewski, A. *et al.* Lincombian-Ranisian-Jerzmanowician points were used primarily as hunting weapons: morphological and functional analysis of points from Nietoperzowa Cave, southern Poland. *Archaeol. Anthropol. Sci.* **14**, 90 (2022).
9. Jacobi, R., Debenham, N. & Catt, J. A Collection of Early Upper Palaeolithic Artefacts from Beedings, near Pulborough, West Sussex, and the Context of Similar Finds from the British Isles. *Proceedings of the Prehistoric Society* **73**, 229–326 (2007).

10. Cooper, L. P. *et al.* An Early Upper Palaeolithic Open-air Station and Mid-Devensian Hyaena Den at Grange Farm, Glaston, Rutland, UK. *Proceedings of the Prehistoric Society* **78**, 73–93 (2012).
11. Hussain, S. T., Weiss, M. & Kellberg Nielsen, T. Being-with other predators: Cultural negotiations of Neanderthal-carnivore relationships in Late Pleistocene Europe. *J. Anthropol. Archaeol.* **66**, 101409 (2022).
12. Chmielewski, W. Civilisation de Jerzmanowice. (Zakład Narodowy im. Ossolińskich, 1961)
13. Flas, D. Jerzmanowice points from Spy and the issue of the Lincombian-Ranisian-Jerzmanowician. in *Spy cave. 125 years of multidisciplinary research at the Betche aux Rotches (Jemeppe-sur-Sambre, Province of Namur, Belgium), Volume 1* (eds. Rougier, H. & Semal, P.) 217-230 (Anthropologica et Præhistorica 123/2012, 2013).
14. Pope, M., Dinnis, R., Milks, A., Toms, P. & Wells, C. A Middle Palaeolithic to Early Upper Palaeolithic succession from an open air site at Beedings, West Sussex. *Quat. Int.* **316**, 14–26 (2013).
15. Hülle, W. *Die Ilsenhöhle unter Burg Ranis, Thüringen: eine paläolithische Jägerstation*. (Verlag Gustav Fischer, 1977).
16. Škrdla, P. Middle to Upper Paleolithic transition in Moravia: New sites, new dates, new ideas. *Quat. Int.* **450**, 116–125 (2017).
17. Mester, Z. What about the Szeletian leaf point as fossile directeur? *Študijné zvesti Archeologického ústavu SAV* **Suppl**, 49–62 (2021).
18. Prošek, F. Szeletien na Slovensku. *Slovenská archeológia* **1**, 133–194 (1953).
19. Bohmers, A. Die Höhlen von Mauern. Teil I. Kulturgeschichte der Altsteinzeitlichen Besiedlung. *Palaeohistoria* **1**, 3–58 (1951).
20. Bosinski, G. *Die mittelpaläolithischen Funde im westlichen Mitteleuropa* (Böhlau, 1967).
21. Weber, T. Some remarks on transportation ways represented in the inventory Ranis 2 of the cave Ilsenhöhle, Ranis, Thuringia, GDR. in *Feuilles de pierre: les industries à pointes foliacées du paléolithique supérieur européen.* Krakow 1989, *Colloque* 239–246 (ERAUL 42, 1990).
22. Aldhouse-Green, S. *Paviland Cave and the'Red Lady': a definitive report.* (Western Acad &

Specialist Press Ltd, 2000).

23. Škrdla, P. *Moravia at the onset of the Upper Paleolithic.* (The Dolní Věstonice Studies 23, 2017).
24. Hublin, J.-J. *et al.* Initial Upper Palaeolithic Homo sapiens from Bacho Kiro Cave, Bulgaria. *Nature* **581**, 299–302 (2020).
25. Prüfer, K. *et al.* A genome sequence from a modern human skull over 45,000 years old from Zlatý kůň in Czechia. *Nat Ecol Evol* **5**, 820–825 (2021).
26. QGIS Development Team. *QGIS* Geographic Information System. http://qgis.osgeo.org. (Open Source Geospatial Foundation Project, 2023).
27. Reuter, H. I., Nelson, A. & Jarvis, A. An evaluation of void-filling interpolation methods for SRTM data. *Int. J. Geogr. Inf. Sci.* **21**, 983–1008 (2007).
28. Affinity – professional creative software. *Affinity* https://affinity.serif.com/en-gb/.
29. Bolus, M. Settlement Analysis of Sites of the Blattspitzen Complex in Central Europe. *In: Conard (Hrsg.), Settlement Dynamics of the Middle Paleolithic and Middle Stone Age II. Tübingen: Kerns Verlag.* 201–226. (2004).
30. Bosinski, G. *Die mittelpaläolithischen Funde im westlichen Mitteleuropa*. (Fundamenta, 1963).
31. Richter, J. The role of leaf points in the Late Middle Palaeolithic of Germany. *Praehistoria* **9-10**, 99–113 (2008-2009).
32. Zotz, L. F. *Das Paläolithikum in den Weinberghöhlen bei Mauern*. (L. Röhrscheid, 1955).
33. Smith, G. M. *et al.* The ecology, subsistence and diet of ca. 45,000-year-old Homo sapiens at Ranis Ilsenhöhle, Germany. *Nature Ecology and Evolution* (2024).
34. Stoops, G. *Guidelines for analysis and description of soil and regolith thin sections. SSSA Inc.: Madison WI, USA, 184 p*. (2003).
35. Stoops, G. *Guidelines for Analysis and Description of Soil and Regolith Thin Sections*. (John Wiley & Sons, 2021).
36. Nicosia, C. & Stoops, G. *Archaeological Soil and Sediment Micromorphology*. (John Wiley & Sons, 2017).
37. Blume, H.-P. *et al. Scheffer/Schachtschabel Soil Science*. (Springer Berlin Heidelberg).

38. Howard, P. J. A. & Howard, D. M. Use of organic carbon and loss-on-ignition to estimate soil organic matter in different soil types and horizons. *Biol. Fertil. Soils* **9**, 306–310 (1990).

39. Rasmussen, S. O. *et al.* A stratigraphic framework for abrupt climatic changes during the Last Glacial period based on three synchronized Greenland ice-core records: refining and extending the INTIMATE event stratigraphy. *Quat. Sci. Rev.* **106**, 14–28 (2014).

40. Lucke, B. & Schmidt, U. Grain size analysis of calcareous soils and sediments: intermethod comparison with and without calcium carbonate removal. *Soils and sediments as archives of landscape change: Geoarchaeology and landscape change in the subtropics and tropics. Selbstverlag der Fränkischen Geographischen Gesellschaft in Kommission bei Palm & Enke, Erlangen* 83–98 (2015).

41. Herrmann, N., Stadtmann, R., Meister, J. & Kirchner, A. Importance of substrate genesis for Mediterranean soil evolution – An example from a limestone hillslope in the Estremadura (W-Portugal). *Catena* **218**, 106566 (2022).

42. Jahn, R., Blume, H. P., Asio, V. B., Spaargaren, O. & Schad, P. *Guidelines for soil description, 4th edition*. - (FAO, 2006).

43. Buckley, M., Collins, M., Thomas-Oates, J. & Wilson, J. C. Species identification by analysis of bone collagen using matrix-assisted laser desorption/ionisation time-of-flight mass spectrometry. *Rapid Communications in Mass Spectrometry* vol. 23 3843–3854 Preprint at https://doi.org/10.1002/rcm.4316 (2009).

44. Rüther, P. L. *et al.* SPIN enables high throughput species identification of archaeological bone by proteomics. *Nat. Commun.* **13**, 1–14 (2022).

45. Bruderer, R. *et al.* Optimization of Experimental Parameters in Data-Independent Mass Spectrometry Significantly Increases Depth and Reproducibility of Results. *Mol. Cell. Proteomics* **16**, 2296–2309 (2017).

46. Bekker-Jensen, D. B. *et al.* Rapid and site-specific deep phosphoproteome profiling by data-independent acquisition without the need for spectral libraries. *Nat. Commun.* **11**, 787 (2020).

47. Batth, T. S., Francavilla, C. & Olsen, J. V. Off-line high-pH reversed-phase fractionation for

in-depth phosphoproteomics. *J. Proteome Res.* **13**, 6176–6186 (2014).

48. Ramsøe, A. *et al.* DeamiDATE 1.0: Site-specific deamidation as a tool to assess authenticity of members of ancient proteomes. *J. Archaeol. Sci.* **115**, 105080 (2020).
49. van Doorn, N. L., Wilson, J., Hollund, H., Soressi, M. & Collins, M. J. Site-specific deamidation of glutamine: a new marker of bone collagen deterioration. *Rapid Commun. Mass Spectrom.* **26**, 2319–2327 (2012).
50. Hill, R. C. *et al.* Preserved Proteins from Extinct Bison latifrons Identified by Tandem Mass Spectrometry; Hydroxylysine Glycosides are a Common Feature of Ancient Collagen*. *Mol. Cell. Proteomics* **14**, 1946–1958 (2015).
51. Orlando, L. *et al.* Recalibrating Equus evolution using the genome sequence of an early Middle Pleistocene horse. *Nature* **499**, 74–78 (2013).
52. Welker, F. *et al.* Ancient proteins resolve the evolutionary history of Darwin's South American ungulates. *Nature* **522**, 81–84 (2015).
53. Welker, F. *et al.* Variations in glutamine deamidation for a Châtelperronian bone assemblage as measured by peptide mass fingerprinting of collagen. *STAR: Science & Technology of Archaeological Research* **3**, 15–27 (2017).
54. Wilson, J., van Doorn, N. L. & Collins, M. J. Assessing the extent of bone degradation using glutamine deamidation in collagen. *Anal. Chem.* **84**, 9041–9048 (2012).
55. Ramsøe, A. *et al.* Assessing the degradation of ancient milk proteins through site-specific deamidation patterns. *Sci. Rep.* **11**, 7795 (2021).
56. Robinson, N. E. *et al.* Structure-dependent nonenzymatic deamidation of glutaminyl and asparaginyl pentapeptides. *J. Pept. Res.* **63**, 426–436 (2004).
57. Pales, L., Lambert, C. & Garcia, M.-A. *Atlas ostéologique pour servir à l'identification des mammifères du quaternaire*. (Editions du Centre national de la recherche scientifique, 1971-1981).
58. Schmid, E. *Atlas of Animal Bones: for Prehistorians, Archaeologists and Quaternary Geologists*. (Elsevier, 1972).
59. Smith, G. M. *et al.* Subsistence behavior during the Initial Upper Paleolithic in Europe: Site use,

dietary practice, and carnivore exploitation at Bacho Kiro Cave (Bulgaria). *J. Hum. Evol.* **161**, 103074 (2021).

60. Smith, G. M. Neanderthal megafaunal exploitation in Western Europe and its dietary implications: a contextual reassessment of La Cotte de St Brelade (Jersey). *J. Hum. Evol.* **78**, 181–201 (2015).

61. Fisher, J. W., Jr. Bone surface modifications in zooarchaeology. *J. Archaeol. Method Theory* **2**, 7–68 (1995).

62. Fernandez-Jalvo, Y. & Andrews, P. *Atlas of Taphonomic Identifications: 1001+ Images of Fossil and Recent Mammal Bone Modification*. (Springer Netherlands, 2016).

63. Binford, L. R. *Bones: Ancient Men and Modern Myths*. (Academic Press, 1981).

64. Blumenschine, R. J., Marean, C. W. & Capaldo, S. D. Blind tests of inter-analyst correspondence and accuracy in the identification of cut marks, percussion marks, and carnivore tooth marks on bone surfaces. *J. Archaeol. Sci.* **23**, 493–507 (1996).

65. Guérin, C. Première biozonation du Pléistocène Européen,principal résultat biostratigraphique de l'étude des Rhinocerotidae (Mammalia, Perissodactyla) du Miocène terminal au Pléistocène supérieur d'Europe Occidentale. *Geobios Mem. Spec.* **15**, 593–598 (1982).

66. Kahlke, R.-D. *The history of the origin, evolution and dispersal of the Late Pleistocene Mammuthus-Coelodonta faunal complex in Eurasia (large mammals)*. (Mammoth Site of Hot Springs, 1999).

67. Pederzani, S. *et al.* Subarctic climate for the earliest Homo sapiens in Europe. *Sci Adv* **7**, eabi4642 (2021).

68. Pederzani, S. *et al.* Early Homo sapiens dispersed into cold-arid steppes in central Europe. *Nature Ecology & Evolution* (2024).

69. Martisius, N. L. *et al.* Initial Upper Paleolithic bone technology and personal ornaments at Bacho Kiro Cave (Bulgaria). *J. Hum. Evol.* **167**, 103198 (2022).

70. Berto, C. *et al.* Environment changes during Middle to Upper Palaeolithic transition in southern Poland (Central Europe). A multiproxy approach for the MIS 3 sequence of Koziarnia Cave (Kraków-Częstochowa Upland). *Journal of Archaeological Science: Reports* **35**, 102723 (2021).

71. Behrensmeyer, A. K. Taphonomic and ecologic information from bone weathering. *Paleobiology* **4**, 150–162 (1978).
72. Fewlass, H. *et al.* Pretreatment and gaseous radiocarbon dating of 40–100 mg archaeological bone. *Scientific Reports* vol. 9 Preprint at https://doi.org/10.1038/s41598-019-41557-8 (2019).
73. Talamo, S., Fewlass, H., Maria, R. & Jaouen, K. 'Here we go again': the inspection of collagen extraction protocols for 14C dating and palaeodietary analysis. *Sci Technol Archaeol Res* **7**, 62–77 (2021).
74. van Klinken, G. J. Bone Collagen Quality Indicators for Palaeodietary and Radiocarbon Measurements. *J. Archaeol. Sci.* **26**, 687–695 (1999).
75. Guiry, E. J. & Szpak, P. Improved quality control criteria for stable carbon and nitrogen isotope measurements of ancient bone collagen. *J. Archaeol. Sci.* **132**, 105416 (2021).
76. Schwarcz, H. P. & Nahal, H. Theoretical and observed C/N ratios in human bone collagen. *J. Archaeol. Sci.* **131**, 105396 (2021).
77. Derrick, M. R., Stulik, D. & Landry, J. M. *Infrared Spectroscopy in Conservation Science*. (Getty Conservation Institute, 1999).
78. Bronk-Ramsey, C. Bayesian Analysis of Radiocarbon Dates. *Radiocarbon* **51**, 337–360 (2009).
79. Reimer, P. J. *et al.* The IntCal20 Northern Hemisphere Radiocarbon Age Calibration Curve (0–55 cal kBP). *Radiocarbon* **62**, 725–757 (2020).
80. Bronk-Ramsey, C. Dealing with Outliers and Offsets in Radiocarbon Dating. *Radiocarbon* **51**, 1023–1045 (2009).
81. Fewlass, H. *et al.* A 14C chronology for the Middle to Upper Palaeolithic transition at Bacho Kiro Cave, Bulgaria. *Nat Ecol Evol* **4**, 794–801 (2020).
82. Richter, D., Tostevin, G. & Skrdla, P. Bohunician technology and thermoluminescence dating of the type locality of Brno-Bohunice (Czech Republic). *J. Hum. Evol.* **55**, 871–885 (2008).
83. Škrdla, P., Nejman, L., Rychtaříková, T., Nikolajev, P. & Lisá, L. New observations concerning the Szeletian in Moravia: Neue Beobachtungen zum Szeletien in Mähren. *Quartär--Internationales Jahrbuch zur Erforschung des Eiszeitalters und der Steinzeit* **61**, 87–101 (2014).

84. Kot, M. *et al.* Chronostratigraphy of Jerzmanowician. New data from Koziarnia Cave, Poland. *J. Archaeol. Sci. Rep.* **38**, 103014 (2021).

85. Fu, Q. *et al.* Genome sequence of a 45,000-year-old modern human from western Siberia. *Nature* **514**, 445–449 (2014).

86. Welker, F. *et al.* Palaeoproteomic evidence identifies archaic hominins associated with the Châtelperronian at the Grotte du Renne. *Proc. Natl. Acad. Sci. U. S. A.* **113**, 11162–11167 (2016).

87. Hublin, J.-J. *et al.* Radiocarbon dates from the Grotte du Renne and Saint-Césaire support a Neandertal origin for the Châtelperronian. *Proc. Natl. Acad. Sci. U. S. A.* **109**, 18743–18748 (2012).

88. Balzeau, A. *et al.* Pluridisciplinary evidence for burial for the La Ferrassie 8 Neandertal child. *Sci. Rep.* **10**, 21230 (2020).

89. Devièse, T. *et al.* Reevaluating the timing of Neanderthal disappearance in Northwest Europe. *Proc. Natl. Acad. Sci. U. S. A.* **118**, (2021).

90. Peyrégne, S. & Peter, B. M. AuthentiCT: a model of ancient DNA damage to estimate the proportion of present-day DNA contamination. *Genome Biol.* **21**, 246 (2020).

91. Zavala, E. I. *et al.* Pleistocene sediment DNA reveals hominin and faunal turnovers at Denisova Cave. *Nature* **595**, 399–403 (2021).

92. Stoneking, M. Hypervariable sites in the mtDNA control region are mutational hotspots. *Am. J. Hum. Genet.* **67**, 1029–1032 (2000).

93. Tully, L. A. *et al.* A sensitive denaturing gradient-Gel electrophoresis assay reveals a high frequency of heteroplasmy in hypervariable region 1 of the human mtDNA control region. *Am. J. Hum. Genet.* **67**, 432–443 (2000).

94. Irwin, J. A. *et al.* Investigation of heteroplasmy in the human mitochondrial DNA control region: a synthesis of observations from more than 5000 global population samples. *J. Mol. Evol.* **68**, 516–527 (2009).

95. Weissensteiner, H. *et al.* HaploGrep 2: mitochondrial haplogroup classification in the era of high-throughput sequencing. *Nucleic Acids Res.* **44**, W58–63 (2016).

96. Katoh, K. & Standley, D. M. MAFFT multiple sequence alignment software version 7:

improvements in performance and usability. *Mol. Biol. Evol.* **30**, 772–780 (2013).

97. Bouckaert, R. *et al.* BEAST 2.5: An advanced software platform for Bayesian evolutionary analysis. *PLoS Comput. Biol.* **15**, e1006650 (2019).

98. Tamura, K. & Nei, M. Estimation of the number of nucleotide substitutions in the control region of mitochondrial DNA in humans and chimpanzees. *Mol. Biol. Evol.* **10**, 512–526 (1993).

99. Peyrégne, S. *et al.* Nuclear DNA from two early Neandertals reveals 80,000 years of genetic continuity in Europe. *Sci Adv* **5**, eaaw5873 (2019).

100. Fu, Q. *et al.* A revised timescale for human evolution based on ancient mitochondrial genomes. *Curr. Biol.* **23**, 553–559 (2013).

101. Baele, G., Li, W. L. S., Drummond, A. J., Suchard, M. A. & Lemey, P. Accurate model selection of relaxed molecular clocks in bayesian phylogenetics. *Mol. Biol. Evol.* **30**, 239–243 (2013).

102. Fu, Q. *et al.* DNA analysis of an early modern human from Tianyuan Cave, China. *Proc. Natl. Acad. Sci. U. S. A.* **110**, 2223–2227 (2013).

103. Higham, T. F. G., Bronk Ramsey, C., Brock, F., Baker, D. & Ditchfield, P. Radiocarbon dates from the oxford ams system: Archaeometry datelist 32. *Archaeometry* **49**, S1–S60 (2007).

104. Marom, A., McCullagh, J. S. O., Higham, T. F. G., Sinitsyn, A. A. & Hedges, R. E. M. Single amino acid radiocarbon dating of Upper Paleolithic modern humans. *Proc. Natl. Acad. Sci. U. S. A.* **109**, 6878–6881 (2012).

105. Krause, J. *et al.* A complete mtDNA genome of an early modern human from Kostenki, Russia. *Curr. Biol.* **20**, 231–236 (2010).

106. Kutschera, W. 4.4 Radiocarbon dating of the Iceman Ötzi with accelerator mass spectrometry. https://www2.chemistry.msu.edu/courses/CEM485/Lectures/IcemanAge.pdf.

107. Ermini, L. *et al.* Complete mitochondrial genome sequence of the Tyrolean Iceman. *Curr. Biol.* **18**, 1687–1693 (2008).

108. Rasmussen, M. *et al.* Ancient human genome sequence of an extinct Palaeo-Eskimo. *Nature* **463**, 757–762 (2010).

109. Gilbert, M. T. P. *et al.* Paleo-Eskimo mtDNA genome reveals matrilineal discontinuity in Greenland. *Science* **320**, 1787–1789 (2008).

110. Hedges, R. E. M., Pettitt, P. B., Ramsey, C. B. & Van Klinken, G. J. Radiocarbon dates from the oxford ams system: Archaeometry datelist 26. *Archaeometry* **40**, 437–455 (1998).

111. Fewlass, H. *et al.* Direct radiocarbon dates of mid Upper Palaeolithic human remains from Dolní Věstonice II and Pavlov I, Czech Republic. *Journal of Archaeological Science: Reports* **27**, 102000 (2019).

112. Trinkaus, E. *et al.* An early modern human from the Peştera cu Oase, Romania. *Proc. Natl. Acad. Sci. U. S. A.* **100**, 11231–11236 (2003).

113. Fu, Q. *et al.* An early modern human from Romania with a recent Neanderthal ancestor. *Nature* **524**, 216–219 (2015).

114. Benazzi, S. *et al.* Archaeology. The makers of the Protoaurignacian and implications for Neandertal extinction. *Science* **348**, 793–796 (2015).

115. Devièse, T. *et al.* Compound-specific radiocarbon dating and mitochondrial DNA analysis of the Pleistocene hominin from Salkhit Mongolia. *Nat. Commun.* **10**, 274 (2019).

116. Devièse, T. *et al.* Direct dating of Neanderthal remains from the site of Vindija Cave and implications for the Middle to Upper Paleolithic transition. *Proc. Natl. Acad. Sci. U. S. A.* **114**, 10606–10611 (2017).

117. Green, R. E. *et al.* A complete Neandertal mitochondrial genome sequence determined by high-throughput sequencing. *Cell* **134**, 416–426 (2008).

118. Hajdinjak, M. *et al.* Reconstructing the genetic history of late Neanderthals. *Nature* **555**, 652–656 (2018).
