# Supplementary material for: Homo sapiens reached the higher latitudes of Europe by 45,000 years ago
Source: Nature. 2024 Jan 31;626(7998):341–6. doi: 10.1038/s41586-023-06923-7 (PMC10849966; doi:10.1038/s41586-023-06923-7)
Supplement: Supplementary file 2 — Reporting Summary [file 41586_2023_6923_MOESM2_ESM.pdf]

nature portfolio

Corresponding author(s): Dr. M. Weiss, Prof. J.-J. Hublin

Last updated by author(s): Oct 25, 2023

Reporting Summary

Nature Portfolio wishes to improve the reproducibility of the work that we publish. This form provides structure for consistency and transparency in reporting. For further information on Nature Portfolio policies, see our Editorial Policies and the Editorial Policy Checklist.

Statistics

For all statistical analyses, confirm that the following items are present in the figure legend, table legend, main text, or Methods section.

n/a	Confirmed	
☐	☒	The exact sample size (*n*) for each experimental group/condition, given as a discrete number and unit of measurement
☒	☐	A statement on whether measurements were taken from distinct samples or whether the same sample was measured repeatedly
☐	☒	The statistical test(s) used AND whether they are one- or two-sided<br>*Only common tests should be described solely by name; describe more complex techniques in the Methods section.*
☒	☐	A description of all covariates tested
☐	☒	A description of any assumptions or corrections, such as tests of normality and adjustment for multiple comparisons
☐	☒	A full description of the statistical parameters including central tendency (e.g. means) or other basic estimates (e.g. regression coefficient) AND variation (e.g. standard deviation) or associated estimates of uncertainty (e.g. confidence intervals)
☐	☒	For null hypothesis testing, the test statistic (e.g. *F*, *t*, *r*) with confidence intervals, effect sizes, degrees of freedom and *P* value noted<br>*Give P values as exact values whenever suitable.*
☐	☒	For Bayesian analysis, information on the choice of priors and Markov chain Monte Carlo settings
☒	☐	For hierarchical and complex designs, identification of the appropriate level for tests and full reporting of outcomes
☒	☐	Estimates of effect sizes (e.g. Cohen's *d*, Pearson's *r*), indicating how they were calculated

Our web collection on statistics for biologists contains articles on many of the points above.

Software and code

Policy information about availability of computer code

Data collection	No specific software was used for data collection.
Data analysis	mMass (v. 5.5.0); Spectronaut (v. 15.6.211220); R (v. 4.1.2); OxCal (v.4.4); HaploGrep 2 (v. 2.4.0); MAFFT (v. 7.453); BEAST2 (v. 2.6.6); BWA(0.5.10), Samtools (v.1.3.1).

For manuscripts utilizing custom algorithms or software that are central to the research but not yet described in published literature, software must be made available to editors and reviewers. We strongly encourage code deposition in a community repository (e.g. GitHub). See the Nature Portfolio guidelines for submitting code & software for further information.

Data

Policy information about availability of data

All manuscripts must include a data availability statement. This statement should provide the following information, where applicable:

- Accession codes, unique identifiers, or web links for publicly available datasets
- A description of any restrictions on data availability
- For clinical datasets or third party data, please ensure that the statement adheres to our policy

The raw mass spectrometry LC-MS/MS proteomics data generated in this study have been deposited to the ProteomeXchange Consortium via the PRIDE partner repository under accession code PDX-043272. The raw mass spectrometry LC-MS/MS and MaxQuant search proteomics data for six bone specimens analyzed in DDA mode included in this study are deposited the ProteomeXchange Consortium via the PRIDE partner repository with the dataset identifier PXD-042321. The

MALDI-TOF .mzml and .msd type files included in this study are available in Zenodo (https://doi.org/10.5281/zenodo.8063812). The newly reconstructed mtDNA sequences are available on the DRYAD databases (https://doi.org/10.5061/dryad.1jwstqk0s). The sequencing data is available on the European Nucleotide Archive (PRJEB67776). Radiocarbon data is available in the supplementary information.

Research involving human participants, their data, or biological material

Policy information about studies with human participants or human data. See also policy information about sex, gender (identity/presentation), and sexual orientation and race, ethnicity and racism.

Reporting on sex and gender	NA
Reporting on race, ethnicity, or other socially relevant groupings	NA
Population characteristics	NA
Recruitment	NA
Ethics oversight	NA

Note that full information on the approval of the study protocol must also be provided in the manuscript.

Field-specific reporting

Please select the one below that is the best fit for your research. If you are not sure, read the appropriate sections before making your selection.

☐ Life sciences ☐ Behavioural & social sciences ☒ Ecological, evolutionary & environmental sciences

For a reference copy of the document with all sections, see nature.com/documents/nr-reporting-summary-flat.pdf

Ecological, evolutionary & environmental sciences study design

All studies must disclose on these points even when the disclosure is negative.

Study description	The Lincombian-Ranisian-Jerzmanowician (LRJ) is an archaeological technocomplex of the Middle to Upper Paleolithic transition spanning from Poland to Britain and whose makers are yet unknown. The main research questions of our project were: who made the LRJ and what is the chronology of their occupation? The site Ilsenhöle in Ranis is one of the type sites of the LRJ and the target of our investigations. The site was initially excavated from 1932-1938. The collection from this excavation period is stored in the Landesmuseum für Vorgschichte Halle/Saale, Germany. We excavated the site Ilsenhöhle in Ranis from 2016-2022. The goal was to clarify and date the stratigraphy, and obtain samples from the entire stratigraphy, with a focus on the LRJ find layer. These samples include: sediment samples, artefacts, bones, charcoal and sediment DNA. Additionally, we worked on the 1932-1938 collection and analysed the material. Based on the detailed stratigraphy of the new excavations and the dating, the material of old and new excavations can be correlated.
Research sample	Samples from archaeological fieldwork (sediment including micromorphology and sediment DNA, bones, charcoal, artefacts), and from the collection of the 1932-1938 excavation (bones).
Sampling strategy	For proteomic analysis, 20 mg of fine bone powder was homogenized and equally divided into two different tubes. For radiocarbon dating, samples were selected based on their stratigraphic context and presence of anthropogenic modifications (faunal bones) and human material from both collections was selected for direct dating. ~300-600 mg material was sampled from faunal bones and minimal sampling strategies were employed for the human remains (55-160 mg) to minimise destructive sampling. The amount for genetic analysis s between 9.9 and 63.9 mg per skeletal fragment.
Data collection	Data from the 2016-2022 fieldwork was collected during excavation. The 1932-1938 collection (bones) was provided from the Landesmuseum für Vorgschichte Halle/Saale, Germany.
Timing and spatial scale	Excavations of Ilsenhöhle in Ranis were conducted from 2016-2022. The sampling of the 1932-1938 collection (bones), stored in the Landesmuseum für Vorgeschichte Halle/Saale was done in parallel to the fieldwork.
Data exclusions	No data were excluded from this study.
Reproducibility	We performed two different types of proteomic analysis (Zooarchaeology by Mass spectrometry and shotgun proteomics) for species identification of a subset of bone specimens. However, not all specimens were analysed by the two different proteomic approaches.
Randomization	Randomization was not relevant to the current study - all available archaeological data were collectively analysed.
Blinding	Blinding was not relevant to the current study - all available archaeological materials were analysed.

Did the study involve field work? ☒ Yes ☐ No

Field work, collection and transport

Field conditions	The field conditions were optimal and temperate in terms of temperature. We excavated mostly during summer. Weather had only minimal effect as the site is a cave setting and was additionally protected by a roof.
Location	Ilsenhöhle in Ranis/ Thuringia, Germany: 50°39.7563'N, 11°33.9139'E
Access & import/export	We had direct access to the site Ilsenhöhle in Ranis and the Landesmuseum für Vorgeschichte/ Halle, as all responsible authorities are part of the study.
Disturbance	No disturbance

Reporting for specific materials, systems and methods

We require information from authors about some types of materials, experimental systems and methods used in many studies. Here, indicate whether each material, system or method listed is relevant to your study. If you are not sure if a list item applies to your research, read the appropriate section before selecting a response.

Materials & experimental systems

n/a	Involved in the study	
☒	☐	Antibodies
☒	☐	Eukaryotic cell lines
☐	☒	Palaeontology and archaeology
☒	☐	Animals and other organisms
☒	☐	Clinical data
☒	☐	Dual use research of concern
☒	☐	Plants

Methods

n/a	Involved in the study	
☒	☐	ChIP-seq
☒	☐	Flow cytometry
☒	☐	MRI-based neuroimaging

Palaeontology and Archaeology

Specimen provenance	Excavation of the site Ilsenhöhle in Ranis. Responsible: Thuringian State Office for the Preservation of Historical Monuments and Archaeology, Weimar.
Specimen deposition	Thuringian State Office for the Preservation of Historical Monuments and Archaeology, Weimar, Germany (2016-2022 collection); and Landesamt für Denkmalpflege und Archäologie Sachsen-Anhalt-Landesmuseum für Vorgeschichte, Halle, Germany (1932-1938 collection)
Dating methods	36 new radiocarbon dates are reported (bone = 27, charcoal = 3) from material sampled from the 1932-1938 collection and 2016-2022 collection. Human samples were selected for direct dating and faunal/charcoal material was selected from the new excavation based on stratigraphic context. Animal bones with anthropogenic modifications were selected where possible. ~300-600 mg material was sampled from faunal remains and 55-160 mg from human remains. Bone samples were pretreated at the MPI-EVA (Leipzig, Germany) using an acid-base-acid plus ultrafiltration protocol, with quality assessed based on collagen yield (%) and elemental values derived through EA-IRMS (C%, N%, C:N). Collagen extracts were graphitised and AMS dated at the Laboratory of Ion Beam Physics at ETH-Zurich (Switzerland; lab code ETH). Charcoal samples were pretreated with an ABOx pretreatment method, graphitised and AMS dated at the Curt-Engelhorn-Centre for Achaeometry (Mannheim, Germany; lab code MAMS). Two charcoal dates were excluded from the site model based on their low C% following combustion. All the dates were calibrated using the IntCal20 calibration curve (Reimer et al 2020). Calibration and modelling were done using OxCal (v4.4). All measured and calibrated dates are reported.

☒ Tick this box to confirm that the raw and calibrated dates are available in the paper or in Supplementary Information.

Ethics oversight	No ethical approval was required for this study considering we did not perform any experiments on living organisms.

Note that full information on the approval of the study protocol must also be provided in the manuscript.
